# Re-orienting coupling of organocuprates with propargyl electrophiles from S_N_2′ to S_N_2 with stereocontrol†
†Electronic supplementary information (ESI) available. See DOI: 10.1039/c6sc01086e


**DOI:** 10.1039/c6sc01086e

**Published:** 2016-05-10

**Authors:** Barry M. Trost, Laurent Debien

**Affiliations:** a Department of Chemistry Stanford University , Stanford , California 94305-5080 , USA . Email: bmtrost@stanford.edu

## Abstract

Diorganocuprate(i) reagents derived from lithiated heterocycles and CuCN react with enantioenriched secondary propargyl bromides to give the corresponding propargylated heterocycles. While propargyl electrophiles typically undergo S_N_2′ displacement, this transformation represents the first example of the reaction of hard carbanions with propargyl electrophiles in an S_N_2 fashion and occurs with excellent levels of stereoinversion. The new method was applied to the formal synthesis of (+)-frondosin B.

## Abstract 2

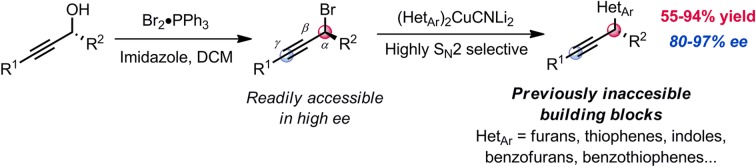
Diorganocuprate(i) reagents derived from lithiated heterocycles and CuCN react with enantioenriched secondary propagryl bromides to give the corresponding propargylated heterocycles.

## 

Nucleophilic organocopper(i) reagents are unambiguously invaluable synthetic tools for the chemo-, regio- and stereo-selective delivery of hard carbanions in a number of transformations such as carbocuprations, alkenylations or allylations.^1^ Regio- and stereo-selective organocopper(i) mediated substitutions of allylic electrophiles have been successfully developed to obtain either the S_N_2 or S_N_2′ adduct (eqn (a)).^2^ In contrast, substituted propropargylic electrophiles are only known to react in an S_N_2′ fashion^3^ to form the corresponding allene, regardless of the nature of the leaving group (R^2^ or R^3^ ≠ H, eqn (b)).^4^ Fu has pioneered a direct S_N_2 substitution of propargyl electrophiles by developing enantioselective nickel-catalyzed Negishi cross-couplings of propargyl bromides and carbonates with aromatic organozinc reagents (eqn (c)).^5^ However, the reactions with heteroaromatic nucleophiles have not been reported to date. Furthermore, in our hands, attempts to apply Fu's protocol to heterocyclic nucleophiles did not prove efficacious. The ubiquity of bioactive compounds bearing at least one heterocycle encouraged us to delineate a complementary protocol allowing the use of heteroaromatic nucleophiles that would solve this unanswered synthetic problem. Thus, we wondered whether a heteroaryl organocopper(i) reagent could be directed to react with propargyl electrophiles in an S_N_2 rather than the known S_N_2′ mode (eqn (d)).
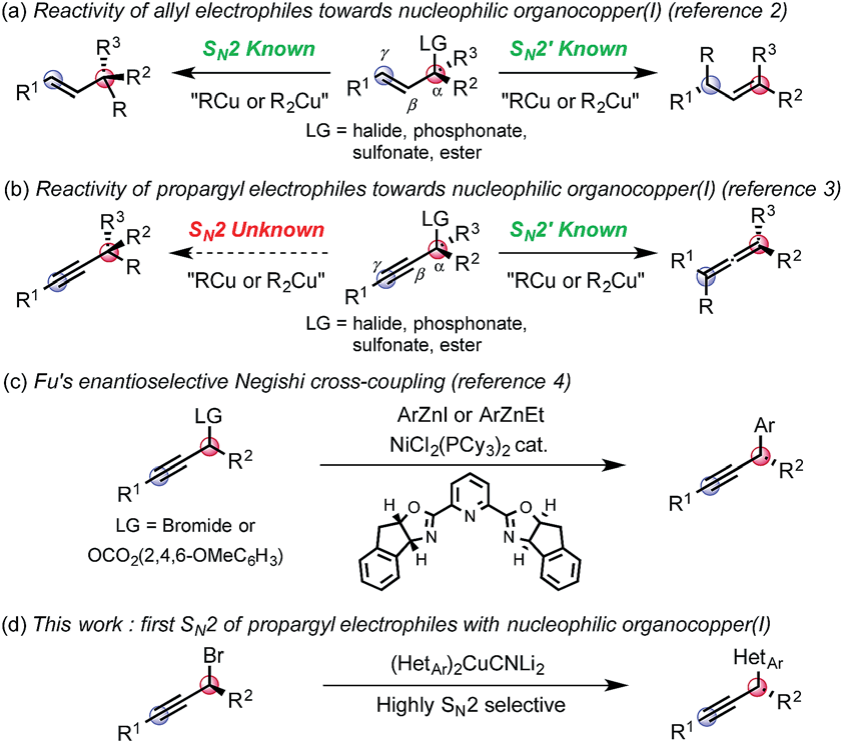


At the outset of our study, based on current knowledge about reaction mechanisms involving nucleophilic organocopper(i) reagents, we considered the mechanistic manifold depicted in Scheme 1 as a working hypothesis.^6^ We reasoned that the S_N_2′ selectivity observed for reactions between organocopper(i) reagents and propargyl electrophiles may be explained by the first step of the mechanism where coordination of the copper center to the triple bond triggers the oxidative addition step into the latter (Scheme 1, LG = leaving group, steps a and b). We thus hypothesized that blocking alkyne coordination could prevent oxidative addition *via* the π-system and direct it to occur at the C_α_–LG; ultimately opening-up a mechanistic path for the formation of the desired S_N_2 adduct (Scheme 1, bottom). Such a distinction may occur by adjusting the HOMO–LUMO energies since π-coordination requires favorable interactions with both the HOMO and the LUMO of the copper(i) complex while oxidative addition into the C_α_–LG only involves interaction with the HOMO. In practice, we anticipated that this differentiation could be achieved using coordinatively saturated organocuprates(i). The targeted transformation is nevertheless very challenging since, after oxidative addition into the C_α_–LG bond, the propargylcopper(iii) species generated could rapidly isomerize to the arguably more stable allenylcopper(iii).^7^ This would ultimately result in the formation of the undesired S_N_2′ allene product and/or racemization (Scheme 1).

**Scheme 1:**
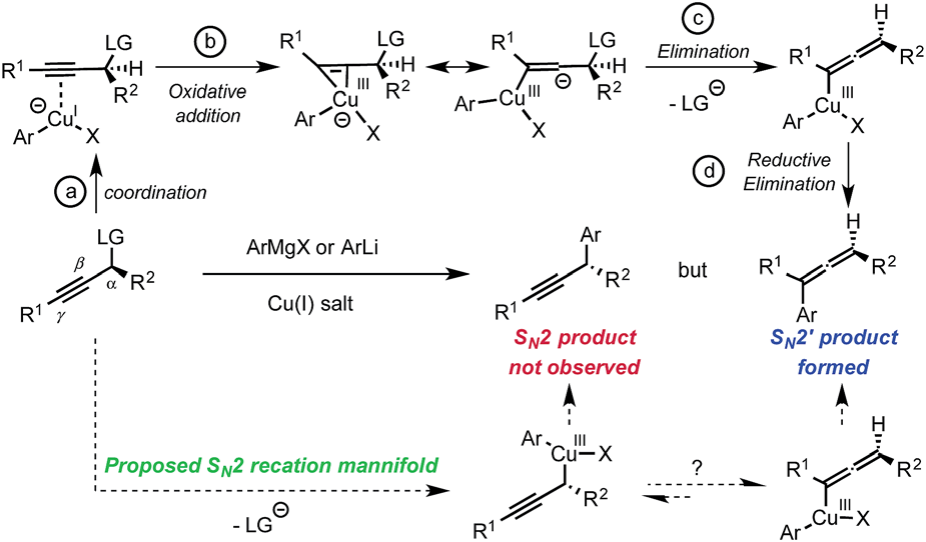
Proposed rationale for the formation of allenes.

With these considerations in mind, we began to explore the targeted reaction using furan **1a** in which the pendant alcohol would serve both as a directing group for site selective deprotonation and as a chelating group to saturate the coordination sphere of the copper. Previous studies on the displacement of aliphatic electrophiles with nucleophilic organocopper(i) reagents suggest that the S_N_2 reaction is more efficient in a polar solvent.^8^ Our investigation thus started by identifying the preferred leaving group using DME as a solvent and CuCN as the copper(i) salt (Table 1, entries 1–7). Commonly used leaving groups in the S_N_2′ displacement of propargyl electrophiles with organocopper(i) nucleophiles gave disparate results, both in terms of reactivity and selectivity. Thus, propargyl sulfonates were found to be superior substrates compare to propargyl phosphates to achieve the desired transformation (entries 1 and 5 *versus* 2 and 6). Aryl sulfonates and phosphates are more reactive than their aliphatic counterparts (entries 1 and 2 *versus* 5 and 6). Interestingly, the observed regio-selectivity seems to depend upon the nature of the leaving group (entries 1 to 6). The use of a perfluoroalkylated ester introduced by Dieter *et al.*4*c* resulted in partial conversion of the starting electrophile and formation of the desired product **2a** in moderate yield and selectivity (entry 4). In addition, a Troc leaving group performed poorly leading to a complex mixture with no desired product observed (entry 3). We then became intrigued about the possibility of using a bromide as leaving group. We found that a bromide acts as a good leaving group but also greatly impacts the selectivity towards the formation of the desired alkyne **2a** (entry 7). Various copper(i) salts were also rapidly surveyed and, amongst others, CuI was found to be as efficient as CuCN albeit with a drop in selectivity (entries 8 *versus* 7). On the other hand, CuSPh failed to deliver the desired alkyne (entry 9).

**Table 1:** Selected optimization studies^*a*^

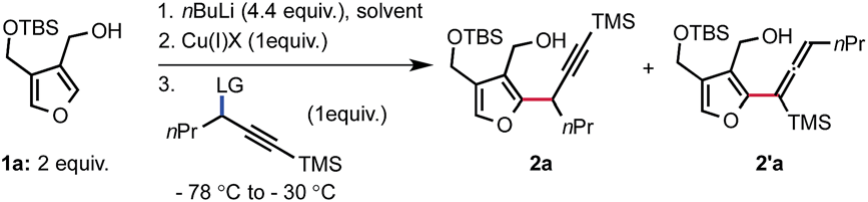					
Entry	Leaving group	Solvent	**2a**/**2′a**	% conv.^*a*^	% yield^*b*^
1	OMs	DME	70 : 30	82	44
2	OP(O)(OEt)_2_	DME	90 : 10	42	27
3	OTroc	DME	—	n.d.	0
4	OCOC_6_F_5_	DME	71 : 29	66	22
5	OSO_2_Ph	DME	75 : 25	100	61
6	OP(O)(OPh)_2_	DME	74 : 26	100	58
7	Br	DME	94 : 6	100	77
8^*b*^	Br	DME	89 : 11	100	68
9^*c*^	Br	DME	—	100	0
10	Br	THF	82 : 18	100	63
11	Br	Et_2_O	—	0	0
12^*d*^	Br	DME	—	0	0

^*a*^All reactions were performed on 0.5 mmol scale using CuCN (0.5 mmol).

^*b*^The reaction was run using CuI (0.5 mmol).

^*c*^The reaction was run using CuSPh (0.5 mmol).

^*d*^The reaction was performed in the absence of copper(i) salts.

Since the nature of the solvent is known to possess a strong effect on reactivity and selectivity in organocopper(i) chemistry, we evaluated alternative solvents. Running the displacement reaction in THF produced **2a** in diminished yield and selectivity. Conversely, using Et_2_O as a solvent resulted in the absence of reactivity (entry 11). Whitesides and co-workers previously observed that Et_2_O as a solvent can negatively impact the reactivity of organocopper(i) reagents towards alkyl electrophiles.^8^ Not surprisingly, reacting the organolithium reagent with the propargyl bromide in the absence of copper(i) salt resulted in the degradation of the reactants (entry 12).

With optimized conditions for controlling the regioselectivity of the heterocycle transfer (entry 7), we initiated the study of the enantioselectivity of the reaction by developing a facile synthesis of previously inaccessible enantioenriched propargyl bromides **4**. These are readily obtained in good yields *via* highly enantioselective Noyori reduction of ynones **3** followed by the one-pot activation and displacement of the alcohol in the presence of commercially available bromo(triphenyl)phosphonium bromide (Scheme 2).5*b* Noteworthy, under these conditions, the bromide substitution reactions occur with high levels of inversion of configuration. To our delight, under the conditions previously established (Table 1, entry 7), the reaction of the diorganocuprate(i) derived from **1a** with (*S*)-(3-bromohex-1-yn-1-yl)trimethylsilane **4a** (98% ee) delivered (*R*)-**2a** in 96% ee and a re-producible 77% yield. Further extension to a variety of enantioenriched propargyl bromides **4** was conducted to deliver enantioenriched propargyl heteroaryls **2** in good yields and with good levels of stereoinversion.

**Scheme 2:**
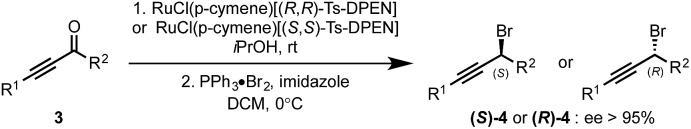
Synthesis of enantioenriched propargyl bromides.

Our study focused on diorganocuprates derived from readily accessible α-lithiathed heterocycles obtained *via* direct lithiation of the corresponding heterocyles. The results, compiled in Fig. 1 demonstrate the generality of the method. We initially observed that a carboxylate may be used in place of the alkoxide as a directing group without losing selectivity (example **2c**). We later found that no chelating group was necessary to achieve the desired transformation and that the displacement occurs with almost complete selectivity (s = S_N_2/S_N_2′ products ratio typically >95 : 5) when the nucleophile is metalated α- to the heteroatom. Thus, α-metalated furans (**2d**), benzofurans (**2e**), dihydrofurans (**2f**), thiophenes (**2g**, **i**, **m**), benzo-thiophenes (**2h**, **j**) and indoles (**2k**, **l**) are all competent nucleophiles in this transformation. It was intriguing to find that, despite the fact that thiophene typically acts as a “dummy” ligand in nucleophilic diorganocuprate(i) reactions, thiophenes and benzothiophenes are amongst the most efficient and most selective nucleophiles in this transformation (no allene formation observed). It is also worth noting that the excess nucleophile used in the transformation may be recovered after purification.^9^ Additionally, while using copper for inducing chirality practically requires the copper to be used catalytically, stoichiometric copper is not a limitation in processes involving achiral copper complexes.^10^ As for the electrophilic partner **4**, the reaction is particularly efficient with TMS-protected alkynes,^11^ which is ideal in view of subsequent functionalization. The reaction is also efficient with R^1^ substituents being aromatic (**2b**, **m**) and BDMS groups (**2j**). As recently illustrated in our synthesis of leustrodusin B,^12^ the BDMS alkynes can serve as useful vinyl cross-coupling partners after semi-hydrogenation of the triple bond; even in complex settings. A number of useful functional groups such as alkenes (**2d**, **k**), arenes (**2b**, **e**, **f**, **m**), protected alcohols (**2h**) and chlorides (**2i**, **l**) are perfectly tolerated substituents. Interestingly an aryl bromide (**2b**) is compatible with the mild reaction conditions. In addition, this stereo-selective displacement is also efficient when the starting bromide **4** possess a secondary center adjacent to the reactive carbon center such as an iso-propyl group (**2g**). Overall, the scope of the transformation compares favorably to the Negishi cross-couplings developed by Fu.^5^ As illustrated in Fig. 1, both enantiomers are of equal ease to access using our strategy and the levels of enantioselectivity achieved are good. Furthermore, while the reactions were routinely run on a 1 mmol scale, we successfully utilized our protocol on up to 50 mmol scale.

**Fig. 1:**
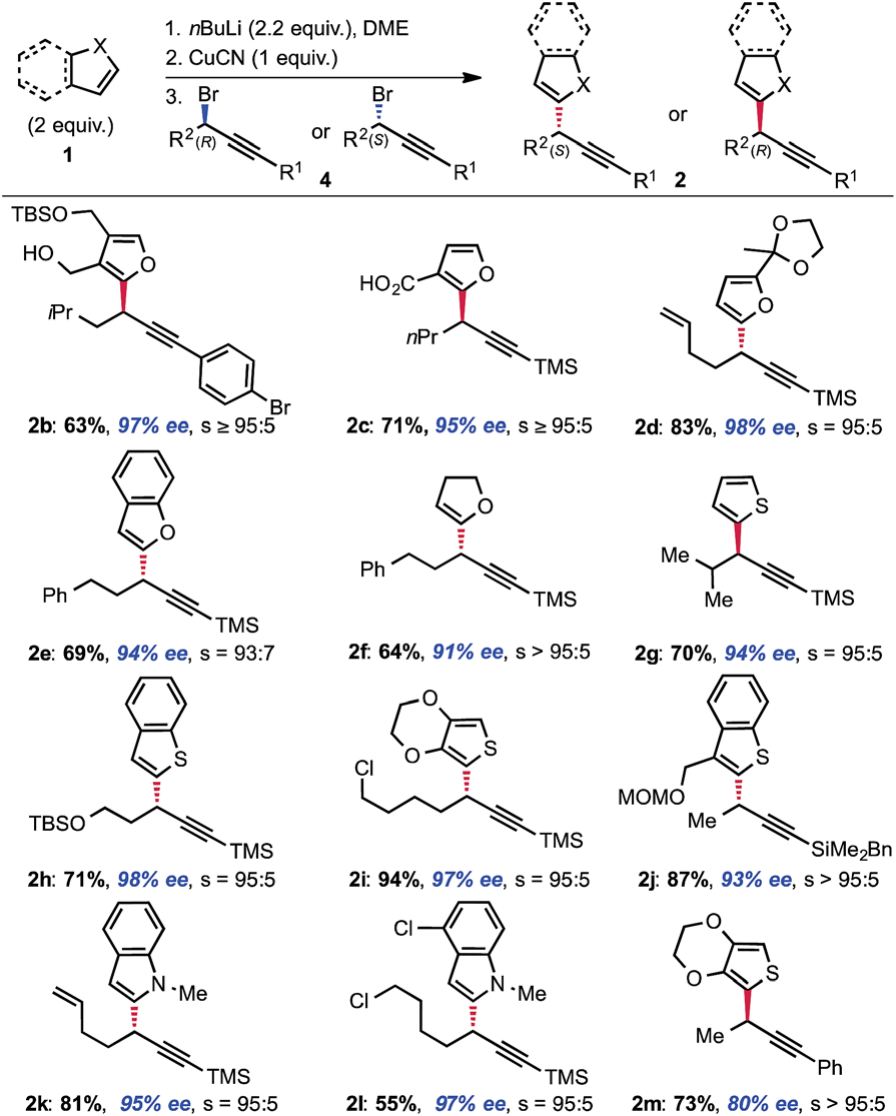
Scope of the copper-mediated displacement.^*a a*^All reactions were performed on 1 mmol scale. Yields and selectivities were calculated on average of two runs: one with the racemic bromide and one with the enantioenriched bromide.

As nicely highlighted in Jarvo and co-workers' recent work;^13^ in the context of drug discovery, the strategic introduction of a methyl group can improve drug potency through various mechanisms in the hit to lead approach. It is thus interesting to observe that tertiary benzylic stereocenters bearing a methyl group are common motifs in medicines. In particular, such a motif is present in a number of heteroaromatic bioactive compounds with varied activities (Fig. 2). Development of stereoselective methods to obtain these compounds are relevant both from a synthetic and a practical point of view since the different enantiomers are known to possess differential bioactivity. In addition, methods to access these motifs are still scarce. We decided to probe further the scope of the transformation towards the synthesis of such privileged building blocks. Example **2j** and **2m** (Fig. 1), already hinted to the possibility of using our newly designed strategy for their synthesis.

**Fig. 2:**
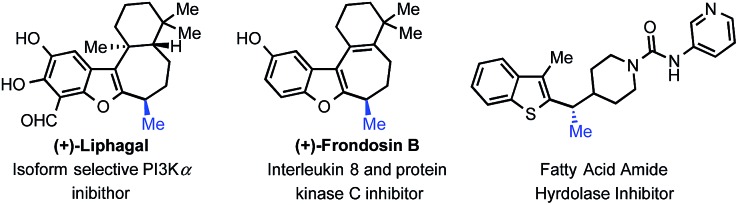
Bioactive compounds with a benzylic methyl group.

Interestingly, molecular complexity may be rapidly built by combining our displacement reaction with other transformations. As shown in Scheme 3, methyllithium can be added to 3-furan-carboxaldehyde and the corresponding alkoxide can be used as a directing group for metallation. In spite of the proximal presence of the racemic alkoxide, no effect on the enantioselectivity was observed. Thus, transmetallation to CuCN and treatment with (*S*)-(3-bromobut-1-yn-1-yl)trimethylsilane **4b** followed by Dess–Martin oxidation of the corresponding alcohol **2n** delivered the doubly functionalized methylketone **5a** in 68% yield and 95% ee. Alternatively, we found that the displacement reaction is selective for propargyl bromides over other electrophiles that can in turn be functionalized. Thiophene **2o**, was obtained using this strategy. This approach allows for further functionalization of the S_N_2 adduct *via* the subsequent transformation of the sulfonate as illustrated by the preparation of azide **5b** (Scheme 3). The selectivity of the displacement reaction for the propargyl bromide over other electrophiles such as primary alkylchlorides and arylsulfonates is particularly noteworthy. Indeed, even secondary alkylhalides and arylsulfonates are known to undergo S_N_2 displacement with diorganocuprate(i) reagents at low temperatures.^14^

**Scheme 3:**
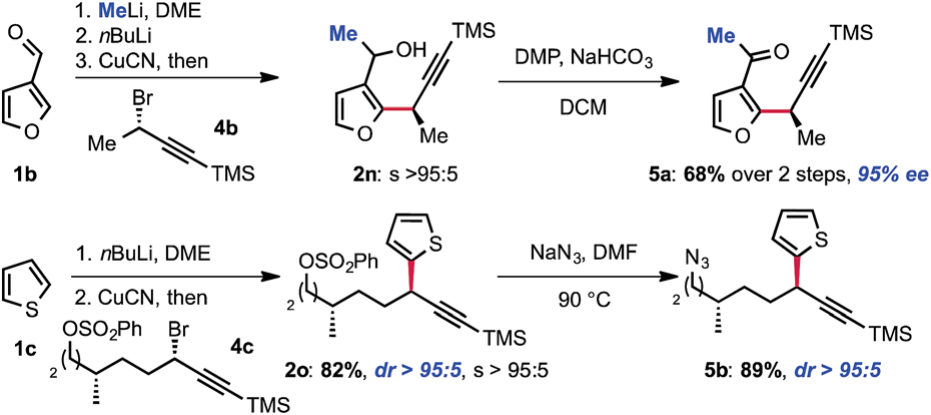
Modular assembly of functionalized scaffolds.

Having established this methodology, we wanted to demonstrate its potential for natural product synthesis. The synthetic utility of the novel displacement was thus further demonstrated by the rapid assembly of aldehyde **5c** that was used as a key intermediate in the synthesis of (+)-frondosin B.^15^ Thus, applying our protocol, alkyne **2p** was obtained in 79% yield, 96% ee and with full S_N_2 selectivity. Deprotection of the TMS-alkyne with TBAF/AcOH followed by hydroboration and oxidation of the intermediate vinylborane, delivered the targeted enantioenriched aldehyde **5c** in good yield over 3 steps, completing the formal synthesis of (+)-frondosin B (Scheme 4). The absolute configuration of intermediate **5c** was confirmed by measurement of its specific rotation, which was comparable to the value obtained previously.^15^ This means that the displacement occurs with Walden inversion at the electrophilic carbon bearing the bromide.^16^

**Scheme 4:**
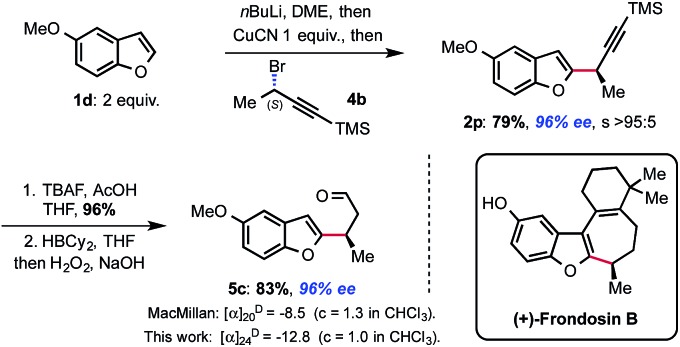
Short formal synthesis of (+)-frondosin B.

While the origin of the observed S_N_2 selectivity and high stereoinversion have not yet been elucidated, a scenario in which: (i) the diorganocopper(i) oxidatively adds into the C_α_–Br bond and (ii) the resulting copper(iii) species is undergoing rapid reductive elimination is plausible. In this context, it also worth noting that our attempt to react diarylcopper(i) nucleophiles with propargyl bromide **4a** was poorly regioselective and resulted in the obtention of mixtures of the desired alkynes **2** together with allenes **2′**.^17^ These additional observations suggest that the presence of the heteroatom α- to the copper center is important to achieve the desired selectivity. In 2012, Nakamura published guidelines for the structure/reactivity relationship of nucleophilic organocopper(i) reagents based on orbital analysis.^6^ Following this analysis, a linear geometry at copper would inhibit alkyne coordination and favor oxidative addition into the C_α_–Br bond (Scheme 5). The presence of lithium cations could additionally increase the rate of oxidative addition into the C_α_–Br bond by coordination of the lithium to the bromine atom.^18^ Overall, we propose that the high affinity of the heteroatoms (O, S, N) for lithium invoked to explain the behavior of “dummy” ligands,^19^ might favor the linear geometry for the diorganocuprate(i) intermediates and would ultimately account for the observed reactivity. A putative simplified structure for the diheteroarylcuprate(i) reagents is distorted square planar (Scheme 5). It is also interesting to note that, in the case of adduct **2f**, the organo-cuprate resulting from oxidative addition undergoes reductive elimination faster than the Kocieński style migratory insertion.^20^

**Scheme 5:**
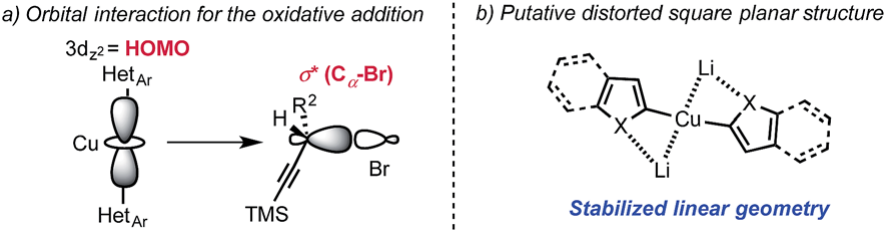
Putative explanation for the observed reactivity.

In summary, we have developed the first example of a selective S_N_2 displacement of propargyl electrophiles with hard carbanions employing readily accessible diorganocuprate(i) reagents and enantioenriched propargyl bromides. This transformation is particularly selective with heterocyclic nucleophiles, occurs with stereoinversion at the C_α_sp^3^ center, and leads to versatile enantioenriched alkynes. The success of the copper mediated reaction in generating highly enantioenriched propargylic centers represents a useful complement to the nickel-catalyzed protocol that failed when attempted using the reported optimized conditions. In addition, the reaction scope compares favorably to the nickel catalyzed Negishi coupling. The synthetic utility of this novel transformation was illustrated by a short formal synthesis of (+)-frondosin B. We expect this transformation to offer new synthetic opportunities not hitherto conceivable. Extension of this method to other types of nucleophiles and in particular to standard aromatic nucleophiles is underway and will be reported in due course.

## Supplementary Material

Supplementary information
