# Supplementary material for: Re-orienting coupling of organocuprates with propargyl electrophiles from SN2′ to SN2 with stereocontrol
Source: Chem Sci. 2016 May 10;7(8):4985–9. doi: 10.1039/c6sc01086e (PMC5207346; doi:10.1039/c6sc01086e)
Supplement: Supplementary file 1 [file SC-007-C6SC01086E-s001.pdf]

Electronic Supplementary Material (ESI) for Chemical Science.
This journal is © The Royal Society of Chemistry 2016

Electronic Supplementary Information

Re-Orienting Coupling of Organocuprates with Propargyl Electrophiles from S_N2' to S_N2 with Stereocontrol.

Barry M. Trost* and Laurent Debien

Department of Chemistry Stanford University, Stanford, California 94305-5080, United States.

bmtrost@stanford.edu

Contents:

General Experimental Methods……………………………………………………………………………*S2*
List of compounds : characterization and spectra pages……………………………………*S3-S4*
Experimental Procedures and Spectroscopic Data………………………………………*S5-S19*
Copies of ^{1}H and ^{13}C Spectra………………………………………………………………*S19-S52*

General Experimental Methods

Air and/or moisture sensitive reactions were carried out under an argon atmosphere in oven-dried glassware and with anhydrous solvents. All compounds were purchased from commercial sources unless otherwise noted and used without further purification. Solvents were freshly distilled or dried by passing through an alumina column. 1,4-dioxane was distilled over sodium benzophenone under a nitrogen atmosphere. Thin layer chromatography was carried out on glass plates coated with silica gel SiO2 60 F254 from Merck; visualization with a UV lamp (254 nm) or by staining with a p-anisaldehyde or a vanilin solution. Flash chromatography was performed with silica gel SiO2 60 (0.040–0.063 µm, 230–400 mesh), technical solvents, and a head pressure of 0.2–0.4 bar. Melting points (m.p.) were measured on a Thomas Hoover capillary melting point apparatus in open capillaries and are uncorrected. Proton (1H) and carbon (13C) nuclear magnetic resonance (NMR) spectroscopy was performed on a Mercury NMR instrument at 400 MHz (1H) or 100 MHz (13C) and on a Unity NMR spectrometer at 500 MHz (1H) or 125 MHz (13C). Chemical shifts are reported in ppm relative to the residual protiated solvent (CDCl3: δH = 7.26 ppm, δC = 77.0 ppm). All 13C NMR spectra are proton decoupled. The resonance multiplicity is described as s (singlet), d (doublet), t (triplet), q (quartet), m (multiplet), and br (broad). Infrared spectroscopic (IR) data were recorded on sodium chloride plates neat or as thin films on a Perkin-Elmer Paragon 500 FT-IR spectrometer. Absorption bands are reported in wavenumbers (cm−1) in the range of 4000–1000 cm−1 . High-resolution mass spectrometry (HRMS) was measured on a Bruker micrOTOF-Q II electronspray ionization (ESI) mass spectrometer by the Vincent Coates Foundation Mass Spectrometry Laboratory at Stanford University. Mass peaks are reported in m/z units.

List of starting propargyl bromides 4 : characterization and spectra pages

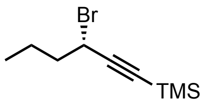

4a
Characterization: S6
Spectra: S20

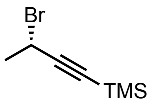

4b
Characterization: S6
Spectra: S21

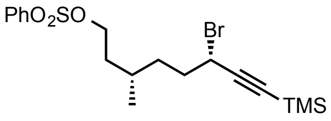

4c
Characterization: S6
Spectra: S22-23

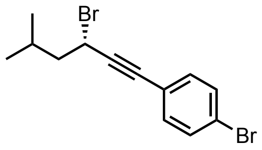

4d
Characterization: S7
Spectra: S24

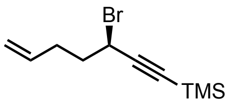

4e
Characterization: S8
Spectra: S25

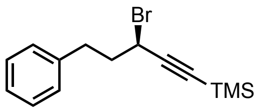

4f
Characterization: S8
Spectra: S26

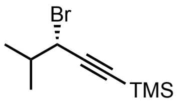

4g
Characterization: S8
Spectra: S27

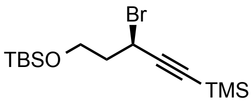

4h
Characterization: S9
Spectra: S228

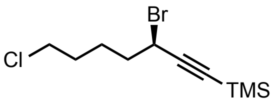

4i
Characterization: S9
Spectra: S29

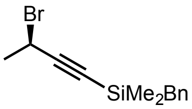

4j
Characterization: S10
Spectra: S30

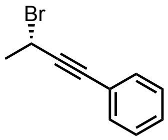

4k
Characterization: S10
Spectra: S31

List of final products 2 and 5 : characterization and spectra pages

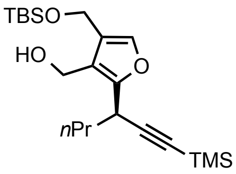

2a
Characteriation: S11
Spectra: S32

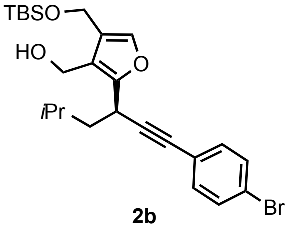

2b
Characteriation: S11
Spectra: S33

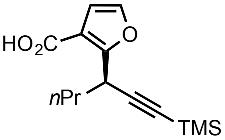

2c
Characteriation: S12
Spectra: S34

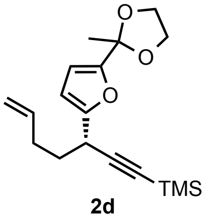

2d
Characteriation: S12
Spectra: S35

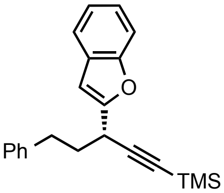

2e
Characteriation: S13
Spectra: S36

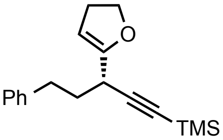

2f
Characteriation: S13
Spectra: S37

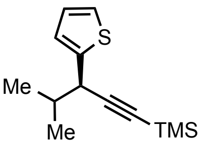

2g
Characteriation: S13
Spectra: S38

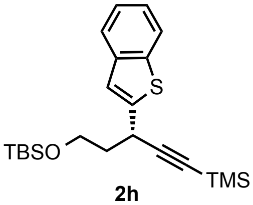

2h
Characteriation: S14
Spectra: S39

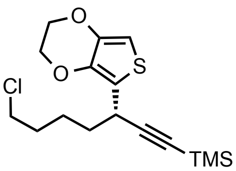

2i
Characteriation: S14
Spectra: S40

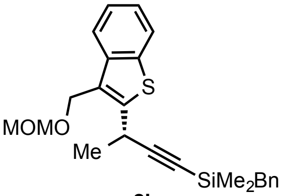

2j
Characteriation: S15
Spectra: S41

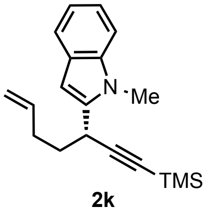

2k
Characteriation: S15
Spectra: S42

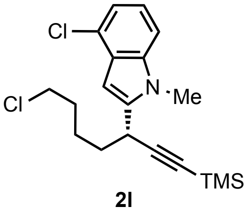

2l
Characteriation: S16
Spectra: S43

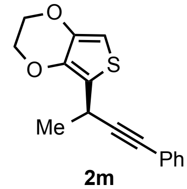

2m
Characteriation: S16
Spectra: S44

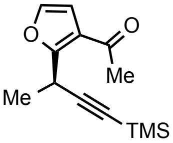

5a
Characteriation: S17
Spectra: S45

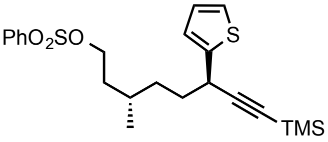

2o
Characteriation: S17
Spectra: S46-47

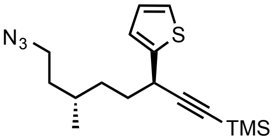

5b
Characteriation: S17
Spectra: S48-49

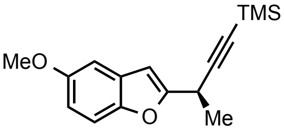

2p
Characteriation: S18
Spectra: S50

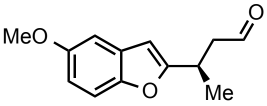

5c
Characteriation: S19
Spectra: S52

General procedure A for the preparation of the enantioenriched propargyl alcohol

A mixture of freshly distilled *iso*-propanol[1] (3n mL) and ynone **3** (n mL) was degassed by bubbling of $Ar_{(g)}$ during 15 minutes. At this point RuCl(p-cymene)[(*R*,*R*)-Ts-DPEN] or RuCl(p-cymene)[(*R*,*R*)-Ts-DPEN] (1 mol%) was added and the resulting mixture was stirred for 6 hours. Additional portions of catalyst were added every 6 hours until full conversion of the starting ynone as determined by TLC analysis. The crude mixture was evaporated to dryness and the residual oil was purified by chromatography over silica gel to afford the enantioenriched propargyl alcohol.

General procedure B for the preparation of the propargyl bromides 4[2]

A round bottom flask was charged with triphenylphosphine (1.2n mmol) and DCM (2n mL). The mixture was cooled to 0°C under argon and bromine (1.2n mmol) was added dropwise. A yellow pale precipitate formed and the reaction mixture was stirred for an additional 30 minutes. A separate round bottom flask was charged with the propargyl alcohol (n mmol), imidazole (1.2n mmol) and DCM (2n mL) and the mixture was cooled to 0°C under argon. The bromotriphenylphosphonium bromide suspension was added dropwise to the alcohol solution and the mixture was warmed to room temperature over 3 hours. Silica (1.5n mL) was added to the and the mixture was evaporated to dryness and purified by column chromatography over silica gel (pentane:ether) to afford the desired propargyl bromide **4**.

General procedure C for the S_N2 displacement reaction and formation of 2[3]

A flamed dried flask was charged with the heterocycle **1** of choice (2n mmol) and freshly distilled DME (6n mmol) and the solution was cooled to -78°C under argon. *n*BuLi (2.5 M in hexanes, 2.2n mmol) was then added dropwise and the resulting solution was stirred for 1.5 hours. $CuCN_{(s)}$ (n mmol) was the added and the resulting suspension was then allowed to warm up to -40°C over 1.5 hours. The mixture was then cooled back to -78°C and the propargyl bromide electrophile **4** was added via syringe. The reaction mixture was stirred between -78°C and -40°C over 3 hours and then quenched with a saturated ammonium chloride solution. The mixture was diluted with diethyl ether and after standard work up ($NH_4Cl_{(sat.)}$, $NaCl_{(sat.)}$), the combined organic layers were dried over anhydrous sodium sulfate and evaporated to dryness. The residual oil was then purified by column chromatography over silica gel to afford the desired alkyne **2**.

---

[1] *Iso*-propanol was distilled over $CaH_{2(s)}$ under $Ar_{(g)}$.

[2] The enantiomeric excess of the propargyl bromides **4** could not always be determined either by chiral GC or HPLC analyses. In these cases, the enantiomeric excess of the propargyl bromide **4** was considered to be identical to the one of the starting propargyl alcohol.

[3] All the reaction temperature given are cold bath temperature. All reactions were run twice: once with the racemic and once with the enantioenriched bromide **4**.

(*S*)-(3-bromohex-1-yn-1-yl)trimethylsilane 4a

(*R*)-1-(trimethylsilyl)hex-1-yn-3-ol (8.52 g, 50 mmol ,98% ee) was prepared according to general procedure **A**. It was engaged in the next step following general procedure **B** using triphenyl phosphine (1.57 g), bromine (3.07 mL) and imidazole (4.08 g). Purification by chromatography over silica gel (pentane) afforded the desired bromide **4a** (10.15 g, 87%, 98% ee).

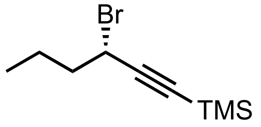

^{1}H NMR ($CDCl_3$, 400 MHz, δ (ppm)) 4.51 (t, *J* = 7.0 Hz, 1H), 2.03-1.92 (m, 2H), 1.61-1.46 (m, 2H), 0.95 (t, *J* = 7.4 Hz, 3H), 0.17 (s, 9H). **^{13}C NMR** ($CDCl_3$, 125 MHz, δ (ppm)) 103.9, 91.7, 41.6, 37.1, 20.6, 13.2, 0.3. **IR (thin film)** ν = 2920, 2861, 2834, 2143, 1444, 1233. **$[\alpha]_{25}^{D}$** = -10.6 (c = 1.0 in $CHCl_3$). **Chiral GC**: Cyclodex-B 90 C, 1C.min^{-1} t_R = 15.1 (minor), 15.6 (major). **HRMS** Calcd for $C_9H_{17}BrSi+NH_4^+$: 250.0621, found 250.0627.

(*S*)-(3-bromobut-1-yn-1-yl)trimethylsilane 4b[4]

(*R*)-4-(trimethylsilyl)but-3-yn-2-ol (3.55 g, 25 mmol , 98% ee) was prepared according to general procedure **A**. It was engaged in the next step following general procedure **B** using triphenyl phosphine (7.87 g), bromine (1.54 mL) and imidazole (2.04 g). Purification by chromatography over silica gel (pentane) afforded the desired bromide **4b** (3.95 g, 77%).

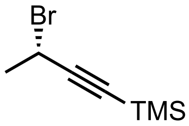

^{1}H NMR ($CDCl_3$, 400 MHz, δ (ppm)) 4.60 (q, *J* = 6.8 Hz, 1H), 1.89 (d, *J* = 6.8 Hz, 1H), 0.17 (s, 9H). **^{13}C NMR** ($CDCl_3$, 125 MHz, δ (ppm)) 104.9, 91.0, 31.3, 27.3, -0.3. **IR (thin film)** ν = 2919, 2138, 1423, 1355, 1233, 1166, 1089. **$[\alpha]_{25}^{D}$** = -18.1 (c = 1.0 in $CHCl_3$). **MS** Calcd for $C_9H_{17}BrSi-Br^{\cdot}+NH_4^+$: 143.1, found: 143.1.

(3*S*,6*S*)-6-bromo-3-methyl-8-(trimethylsilyl)oct-7-yn-1-ylbenzenesulfonate 4c

(*S*)-6-hydroxy-4-methylhexanal[5] (2.60 g, 20 mmol) in DCM (50 mL) was added triethylamine (4.05 mL, 30 mmol) and benzene sulfonylchloride (3.53 g, 20 mmol). After full conversion, the reaction mixture was worked-up and the residue was purified by chromatography over silica gel to afford (*S*)-3-methyl-6-oxohexyl benzenesulfonate (5.30 g, 98 %).

To a -78°C solution of lithium trimethylsilylacetylene in THF (50 mL) formed from trimethylsilyl acetylene (3.96 mL, 28 mmol) and *n*BuLi (2.5M in hexanes, 10 mL, 25 mmol) was added the freshly prepared aldehyde (5.14 g, 19 mmol)in THF (2x6 mL).

---

[4] The ee of the bromide could not be measured and was assumed to be identical to the ee reported for the starting alcohol ee = 98%, see: Matsumura, K.; Hashiguchi, S.; Ikariya, T.; Noyori, R. *J. Am. Chem. Soc.* **1997**, *119*, 8738.

[5] (*S*)-6-hydroxy-4-methylhexanal was prepared according to the literature protocol, see: Sheng, G.; Jie, L.; Dawei, M. *Angew. Chem., Int. Ed.* **2015**, *54*, 1298.

The reaction medium was warmed to -20°C over 1 hour and quenched with $NH_4Cl_{(sat.)}$. After usual work up the residue was purified by chromatography over silica gel to afford (3*S*)-6-hydroxy-3-methyl-8-(trimethylsilyl)oct-7-yn-1-yl benzenesulfonate (6.44 g, 92%) as a one to one mixture of diastereoisomers.
The previously obtained mixture of diastereoisomers (4.42 g, 12 mmol) was dissolved in DCM (30 mL). Celite (10.3 g) and PCC (10.3 g, 48 mmol) and the resulting mixture was stirred until full conversion. It was then purified by chromatography over silica gel to afford (*S*)-3-methyl-6-oxo-8-(trimethylsilyl)oct-7-yn-1-yl benzenesulfonate (3.61g, 82%).
(3*S*,6*R*)-6-hydroxy-3-methyl-8-(trimethylsilyl)oct-7-yn-1-yl benzenesulfonate (3.26 g, 8.84 mmol , 93%) was prepared as a unique diastereoisomer from (*S*)-3-methyl-6-oxo-8-(trimethylsilyl)oct-7-yn-1-yl benzenesulfonate (3.50 g, 9.5 mmol) according to general procedure **A**. It was engaged in the next step following general procedure **B** using triphenyl phosphine (2.78 g), bromine (543 µL) and imidazole (722 mg). Purification by chromatography over silica gel afforded the desired bromide **4c** as a unique diastereoisomer (3.36 g, 88%).

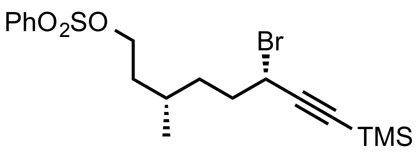

^{1}H NMR ($CDCl_3$, 500 MHz, δ (ppm)) 7.93-7.89 (m, 2H), 7.67-7.62 (m, 1H), 7.58-7.53 (m, 2H), 4.42 (t, *J* = 6.8 Hz, 1H), 4.14-4.05 (m, 2H), 1.99-1.81 (m, 2H), 1.73-1.65 (m, 1H), 1.63-1.53 (m, 1H), 1.49-1.40 (m, 2H), 1.37-1.25 (m, 1H), 0.84 (d, *J* = 6.5 Hz, 3H), 0.17 (s, 9H). **^{13}C NMR** ($CDCl_3$, 125 MHz, δ (ppm)) 136.1, 133.7, 129.2, 127.8, 103.5, 92.1, 68.8, 37.3, 36.8, 35.5, 33.9, 28.5, 19.0, -0.3. **$[\alpha]_{25}^{D}$** = -5.3 (c = 1.0 in $CHCl_3$).

(S)-1-bromo-4-(3-bromo-5-methylhex-1-yn-1-yl)benzene 4d[6]

(*R*)-1-(4-bromophenyl)-5-methylhex-1-yn-3-ol (1.50 g, 5.6 mmol, 97% ee) was prepared according to general procedure **A**. It was engaged in the next step following general procedure **B** using triphenyl phosphine (1.76 g), bromine (344 µL) and imidazole (457 mg). Purification by chromatography over silica gel (pentane/diethyl ether, 95:5) afforded the desired bromide **4d** (1.75 g, 95%).

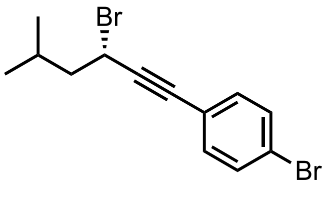

^{1}H NMR ($CDCl_3$, 400 MHz, δ (ppm)) 7.47-7.42 (m, 2H), 7.32-7.27 (m, 2H), 4.75 (t, *J* = 7.6 Hz, 1H), 2.04-1.90 (m, 3H), 0.98 (d, *J* = 6.4 Hz, 3H), 0.97 (d, *J* = 6.4 Hz, 3H). **^{13}C NMR** ($CDCl_3$, 100 MHz, δ (ppm)) 133.2, 131.6, 123.0, 121.2, 89.4, 85.6, 48.6, 36.0, 26.9, 21.9, 21.9. **IR (thin film)** ν = 3046, 2910, 2858, 2827, 2199, 1464, 1447, 1373, 1364, 1346, 1323, 1302, 1268, 1241, 1150, 1111. **$[\alpha]_{25}^{D}$** = -6.8 (c = 1.0 in $CHCl_3$). **MS** Calcd for $C_{13}H_{14}Br_2$-HBr: 248.0, found: 248.2.

[6] The ee of the bromide could not be measured and was assumed to be identical to the ee of the starting alcohol (ee = 97%, **Chiral HPLC**: Chiralpak® IC, 2% isopropanol in heptane, 0.8 mL.min^{-1}, λ = 254 nm, t_R = 11.5 (minor), 12.2 (major).

(*R*)-(3-bromohept-6-en-1-yn-1-yl)trimethylsilane 4e

(*S*)-1-(benzyldimethylsilyl)hept-6-en-1-yn-3-ol (1.64 g, 9 mmol ,99% ee) was prepared according to general procedure **A**. It was engaged in the next step following general procedure **B** using triphenyl phosphine (2.83 g), bromine (553 µL) and imidazole (753 mg). Purification by chromatography over silica gel (pentane) afforded the desired bromide **4e** (1.90 g, 86%, 99% ee).

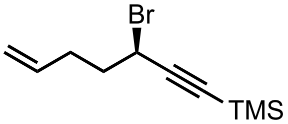

^{1}H NMR ($CDCl_3$, 400 MHz, δ (ppm)) 5.84-5.70 (m, 1H), 5.12-5.05 (m, 1H), 50.5-5.0 (m, 1H), 4.50 (t, *J* = 6.8 Hz, 1H), 2.35-2.23 (m, 2H), 2.13-2.02 (m, 2H), 0.18 (s, 9H). **^{13}C NMR** ($CDCl_3$, 100 MHz, δ (ppm)) 136.3, 116.1, 103.6, 94.4, 92.2, 38.6, 36.5, 31.4, -0.3. **IR (thin film)** ν = 3036, 2919, 2861, 2142, 1619, 1424, 1395, 1233, 1147. **$[\alpha]_{25}^{D}$** = -0.5 (c = 1.0 in $CHCl_3$). **Chiral GC**: Cyclodex-B 90 C, 1C.min^{-1} t_R = 18.3 (major), 18.6 (minor). **MS** Calcd for C10H17BrSi-Br·+NH_4^+: 183.1, found: 183.1.

(*R*)-(3-bromo-5-phenylpent-1-yn-1-yl)trimethylsilane 4f[7]

(*S*)-5-phenyl-1-(trimethylsilyl)pent-1-yn-3-ol (2.05g, 8.8 mmol , 99% ee) was prepared according to general procedure **A**. It was engaged in the next step following general procedure **B** using triphenyl phosphine (2.77 g), bromine (541 µL) and imidazole (719 mg). Purification by chromatography over silica gel (pentane/diethyl ether, 95:5) afforded the desired bromide **4f** (2.29 g, 88%).

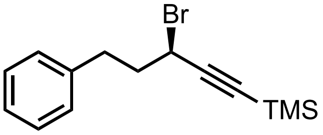

^{1}H NMR ($CDCl_3$, 500 MHz, δ (ppm)) 7.33-7.27 (m, 2H), 7.24-7.29 (m, 3H), 4.44 (t, *J* = 7.0 Hz, 1H), 2.90-2.79 (m, 2H), 2.35-2.25 (m, 2H), 0.19 (s, 9H). **^{13}C NMR** ($CDCl_3$, 125 MHz, δ (ppm)) 140.0, 128.5, 126.3, 103.5, 92.3, 41.1, 36.4, 33.4, -0.2. **IR (thin film)** ν = 2934, 1707, 1461, 1379, 1266, 1143. **$[\alpha]_{25}^{D}$** = -13.5 (c = 1.0 in $CHCl_3$). **HRMS** Calcld for $C_{14}H_{19}BrSi+NH_4^+$: 312.0783, found: 312.0790.

(*S*)-(3-bromo-4-methylpent-1-yn-1-yl)trimethylsilane 4g[8]

(*R*)-1-(benzyldimethylsilyl)-4-methylpent-1-yn-3-ol (2.11 g, 12.4 mmol, 99% ee) was prepared according to general procedure **A**. It was engaged in the next step

---

[7] The ee of the bromide could not be measured and was assumed to be identical to the ee reported for the starting alcohol ee = 99%, see: Mizuta, S.; Engle, K. M.; Verhoog, S.; Galicia-López, O.; O'Duill, M.; Médebielle, M.; Wheelhouse, K.; Rassias, G.; Thompson, A. L.; Gouverneur, V. *Org. Lett.* **2013**, *15*, 1250.

[8] **4g** was obtained as an inseparable mixture with trimethyl(4-methylpent-3-en-1-yn-1-yl)silane (95:5 to 90:10) as determined by ^{1}H NMR; however, this impurity did not affect the subsequent S_N2 reaction. The ee of the bromide could not be measured and was assumed to be identical to the ee = 99% of the starting alcohol: Matsumura, K.; Hashiguchi, S.; Ikariya, T.; Noyori, R. *J. Am. Chem. Soc.* **1997**, *119*, 8738.

following general procedure **B** using triphenyl phosphine (3.90 g), bromine (762 μL) and imidazole (1.01 g). Purification by chromatography over silica gel (pentane) afforded the desired bromide **4g** (2.46 g, 85%).

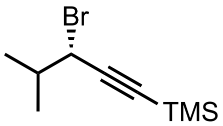

^{1}H NMR ($CDCl_3$, 400 MHz, δ (ppm)) 4.46 (d, *J* = 4.4 Hz, 1H), 2.09-1.97 (m, 1H), 1.10 (d, *J* = 6.5 Hz, 3H), 1.08 (d, *J* = 6.5 Hz, 3H), 0.17 (s, 9H). **^{13}C NMR** ($CDCl_3$, 125 MHz, δ (ppm)) 102.6, 92.7, 45.6, 35.7, 19.6, 19.2, -0.3. **Chiral GC**: Cyclodex-B 70 C, 0.5C.min^{-1} t_R = 26.6 (minor), 26.9 (major). **IR (thin film)** ν = 2923, 2892, 2860, 2834, 2144, 2123, 1446, 1366, 1292, 1233, 1152. **[α]$_{25}$^{D}** = -6.8 (c = 1.0 in $CHCl_3$). **HRMS** Calcld for $C_9H_{17}BrSi+NH_4^+$: 250.0627, found: 250.0632.

(*R*)-(3-bromo-5-((*tert*-butyldimethylsilyl)oxy)pent-1-yn-1-yl)trimethylsilane 4h[9]

(*S*)-5-((*tert*-butyldimethylsilyl)oxy)-1-(trimethylsilyl)pent-1-yn-3-ol (720 mg, 2.5 mmol , 98% ee) was prepared according to general procedure **A**. It was engaged in the next step following general procedure **B** using triphenyl phosphine (787 mg), bromine (154 μL) and imidazole (204 mg). Purification by chromatography over silica gel (pentane/diethyl ether, 95:5) afforded the desired bromide **4h** (778 mg, 89%).

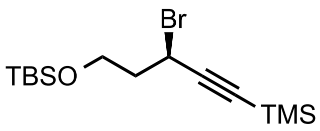

^{1}H NMR ($CDCl_3$, 500 MHz, δ (ppm)) 4.69 (t, *J* = 7.0 Hz, 1H), 3.80-3.71 (m, 2H), 2.24-2.13 (m, 2H), 0.89 (s, 9H), 0.18 (s, 9H), 0.07 (s, 6H). **^{13}C NMR** ($CDCl_3$, 125 MHz, δ (ppm)) 103.8, 91.9, 60.1, 42.4, 34.1, 25.9, 18.3, -0.3, -5.4, -5.5. **IR (thin film)** ν = 2915, 2889, 2859, 2818, 2141, 1450, 1367, 1234, 1093. **[α]$_{25}$^{D}** = -3.0 (c = 1.0 in $CHCl_3$). **HRMS** Calcld for $C_{14}H_{29}BrOSi+H^+$: 349.1018, found: 349.1031.

(*R*)-(3-bromo-7-chlorohept-1-yn-1-yl)trimethylsilane 4i

(*S*)-7-chloro-1-(trimethylsilyl)hept-1-yn-3-ol (1.75 g, 8 mmol , 99% ee) was prepared according to general procedure **A**. It was engaged in the next step following general procedure **B** using triphenyl phosphine (2.52 g), bromine (492 μL) and imidazole (654 mg). Purification by chromatography over silica gel (pentane/ether, 98:2) afforded the desired bromide **4i** (1.85 g, 82%, 98%).

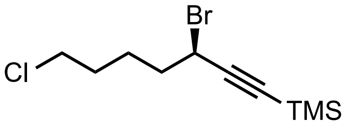

^{1}H NMR ($CDCl_3$, 400 MHz, δ (ppm)) 4.51 (t, *J* = 6.8 Hz, 1H), 3.55 (t, *J* = 6.4 Hz, 2H), 2.06-1.96 (m, 2H), 1.88-1.78 (m, 2H), 1.75-1.54 (m, 2H), 0.18 (s, 9H). **^{13}C NMR** ($CDCl_3$, 100 MHz, δ (ppm)) 103.4, 92.4, 44.5, 38.7, 36.7, 31.6,

[9] The ee of the bromide could not be measured and was assumed to be identical to the ee reported for the starting alcohol ee = 98%, see: Hartman, O.; Kalesse, M. *Org. Lett.* **2012**, *14*, 3064.

24.7, -0.3. **IR (thin film)** ν = 2917, 2860, 2828, 2140, 1436, 1293, 1233, 1158. **$[\alpha]_{25}^{D}$** = 1.9 (c = 1.0 in $CHCl_3$). **Chiral GC**: Cyclodex-B 110 C, 1C.min^{-1} t_R = 32.3 (major), 32.7 (minor). **HRMS** Calcld for $C_{20}H_{36}Cl_2Si_2+NH_4^+$:[10] 420.2076, found: 420.2082.

(*R*)-benzyl(3-bromobut-1-yn-1-yl)dimethylsilane 4j

(*S*)-4-(benzyldimethylsilyl)but-3-yn-2-ol (2.42 g, 11.1 mmol , 98% ee) was prepared according to general procedure **A**. It was engaged in the next step following general procedure **B** using triphenyl phosphine (3.49 g), bromine (682 μL) and imidazole (907 mg). Purification by chromatography over silica gel (pentane/diethyl ether, 98:2) afforded the desired bromide **4j** (2.75 g, 88%).

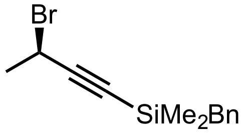

^{1}H NMR ($CDCl_3$, 400 MHz, δ (ppm)) 7.30-7.21 (m, 2H), 7.16-7.06 (m, 3H), 4.61 (q, *J* = 6.8 Hz, 1H), 2.23 (s, 2H), 1.91 (d, *J* = 6.8 Hz, 3H), 0.16 (s, 6H). **^{13}C NMR** ($CDCl_3$, 125 MHz, δ (ppm)) 138.6, 128.4, 128.2, 124.4, 106.2, 89.5, 31.0, 27.2, 25.9, -2.4. **IR (thin film)** ν = 3038, 3017, 2982, 2918, 2884, 2852, 2137, 1577, 1472, 1431, 1389, 1354, 1288, 1233, 1190, 1165. **$[\alpha]_{25}^{D}$** = -20.6 (c = 1.0 in $CHCl_3$). **HRMS** Calcld for $C_{13}H_{17}BrSi+NH_4^+$: 298.0627, found: 298.0624.

(*S*)-(3-bromobut-1-yn-1-yl)benzene 4k[11]

(*R*)-4-phenylbut-3-yn-2-ol (3.20 g, 21.9 mmol, 97% ee) was prepared according to general procedure **A**. It was engaged in the next step following general procedure **B** using triphenyl phosphine (6.89 g), bromine (1.35 mL) and imidazole (1.79 g). Purification by chromatography over silica gel (pentane/diethyl ether, 98:2) afforded the desired bromide **4k** (3.62 g, 79%).

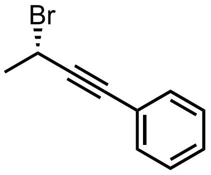

^{1}H NMR ($CDCl_3$, 500 MHz, δ (ppm)) 7.46-7.41 (m, 2H), 7.36-7.28 (m, 3H), 4.87 (q, *J* = 7.0 Hz, 1H), 2.0 (d, *J* = 7.0 Hz, 3H). **^{13}C NMR** ($CDCl_3$, 125 MHz, δ (ppm)) 131.8, 128.8, 128.3, 122.1, 89.1, 86.0, 32.0, 27.5. **IR (thin film)** ν = 3037, 3014, 2991, 2952, 2935, 2884, 2821, 2204, 2178, 1574, 1469, 1421, 1355, 1300, 1164, 1051. **$[\alpha]_{25}^{D}$** = -14.8 (c = 1.0 in $CHCl_3$). **HRMS** Calcld for $C_{10}H_9Br^+$: 207.9888, found: 207. 9895.

---

[10] The observed mass corresponds to the dimer resulting from recombination of the corresponding radical:

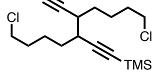

[11] The ee of the bromide could not be measured and was assumed to be identical to the ee reported for the starting alcohol ee = 97%, see: Newcomb, E. T.; Ferreira, M. *Org. Lett.* **2013**, *15*, 1772.

(*R*)-(4-(((*tert*-butyldimethylsilyl)oxy)methyl)-2-(1-(trimethylsilyl)hex-1-yn-3-yl)furan-3-yl)methanol 2a

Product **2a** was prepared according to general procedure **C**[12] using (4-(((*tert*-butyldimethylsilyl)oxy)methyl)furan-3-yl)methanol **1a**[13] (485 mg, 2 mmol), CuCN (89 mg, 1 mmol) and (*S*)-(3-bromohex-1-yn-1-yl)trimethylsilane **4a** (233 mg, 1 mmol). After purification by flash chromatography over silica gel (petroleum ether/ethyl acetate, 95:5 to 90:10), **2a** was obtained (304 mg, 77%, 96% ee).

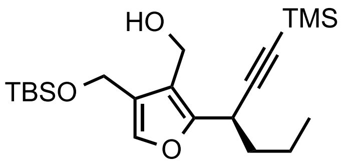

^{1}H NMR ($CDCl_3$, 400 MHz, δ (ppm)) 7.20 (s, 1H), 4.59 (s, 2H), 4.55 (d, *J* = 2.8 Hz), 3.78 (t, *J* = 7.2 Hz, 1H), 3.13 (brs, 1H), 1.85-1.71 (m, 1H), 1.46-1.30 (m, 1H), 0.89 (s, 9H), 0.14 (s, 9H), 0.10 (s, 6H). **^{13}C NMR** ($CDCl_3$, 100 MHz, δ (ppm)) 150.7, 137.8, 125.0, 119.6, 105.3, 86.4, 56.6, 54.6, 37.4, 30.8, 25.8, 20.3, 18.2, 13.5, 0.0, -5.4. **IR (thin film)** ν = 3419, 2916, 2890, 2819, 2143, 1544, 1445, 1390, 1233, 1033. **$[\alpha]_{25}^{D}$** = -19.2 (c = 1.0 in $CHCl_3$). **Chiral HPLC**: Chiralpak® IA, 0.1% isopropanol in heptane, 0.8 mL.min^{-1}, λ = 220 nm, t_R = 14.40 (minor), 19.45 (major). **HRMS** Calcld for $C_{21}H_{38}O_3Si_2+Na^+$: 417.2257, found: 417.2254.

(R)-(2-(1-(4-bromophenyl)-5-methylhex-1-yn-3-yl)-4-(((*tert*butyldimethylsilyl)-oxy)methyl)furan-3-yl)methanol 2b

Product **2b** was prepared according to general procedure **C**[12] using (4-(((*tert*-butyldimethylsilyl)oxy)methyl)furan-3-yl)methanol **1a** (485 mg, 2 mmol), CuCN (89 mg, 1 mmol) and (S)-1-bromo-4-(3-bromo-5-methylhex-1-yn-1-yl)benzene **4d** (330 mg, 1 mmol). After purification by flash chromatography over silica gel (petroleum ether/ethyl acetate, 95:5 to 90:10), **2b** was obtained (310 mg, 63%, 97% ee).

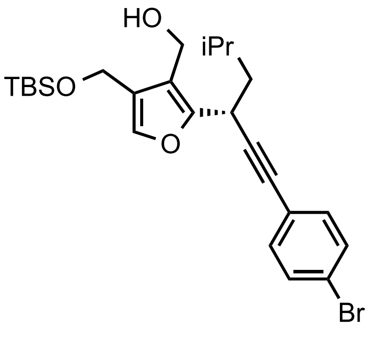

^{1}H NMR ($CDCl_3$, 400 MHz, δ (ppm)) 7.43-7.38 (m, 2H), 7.30-7.26 (m, 2H), 4.62 (s, 2H), 4.60 (s, 2H), 4.05 (t, *J* = 7.6 Hz, 1H), 3.20 (brs, 1H), 1.93-1.82 (m, 1H), 1.78-1.66 (m, 2H), 0.97 (d, *J* = 7.7 Hz, 3H), 0.95 (d, *J* = 7.7 Hz, 3H), 0.93 (s, 9H), 0.14 (s, 3H), 0.13 (s, 3H). **^{13}C NMR** ($CDCl_3$, 100 MHz, δ (ppm)) 150.8, 138.0, 33.1, 131.6, 125.1, 122.8, 122.0, 119.6, 104.9, 90.0, 81.0, 56.7, 54.7, 44.0, 28.6, 25.8, 22.5, 21.9, 18.3, -5.4. **IR (thin film)** ν = 3400, 3011, 2913, 2889, 2872, 1544, 1465, 1449, 1371, 1238, 1055. **$[\alpha]_{25}^{D}$** = 35.5 (c = 1.0 in $CHCl_3$). **Chiral**

---

[12] Except that 4.4 mmol of *n*BuLi was used instead of 2.2 mmol.

[13] **1a** was prepared from 4-(hydroxymethyl)furan-3-carbaldehyde by TBS protection followed $NaBH_{4(s)}$ reduction, see: Clark, J. S.; Marlin, F.; Nay, B.; Wilson, C. *Org. Lett.* **2003**, *5*, 89.

HPLC: Chiralpak® IC, 0.5% isopropanol in heptane, 0.8 mL.min^{-1}, λ = 254 nm, t_R = 10.5 (major), 11.8 (minor). **HRMS** Calcld for $C_{25}H_{35}BrO_3Si+Na^+$: 513.1437, found: 513.1425.

(*R*)-2-(1-(trimethylsilyl)hex-1-yn-3-yl)furan-3-carboxylic acid 2c

Product **2c** was prepared according to general procedure **C**[12] using 3-furoic acid (224 mg, 2 mmol), CuCN (89 mg, 1 mmol) and (*S*)-(3-bromohex-1-yn-1-yl)trimethylsilane **4a** (233 mg, 1 mmol). After purification by flash chromatography over silica gel (petroleum ether/ethyl acetate/acetic acid, 78:20:2), **2c** was obtained (188 mg, 71%, 95% ee).

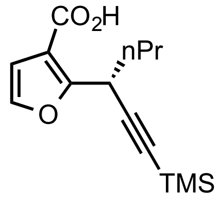

^{1}H NMR ($CDCl_3$, 400 MHz, δ (ppm)) 7.35 (d, *J* = 2.0 Hz, 1H), 6.71 (d, *J* = 2.0 Hz, 1H), 4.67 (dd, *J* = 7.2 Hz, *J* = 7.2 Hz, 1H), 1.95-1.73 (m, H), 1.52-1.31 (m, 2H), 0.93 (t, *J* = 7.6 Hz, 3H), 0.16 (s, 9H). **^{13}C NMR** ($CDCl_3$, 100 MHz, δ (ppm)) 169.3, 161.2, 141.5, 12.6, 110.8, 103.9, 86.5, 37.0, 30.9, 20.3, 13.6, 0.0. **IR (thin film)** ν = 3300, 2919, 2834, 2146, 1663, 1575, 1498, 1435, 1301, 1233, 1187. **$[\alpha]_{25}^D$** = 118.3 (c = 1.0 in $CHCl_3$). **Chiral HPLC**: Chiralpak® IB, 10% isopropanol in heptane, 0.8 mL.min^{-1}, λ = 254 nm, t_R = 8.81 (major), 10.65 (minor). **HRMS** Calcld for $C_{14}H_{20}O_3Si+Na^+$: 287.1080, found: 287.1073.

(*S*)-trimethyl(3-(5-(2-methyl-1,3-dioxolan-2-yl)furan-2-yl)oct-7-en-1-yn-1-yl)silane 2d

Product **2d** was prepared according to general procedure **C** using 2-(furan-2-yl)-2-methyl-1,3-dioxolane[14] (308 mg, 2 mmol), CuCN (89 mg, 1 mmol) and (*R*)-(3-bromohept-6-en-1-yn-1-yl)trimethylsilane **4e** (245 mg, 1 mmol). After purification by flash chromatography over silica gel (petroleum ether/diethyl ether 95:5), **2d** was obtained (276 mg, 83%, 98% ee).

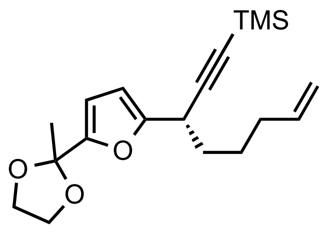

^{1}H NMR ($CDCl_3$, 400 MHz, δ (ppm)) 6.22 (d, *J* = 3.2 Hz, 1H), 6.14 (dd, *J* = 3.2 Hz, *J* = 1.2 Hz, 1H), 5.86-5.76 (m, 1H), 5.05 (dd, *J* = 17.2 Hz, *J* = 2.0 Hz, 1H), 4.98 (dd, *J* = 10.4 Hz, *J* = 1.2 Hz, 1H), 4.06-3.95 (m, 4H), 3.76 (dd, *J* = 8.4 Hz, *J* = 5.6 Hz, 1H), 2.25-2.15 (m, 2H), 1.98-1.77 (m, 2H), 1.71 (s, 3H), 0.17 (s, 9H). **^{13}C NMR** ($CDCl_3$, 100 MHz, δ (ppm)) 154.1, 153.3, 137.6, 115.2, 107.1, 106.1, 104.9, 104.6, 87.1, 65.1, 65.0, 33.7, 31.9, 30.9, 24.2, 0.0. **IR (thin film)** ν = 3035, 2916, 2854, 2145, 1617, 1576, 1530, 1421, 1356, 1233, 1177, 1094. **$[\alpha]_{25}^D$** = 19.5 (c = 1.0 in $CHCl_3$). **Chiral HPLC**: Chiralpak® IC, heptane, 0.8 mL.min^{-1}, λ = 220 nm, t_R = 4.42 (major), t_R = 5.03 (minor). **HRMS** Calcld for $C_{19}H_{28}O_3Si+Na^+$: 341.1549, found: 341.1536.

---

[14] Dang, Q. et al. *J. Med. Chem.* **2011**, *54*, 153.

(*S*)-(3-(benzofuran-2-yl)-5-phenylpent-1-yn-1-yl)trimethylsilane 2e

Product **2e** was prepared according to general procedure **C** using benzofuran (236 mg, 2 mmol), CuCN (89 mg, 1 mmol) and (*R*)-(3-bromo-5-phenylpent-1-yn-1-yl)trimethylsilane **4f** (295 mg, 1 mmol). After purification by flash chromatography over silica gel (petroleum ether/diethyl ether 98:2), **2e** was obtained (229 mg, 69%, 94% ee).

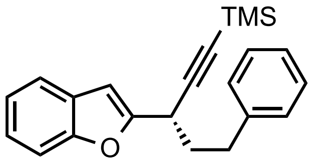

^{1}H NMR ($CDCl_3$, 500 MHz, δ (ppm)) 7.54 (dd, *J* = 7.5 Hz, *J* = 1.5 Hz, 1H), 7.45 (d, *J* = 7.5 Hz, 1H), 7.34-7.28 (m, 2H), 7.27-7.18 (m, 5H), 6.66 (s, 1H), 3.92 (dd, *J* = 8.0 Hz, *J* = 5.3 Hz, 1H), 2.94-2.77 (m, 2H), 2.34-2.26 (m, 1H), 2.24-2.14 (m, 1H), 0.25 (s, 9H). **^{13}C NMR** ($CDCl_3$, 125 MHz, δ (ppm)) 157.1, 154.9, 141.2, 128.5, 128.4, 126.0, 123.6, 122.6, 120.6, 111.0, 104.3, 103.1, 88.0, 35.8, 32.9, 32.3, 0.1. **IR (thin film)** ν = 3024, 3020, 2985, 2915, 2859, 2146, 1578, 1563, 1433, 1234. **$[\alpha]_{25}^{D}$** = 18.7 (c = 1.0 in $CHCl_3$). **Chiral HPLC[15]**: Chiralpak® IA, heptane, 1.0 mL.min^{-1}, λ = 254 nm, t_R = 7.70 (major), t_R = 9.77 (minor). **HRMS** Calcld for $C_{22}H_{24}OSi+Na^+$: 355.1494, found: 355.1489.

(*S*)-(3-(4,5-dihydrofuran-2-yl)-5-phenylpent-1-yn-1-yl)trimethylsilane 2f

Product **2f** was prepared according to general procedure **C** using dihydrofuran (140 mg, 2 mmol), CuCN (89 mg, 1 mmol) and (*R*)-(3-bromo-5-phenylpent-1-yn-1-yl)trimethylsilane **4f** (295 mg, 1 mmol). After purification by flash chromatography over silica gel (petroleum ether/diethyl ether/tirethylamine 97:2:1), **2f** was obtained (182 mg, 64%, 91% ee).

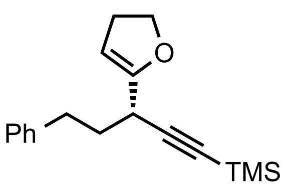

^{1}H NMR ($CDCl_3$, 400 MHz, δ (ppm)) 7.32-7.26 (m, 2H), 7.24-7.14 (m, 3H), 4.91 (brs, 1H), 4.36 (t, *J* = 9.2 Hz, 1H), 3.15-3.17 (m, 1H), 2.86-2.69 (m, 2H), 2.67-2.59 (m, 2H), 2.09-1.98 (m, 1H), 1.96-1.84 (m, 1H), 0.21 (s, 9H). **^{13}C NMR** ($CDCl_3$, 125 MHz, δ (ppm)) 157.0, 141.7, 128.5, 128.3, 125.8, 105.5, 95.7, 87.0, 70.6, 4.5, 32.9, 31.8, 29.8, -0.1. **IR (thin film)** ν = 3019, 2916, 2855, 2821, 2146, 1641, 1475, 1434, 1231, 1135.). **$[\alpha]_{25}^{D}$** = 20.9 (c = 1.0 in $CHCl_3$). **Chiral HPLC**: Chiralpak® IA, 1% isopropanol in heptane, 0.8 mL.min^{-1}, λ = 220 nm, t_R = 4.6 (major), 4.8 (minor). **HRMS** Calcld for $C_{18}H_{24}OSi+Na^+$: 307.1494, found: 307.1489.

(*R*)-trimethyl(4-methyl-3-(thiophen-2-yl)pent-1-yn-1-yl)silane 2g

Product **2g** was prepared according to general procedure **C** using thiophene **1c** (168 mg, 2 mmol), CuCN (89 mg, 1 mmol) and (*S*)-(3-bromo-4-methylpent-1-yn-1-yl)trimethylsilane **4g** (233 mg, 1 mmol). After purification by flash chromatography

[15] Chiral HPLC analysis was ran on the corresponding terminal alkyne which was obtained by deprotection of the TMS protecting group (TBAF 3 equiv., AcOH 30 mol%, THF, rt)

over silica gel (petroleum ether/diethyl ether 99:1), **2g** was obtained (165 mg, 70%, 94% ee).

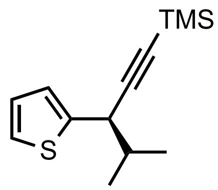

^{1}H NMR ($CDCl_3$, 400 MHz, δ (ppm)) 7.19-7.15 (m, 1H), 6.99-6.92 (m, 2H), 3.84 (d, *J* = 5.2 Hz, 1H), 2.09-1.97 (m, 1H), 1.05 (dd, *J* = 6.8 Hz, *J* = 1.6 Hz, 3H), 0.96 (dd, *J* = 6.8 Hz, *J* = 1.6 Hz, 3H), 0.21 (s, 9H). **^{13}C NMR** ($CDCl_3$, 125 MHz, δ (ppm)) 144.3, 126.4, 125.0, 123.7, 105.7, 88.2, 41.2, 35.3, 20.8, 18.2, 0.1. **IR (thin film)** ν = 2919, 2889, 2857, 2831, 2142, 1443, 1232. **[α]$_{25}$^{D}** = -8.1 (c = 1.0 in $CHCl_3$). **Chiral HPLC**: Chiralpak® IA, heptane, 0.8 mL.min^{-1}, λ = 220 nm, t_R = 4.88 (major), 5.29 (minor). **HRMS** Calcld for $C_{13}H_{20}SSi+NH_4^+$: 254.1399, found: 254.1407.

(*S*)-(3-(benzo[*b*]thiophen-2-yl)-5-((*tert*-butyldimethylsilyl)oxy)pent-1-yn-1-yl)trimethylsilane 2h

Product **2h** was prepared according to general procedure **C** using benzothiophene (268 mg, 2 mmol), CuCN (89 mg, 1 mmol) and (*R*)-(3-bromo-5-((*tert*-butyldimethylsilyl)oxy)pent-1-yn-1-yl)trimethylsilane **4h** (349 mg, 1 mmol). After purification by flash chromatography over silica gel (petroleum ether/diethyl ether 98:2), **2h** was obtained (248 mg, 71%, 98% ee).

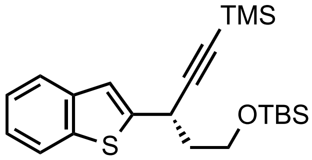

^{1}H NMR ($CDCl_3$, 500 MHz, δ (ppm) 7.80-7.76 (m, 1H), 7.71-7.67 (m, 1H), 7.34-7.30 (m, 1H), 7.29-7.25 (m, 1H), 7.20 (s, 1H), 4.22 (dd, *J* = 9.0 Hz, *J* = 5.5 Hz, 1H), 3.89-3.83 (m, 1H), 3.77-3.71 (m, 1H), 2.16-2.08 (m, 1H), 2.06-1.99 (m, 1H), 0.92 (s, 9H), 0.20 (s, 9H), 0.1 (s, 3H), 0.1 (s, 3H). **^{13}C NMR** ($CDCl_3$, 125 MHz, δ (ppm)) 146.0, 139.7, 139.4, 124.1, 123.8, 123.1, 122.2, 121.0, 106.2, 87.8, 60.2, 40.8, 30.9, 26.0, 18.4, 0.0, -5.3, -5.4. **IR (thin film)** ν = 3017, 2987, 2914, 2857, 2142, 1899, 1440, 1233, 1087. **[α]$_{25}$^{D}** = 16.6 (c = 1.0 in $CHCl_3$). **Chiral HPLC**:[16] Chiralpak® IC, 2% isopropanol in heptane, 0.6 mL.min^{-1}, λ = 254 nm, t_R = 16.27 (major), 16.99 (minor). **HRMS** Calcld for $C_{22}H_{34}OSSi_2+H^+$: 403.1947, found: 403.1943.

(*S*)-(7-chloro-3-(2,3-dihydrothieno[3,4-*b*][1,4]dioxin-5-yl)hept-1-yn-1-yl)trimethylsilane 2i

Product **2i** was prepared according to general procedure **C** using 2,3-dihydrothieno[3,4-*b*][1,4]dioxine (284 mg, 2 mmol), CuCN (89 mg, 1 mmol) and (*R*)-(3-bromo-7-chlorohept-1-yn-1-yl)trimethylsilane **4i** (282 mg, 1 mmol). After purification by flash chromatography over silica gel (petroleum ether/diethyl ether 95:5 to 90:10), **2i** was obtained (322 mg, 94%, 97% ee).

^{1}H NMR ($CDCl_3$, 400 MHz, δ (ppm)) 6.18 (s, 1H), 4.17 (s, 4H), 3.96 (dd, *J* = 7.2 Hz, *J* = 7.2 Hz, 1H), 3.53 (t, *J* = 6.8 Hz, 2H), 1.85-1.72 (m, 4H), 1.68-1.45 (m, 2H), 0.17 (s, 9H).

[16] Chiral HPLC analysis was ran on the corresponding alcohol which was obtained by deprotection of the TBS protecting group (2N $HCl_{(aq.)}$ in MeOH, 5 min.)

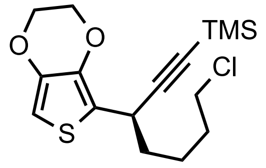

^{13}C NMR ($CDCl_3$, 125 MHz, δ (ppm)) 141.2, 137.3, 117.9, 106.5, 106.5, 96.5, 86.9, 64.7, 64.6, 44.9, 36.0, 32.1, 30.1, 24.3, 0.0. **IR (thin film)** ν = 3071, 2915, 2830, 2141, 1480, 1419, 1347, 1231, 1149. **[α]$_{25}$^{D}** = 1.2 (c = 1.0 in $CHCl_3$). **Chiral HPLC**: Chiralpak® IC, heptane, 0.8 mL.min^{-1}, λ = 254 nm, t_R = 37.9 (minor), 38.9 (major). **HRMS** Calcld for $C_{16}H_{23}ClO_2SSi+H^+$: 343.0955, found: 343.0943.

(*S*)-benzyl(3-(3-((methoxymethoxy)methyl)benzo[*b*]thiophen-2-yl)but-1-yn-1-yl)dimethylsilane 2j

Product **2j** was prepared according to general procedure **C** using 3-((methoxymethoxy)methyl)benzo[*b*]thiophene[17] (416 mg, 2 mmol), CuCN (89 mg, 1 mmol) and (*R*)-benzyl(3-bromobut-1-yn-1-yl)dimethylsilane **4j** (281 mg, 1 mmol). After purification by flash chromatography over silica gel (petroleum ether/diethyl ether 98:2 to 90:10), **2j** was obtained (339 mg, 87%, 93% ee).

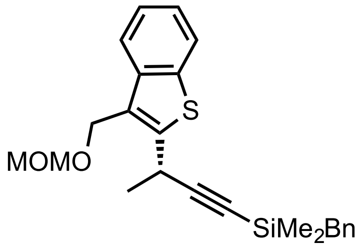

^{1}H NMR ($CDCl_3$, 500 MHz, δ (ppm)) 7.83-7.80 (m, 2H), 7.41-7.36 (m, 1H), 7,35-7.30 (m, 1H), 7.20-7.15 (m, 2H), 7.09-7.05 (m, 3H), 4.83 (d, *J* = 12.0 Hz, 1H), 4.78 (d, *J* = 12.0 Hz, 1H), 4.69 (d, *J* = 7.0 Hz, 1H), 4.68 (d, *J* = 7.0 Hz, 1H), 4.37 (q, *J* = 7.5 Hz, 1H), 3.44 (s, 3H), 2.21 (s, 2H), 1.62 (d, *J* = 7.5 Hz, 3H), 0.13 (s, 3H0, 0.13 (s, 3H). **^{13}C NMR** ($CDCl_3$, 125 MHz, δ (ppm)) 146.8, 139.8, 139.0, 138.2, 128.4, 128.1, 126.6, 124.3, 124.2, 122.3, 121.8, 109.2, 95.2, 84.9, 59.7, 55.5, 26.9, 26.2, 24.5, -2.1. **IR (thin film)** ν =3036, 3017, 2982, 2918, 2782, 2736, 2138, 1912, 1577, 1472, 1431, 1416, 1384, 1353, 1273. **[α]$_{25}$^{D}** = -23.4 (c = 1.0 in $CHCl_3$). **Chiral HPLC**: Chiralpak® IB, 0.1% isopropanol in heptane, 0.8 mL.min^{-1}, λ = 254 nm, t_R = 20.1 min (major), 22.3 (minor). **HRMS** Calcld for $C_{24}H_{28}O_2SSi+NH_4^+$: 426.1923, found: 426.1918.

(*S*)-1-methyl-2-(1-(trimethylsilyl)oct-7-en-1-yn-3-yl)-1*H*-indole 2k

Product **2k** was prepared according to general procedure **C** using *N*-methylindole (262 mg, 2 mmol), CuCN (89 mg, 1 mmol) and (*R*)-(3-bromohept-6-en-1-yn-1-yl)trimethylsilane **4e** (245 mg, 1 mmol). After purification by flash chromatography over silica gel (petroleum ether/DCM 95:5 to 80:20), **2k** was obtained (251 mg, 81%, 95% ee).

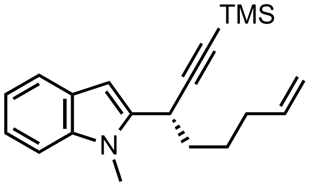

^{1}H NMR ($CDCl_3$, 400 MHz, δ (ppm) 7.62 (d, *J* = 7.6 Hz, 1H), 7.33 (d, *J* = 8.4 Hz, 1H), 7.26-7.22 (m, 1H), 7.16-7.11 (m, 1H), 6.44 (s, 1H), 5.96-5.85 (m, 1H), 5.19 (d, *J* = 18.8 Hz, 1H), 5.13 (d, *J* = 10.0 Hz, 1H), 3.96 (t, *J* = 7.4 Hz, 2H), 3.85 (s,

[17] 3-((methoxymethoxy)methyl)benzo[*b*]thiophene was obtained by reacting benzo-[*b*]thiophen-3-ylmethanol with MOMCl and Hünig's base in DCM.

3H), 2.47-2.32 (m, 2H), 2.16-2.02 (m, 2H), 0.26 (s, 9H). **^{13}C NMR** ($CDCl_3$, 100 MHz, δ (ppm)) 139.2, 137.7, 137.5, 127.5, 121.1, 120.2, 119.4, 115.6, 108.8, 105.7, 99.3, 87.3, 33.7, 31.3, 30.3, 30.0, 0.0. **IR (thin film)** ν = 3013, 2915, 2140, 1617, 1519, 1448, 1414, 1379, 1320, 1298, 1232. **$[\alpha]_{25}{}^{D}$** = 1.5 (c = 1.0 in $CHCl_3$). **Chiral HPLC**: Chiralpak® **HRMS** Calcld for $C_{19}H_{25}NSi+H^+$: 296.1829, found: 296.1835.

(*S*)-4-chloro-2-(7-chloro-1-(trimethylsilyl)hept-1-yn-3-yl)-1-methyl-1*H*-indole 2l

Product **2l** was prepared according to general procedure **C** using 4-chloro-1-methyl-1*H*-indole (331 mg, 2 mmol), CuCN (89 mg, 1 mmol) and (*R*)-(3-bromo-7-chlorohept-1-yn-1-yl)trimethylsilane **4i** (282 mg, 1 mmol). After purification by flash chromatography over silica gel (petroleum ether/DCM 95:5 to 80:20), **2l** was obtained (202 mg, 55%, 97% ee).

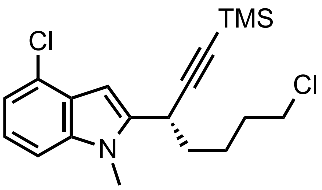

^{1}H NMR ($CDCl_3$, 500 MHz, δ (ppm)) 7.24-7.15 (m, 1H), 7.13-7.05 (m, 2H), 6.47 (s, 1H), 3.85 (t, *J* = 7.2 Hz, 1H), 3.79 (s, 3H), 3.58 (t, *J* = 6.8 Hz, 2H), 2.04-1.82 (m, 4H), 1.82-1.60 (m, 2H), 0.16 (s, 9H). **^{13}C NMR** ($CDCl_3$, 100 MHz, δ (ppm)) 139.9, 138.5, 126.2, 125.6, 121.7, 119.2, 107.6, 105.0, 97.9, 44.7, 33.6, 32.1, 31.0, 30.5, 24.7, 0.0. **$[\alpha]_{25}{}^{D}$** = 27.0 (c = 1.0 in $CHCl_3$). **Chiral HPLC**: Chiralpak® IA, 0.1% isopropanol in heptane, 0.8 mL.min^{-1}, λ = 254 nm, t_R = 8.9 (major), 9.6 (minor). **HRMS** Calcld for $C_{19}H_{25}Cl_2NSi+H^+$: 366.1212, found: 366.1203.

(*R*)-5-(4-phenylbut-3-yn-2-yl)-2,3-dihydrothieno[3,4-*b*][1,4]dioxine 2m

Product **2m** was prepared according to general procedure **C** using 2,3-dihydrothieno[3,4-*b*][1,4]dioxine (284 mg, 2 mmol), CuCN (89 mg, 1 mmol) and (*S*)-(3-bromobut-1-yn-1-yl)benzene **4k** (209 mg, 1 mmol). After purification by flash chromatography over silica gel (petroleum ether/diethyl ether 95:5 to 90:10), **2m** was obtained (197 mg, 73%, 80% ee).

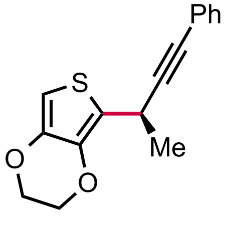

^{1}H NMR ($CDCl_3$, 400 MHz, δ (ppm)) 7.48-7.41 (m, 2H), 7.32-7.27 (m, 3H), 6.19 (s, 1H), 4.26 (q, *J* = 6.8 Hz, 1H), 4.24-4.17 (m, 4H), 1.59 (d, *J* = 6.8 Hz, 3H). **^{13}C NMR** ($CDCl_3$, 75 MHz, δ (ppm)) 141.4, 137.0, 131.6, 128.1, 127.8, 123.4, 119.6, 96.1, 91.4, 81.5, 64.7, 64.6, 24.5, 22.9. **IR (thin film)** ν = 3112, 2978, 2928, 2871, 2362, 2340, 1597, 1499, 1439, 1365, 1166, 1133, 1082, 1065. **$[\alpha]_{25}{}^{D}$** =-50.7 (1M in $CHCl_3$). **Chiral HPLC**: Chiralpak® IC, 0.1% isopropanol in heptane, 0.8 mL.min^{-1}, λ = 254 nm, t_R = 35.6 min (minor), 44.4 min (major). **HRMS** Calcld for $C_{16}H_{14}SO_2+H^+$: 271.0788, found: 271.0793.

(*R*)-1-(2-(4-(trimethylsilyl)but-3-yn-2-yl)furan-3-yl)ethan-1-one 5a

Product **2n** was prepared according to general procedure **C** using 3-furan carboxaldehyde **1b** (192 mg, 2 mmol), CuCN (89 mg, 1 mmol) and (*S*)-(3-bromobut-1-yn-1-yl)trimethylsilane **4b** (205 mg, 1 mmol). After purification by flash chromatography over silica gel (petroleum ether/diethyl ether 95:5 to 85:15), **2n** was obtained (199 mg, 84%).
Alcohol **2n** was dissolved in DCM (10 mL) and $NaHCO_3$ (141 mg, 2 equiv.) followed by Dess-Martin periodinane (534 mg, 1.5 equiv.) were added. After 3 hours the TLC showed no remaining alcohol **2n** and the residue was filtrated over silica, evaporated to dryness and purified by chromatography over silica gel (petroleum ether/diethyl ether 95:5 to 85:15) to deliver **5a** (159 mg, 81%, 95% ee).

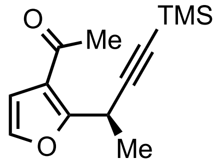

^{1}H NMR ($CDCl_3$, 400 MHz, δ (ppm)) 7.31 (d, *J* = 2.0 Hz, 1H), 6.60 (d, *J* = 2.0 Hz, 1H), 4.73 (q, *J* = 7.2 Hz, 1H), 2.40 (s, 3H), 1.47 (d, *J* = 7.2 Hz, 3H), 0.13 (s, 9H). **^{13}C NMR** ($CDCl_3$, 100 MHz, δ (ppm)) 193.9, 159.3, 141.0, 119.7, 110.4, 110.4, 105.2, 85.5, 29.2, 25.8, 20.4, 0.0. I**R (thin film)** ν = 3102, 3076, 2944, 2917, 2896, 2858, 2135, 1658, 1558, 1492, 1426, 1401, 1379, 1340, 1293, 1273, 1231. **$[\alpha]_{25}^{D}$** = 94.5. **Chiral HPLC**: Chiralpak® IB, 0.1% isopropanol in heptane, 0.6 mL.min^{-1}, λ = 254 nm, t_R = 20.5 (major), 22.7 (minor). **HRMS** Calcld for $C_{13}H_{18}O_2Si+H^+$: 235.1154, found: 235.1145.

(2*S*,5*R*)-2-methyl-5-(thiophen-2-yl)-7-(trimethylsilyl)hept-6-yn-1-ylbenzene sulfonate 2o

Product **2o** was prepared according to general procedure **C** using thiophene **1c** (402 mg, 3 mmol), CuCN (134 mg, 1 mmol) and (3*S*,6*S*)-6-bromo-3-methyl-8-(trimethylsilyl)oct-7-yn-1-yl benzenesulfonate **4c** (647 mg, 1.5 mmol). After purification by flash chromatography over silica gel (petroleum ether/ethyl acetate 95:5 to 85:15), **2o** was obtained (535 mg, 82%, dr > 95:5).

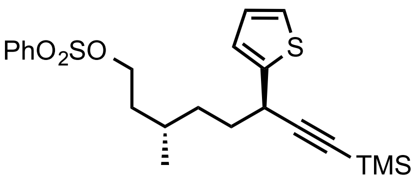

^{1}H NMR ($CDCl_3$, 500 MHz, δ (ppm)) 7.93-7.89 (m, 2H), 7.67-7.62 (m, 1H), 7.58-7.53 (m, 2H), 7.17 (dd, *J* = 5 Hz, *J* = 1.5 Hz, 1H), 6.95-6.91 (m, 2H), 4.12-4.04 (m, 2H), 3.85 (dd, *J* = 6 Hz, *J* = 8.3 Hz, 1H), 1.80-1.64 (m, 3H), 1.60-1.50 (m, 1H), 1.48-1.24 (m, 4H), 0.81 (d, *J* = 6.5 Hz, 3H), 0.18 (s, 9H). **^{13}C NMR** ($CDCl_3$, 125 MHz, δ (ppm)) 145.1, 136.1, 133.6, 129.2, 127.8, 126.5, 124.3, 123.8, 107.0, 87.4, 69.1, 35.7, 35.6, 33.9, 33.6, 28.8, 18.9, 0.0. **$[\alpha]_{25}^{D}$** = -14.0 (c = 1.0 in $CHCl_3$). **MS** Calcld for $C_{22}H_{30}O_3S_2Si+Na^+$: 457.1, found: 457.1.

(3*S*,6*R*)-3-methyl-6-(thiophen-2-yl)-8-(trimethylsilyl)oct-7-yn-1-ylbenzene sulfonate 5b

Sulfonate **2o** (217 mg, 0.5 mmol) was dissolved in dry DMF (3 mL) and NaN_3 (98 mg, 3 equiv.) was added. The resulting mixture was heated to 90°C for 1.5 hours. After work up (Et_2O, H_2O), the residue was purified by flash chromatography over silica gel (petroleum ether/diethyl ether 95:5) to deliver **5b** (142 mg, 89%, dr > 95:5).

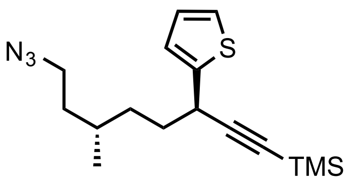

^{1}H NMR ($CDCl_3$, 400 MHz, δ (ppm)) 7.20-7.15 (m, 1H), 7.00-6.91 (m, 2H), 3.93 (t, *J* = 7.2 Hz, 1H), 3.36-3.19 (m, 2H), 1.90-1.77 (m, 4H), 1.69-1.28 (m, 3H), 0.92 (d, *J* = 6.5 Hz, 3H), 0.20 (s, 9H). **^{13}C NMR** ($CDCl_3$, 100 MHz, δ (ppm)) 145.2, 126.5, 124.3, 123.8, 107.1, 87.4, 49.4, 35.7, 35.6, 33.9, 33.6, 29.9, 19.2, 0.0. **IR (thin film)** ν =2917, 2832, 2225, 2141, 1440, 1360, 1232, 1026. **$[\alpha]_{22}^{D}$** = -19.4 (c = 1.0 in $CHCl_3$). **HRMS** Calcld for $C_{16}H_{25}N_3SSi+H^+$: 320.1616, found: 320.1599.

(*R*)-(3-(5-methoxybenzofuran-2-yl)but-1-yn-1-yl)trimethylsilane 2p

Product **2p** was prepared according to general procedure **C** using 5-methoxybenzofuran **1d** (889 mg, 6 mmol), CuCN (267 mg, 3 mmol) and (*S*)-(3-bromobut-1-yn-1-yl)trimethylsilane **4b** (616 mg, 3 mmol). After purification by flash chromatography over silica gel (petroleum ether/diethyl 99:1 to 97:3), **2p** was obtained (646 mg, 79%, 96% ee).

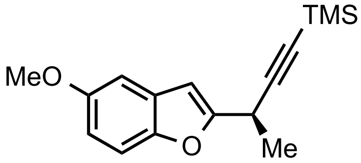

^{1}H NMR ($CDCl_3$, 400 MHz, δ (ppm)) 7.36-7.31 (m, 1H), 7.0 (d, *J* = 2.6 Hz, 1H), 6.85 (dd, *J* = 8.8 Hz, *J* = 2.6 Hz, 1H), 6.58-6.55 (m, 1H), 3.97 (qd, *J* = 8.4 Hz, *J* = 1.2 Hz, 1H), 3.84 (s, 3H), 1.61 (d, *J* = 8.4 Hz, 3H), 0.22 (s, 9H). **^{13}C NMR** ($CDCl_3$, 100 MHz, δ (ppm)) 159.2, 155.8, 149.9, 129.0, 112.0, 111.3, 105.8, 103.4, 102.2, 86.2, 55.8, 27.3, 20.4, 0.0. **IR (thin film)** ν =3005, 2944, 2916, 2858, 2792, 2141, 1595, 1579, 1445, 1429, 1232, 1210. **$[\alpha]_{25}^{D}$** = -0.6 (c = 1.0 in $CHCl_3$). **Chiral HPLC**: Chiralpak® IC, 0.5% isopropanol in heptane, 0.8 mL.min^{-1}, λ = 254 nm, t_R = 8.1 min (minor), 9.1 min (major). **HRMS** Calcld for $C_{16}H_{20}O_2Si^+$: 272.1233, found: 272.1236.

(*R*)-2-(but-3-yn-2-yl)-5-methoxybenzofuran

TMS-alkyne **2p** (409 mg, 1.5 mmol) was dissolved in THF (5 mL) and AcOH (58 μL, 1 mmol) followed by TBAF (1M in THF, 1.8 mL, 1.8 mmol). When the TLC showed full conversion, the reaction medium was quenched with water and worked-up. The residue was evaporated to dryness and purified by flash chromatography over silica gel (petroleum ether/diethyl 98:2 to 96:4) to deliver the terminal alkyne (288 mg, 96%).

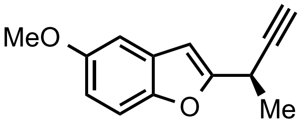

^{1}H NMR ($CDCl_3$, 400 MHz, δ (ppm)) 7.36-7.31 (m, 1H), 7.0 (d, *J* = 2.8 Hz, 1H), 6.85 (dd, *J* = 8.8 Hz, *J* = 2.8 Hz, 1H), 6.58-6.55 (m, 1H), 3.99-3.90 (m, 1H), 3.84 (s, 3H), 2.29 (dq, *J* =

2.8 Hz, *J* = 0.4 Hz, 1H), 1.63 (d, *J* = 6.8 Hz, 3H). **^{13}C NMR** ($CDCl_3$, 100 MHz, δ (ppm)) 158.7, 155.8, 149.9, 128.9, 112.2, 111.4, 103.4, 102.2, 83.8, 70.0, 55.9, 26.1, 20.2. **IR (thin film)** ν = 3248, 2947, 2897, 2793, 1593, 1580, 1455, 1429, 1270, 1189, 1164, 1120. **$[\alpha]_{25}^{D}$** = 1.4 (c = 1.0 in $CHCl_3$).

(*R*)-3-(5-methoxybenzofuran-2-yl)butanal 5c

The previously obtained terminal alkyne (200 mg, 1 mmol) was dissolved in dry THF (2 mL) and cooled to -20°C. A suspension of dicyclohexylborane (2 mmol) in dry THF (2 mL) was added and the resulting mixture was warmed to room temperature and stirred for 2 additional hours at room temperature at which point a solution of NaOH (1M, 5.5 mL) followed by H_2O_2 (30%, 1.9 mL) were added. The biphasic mixture was vigorously stirred for 3 hours at room temperature and after usual work up, the residue was purified by flash chromatography over silica gel (petroleum ether/diethyl 95:5 to 80:20) to afford aldehyde **5c**[18] (181 mg, 83%, 96%).

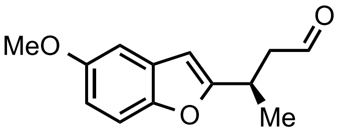

^{1}H NMR ($CDCl_3$, 400 MHz, δ (ppm)) 9.81 (t, *J* = 1.8 Hz, 1H), 7.31-7.27 (m, 1H), 6.96 (d, *J* = 2,4 Hz, 1H), 6.83 (dd, *J* = 8.8 Hz, *J* = 2.8 Hz, 1H), 6.37-6.35 (m, 1H), 3.83 (s, 3H), 3.60-3.48 (m, 1H), 2.80 (ddd, *J* = 17.3 Hz, *J* = 6.6 Hz, *J* = 1.8 Hz, 2H), 1.40 (d, *J* = 6.6 Hz, 3H). **^{13}C NMR** ($CDCl_3$, 100 MHz, δ (ppm)) 200.9, 162.1, 155.8, 149.6, 129.0, 112.0, 111.2, 103.3, 101.5, 55.9, 48.8, 28.2, 18.8. **IR (thin film)** ν = 2998, 2893, 2792, 2687, 1701, 1600, 1593, 1456, 1429, 1188, 1016. **$[\alpha]_{25}^{D}$** = -12.8 (c = 1.0 in $CHCl_3$). **Chiral HPLC**: Chiralpak® IC, 2% isopropanol in heptane, 0.8 $mL.min^{-1}$, λ = 254 nm, t_R = 19.0 min (minor), 21.1 min (major).

[18] Reiter, M.; Torssell, S.; Lee, S.; MacMillan, D. W. C. *Chem. Sci.* **2010**, *1*, 37.

Compound 4a

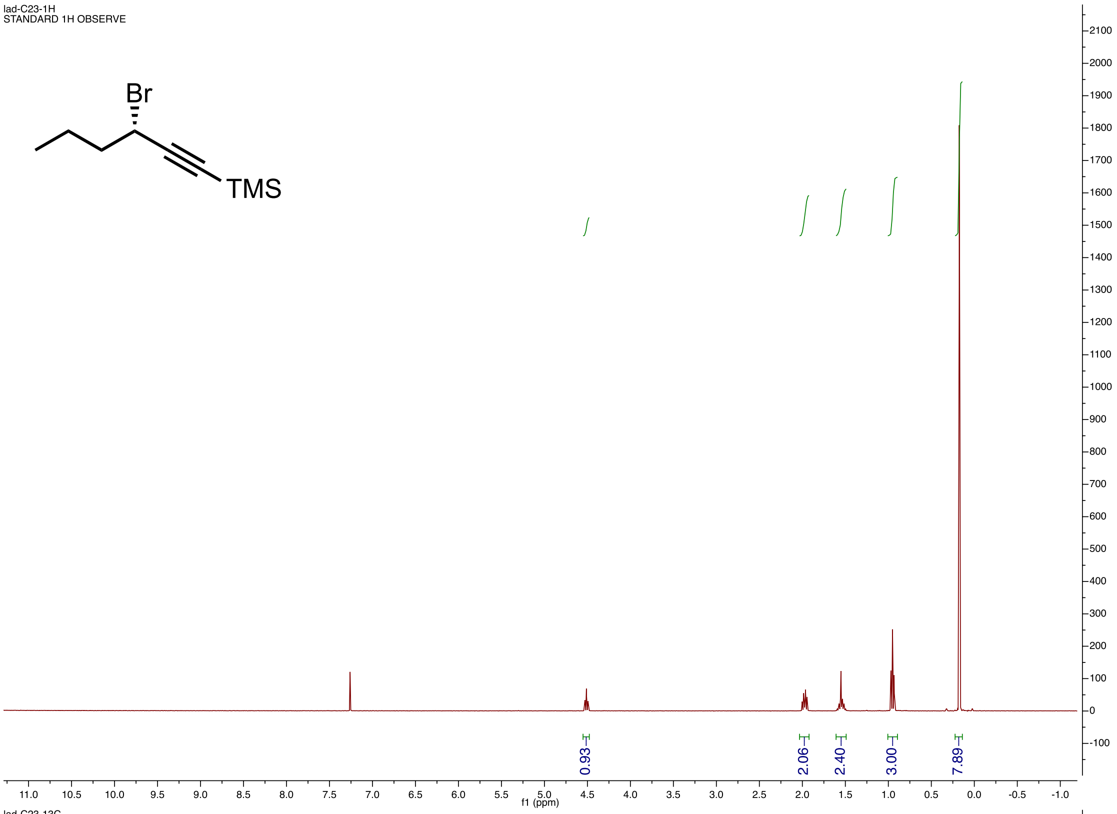
lad-C23-1H
STANDARD 1H OBSERVE
Br
TMS
0.93
2.06
2.40
3.00
7.89
f1 (ppm)

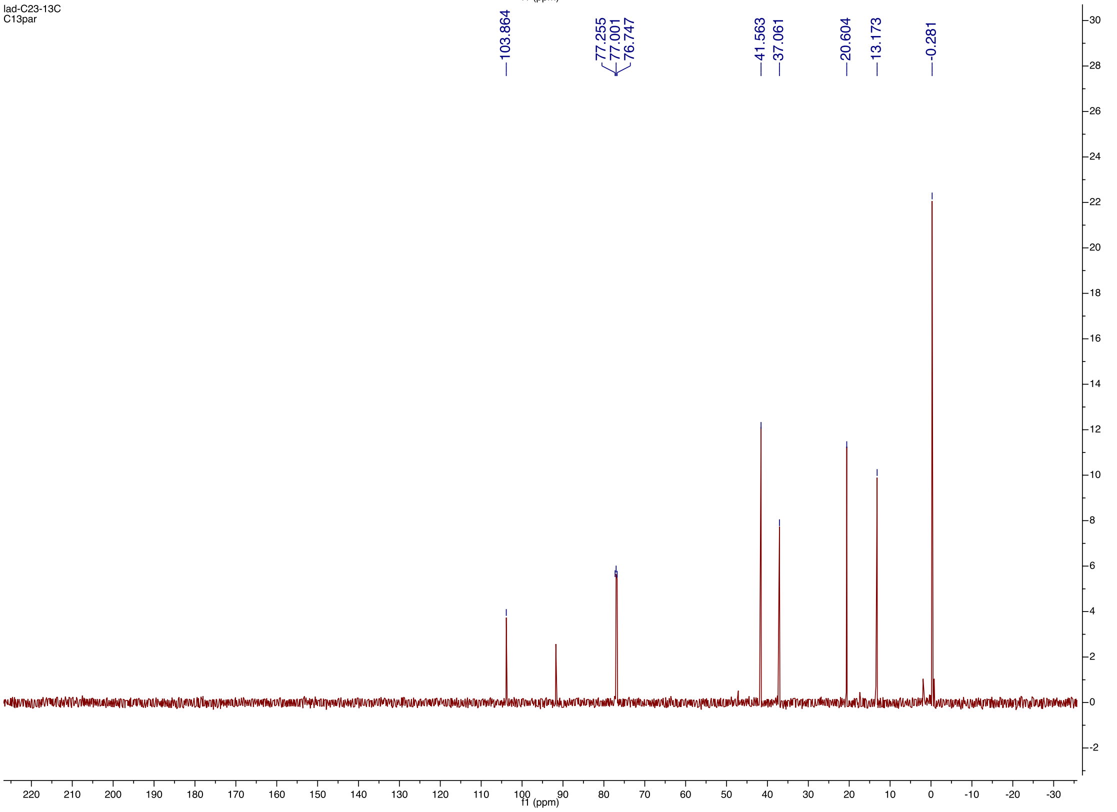
lad-C23-13C
C13par
103.864
77.255
77.001
76.747
41.563
37.061
20.604
13.173
-0.281
f1 (ppm)

Compound 4b

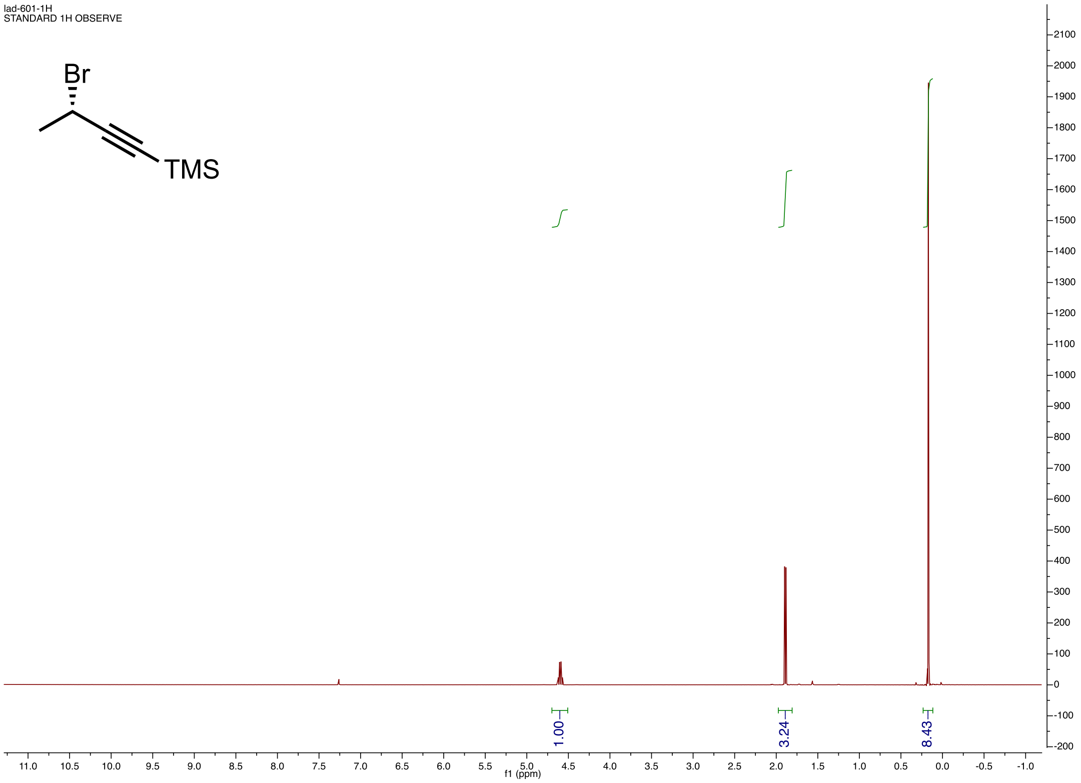
lad-601-1H
STANDARD 1H OBSERVE
Br
TMS
1.00
3.24
8.43
f1 (ppm)

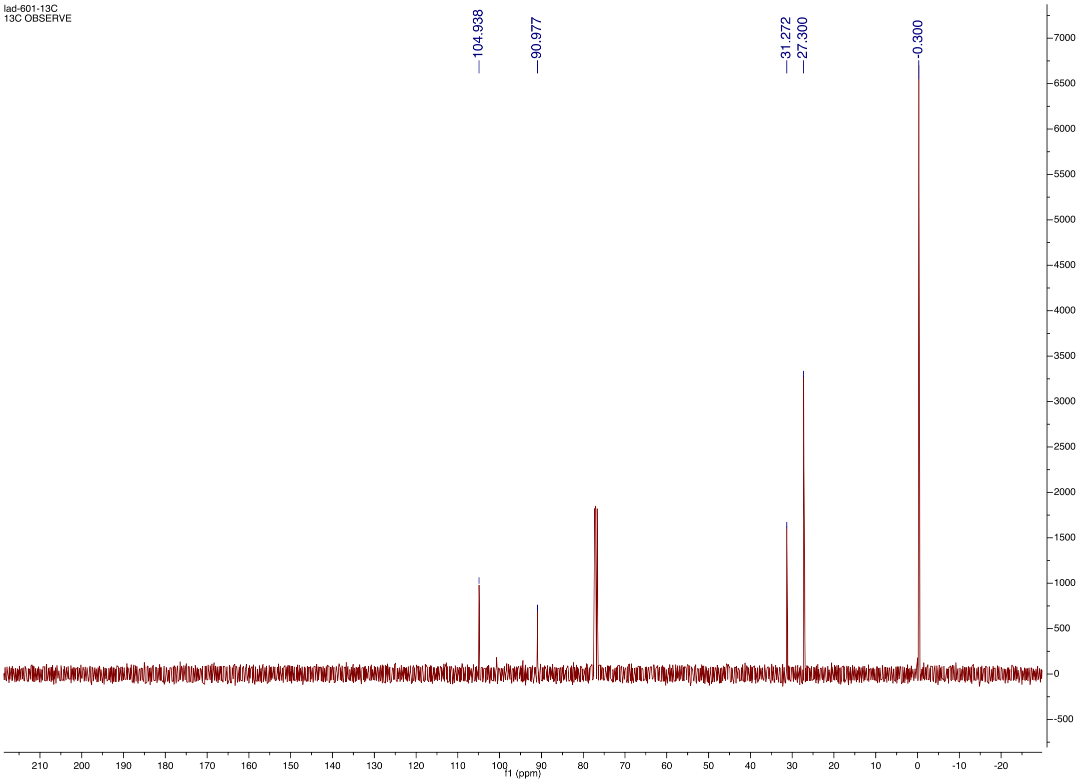
lad-601-13C
13C OBSERVE
104.938
90.977
31.272
27.300
-0.300
f1 (ppm)

Compound 4c dr = 1:1

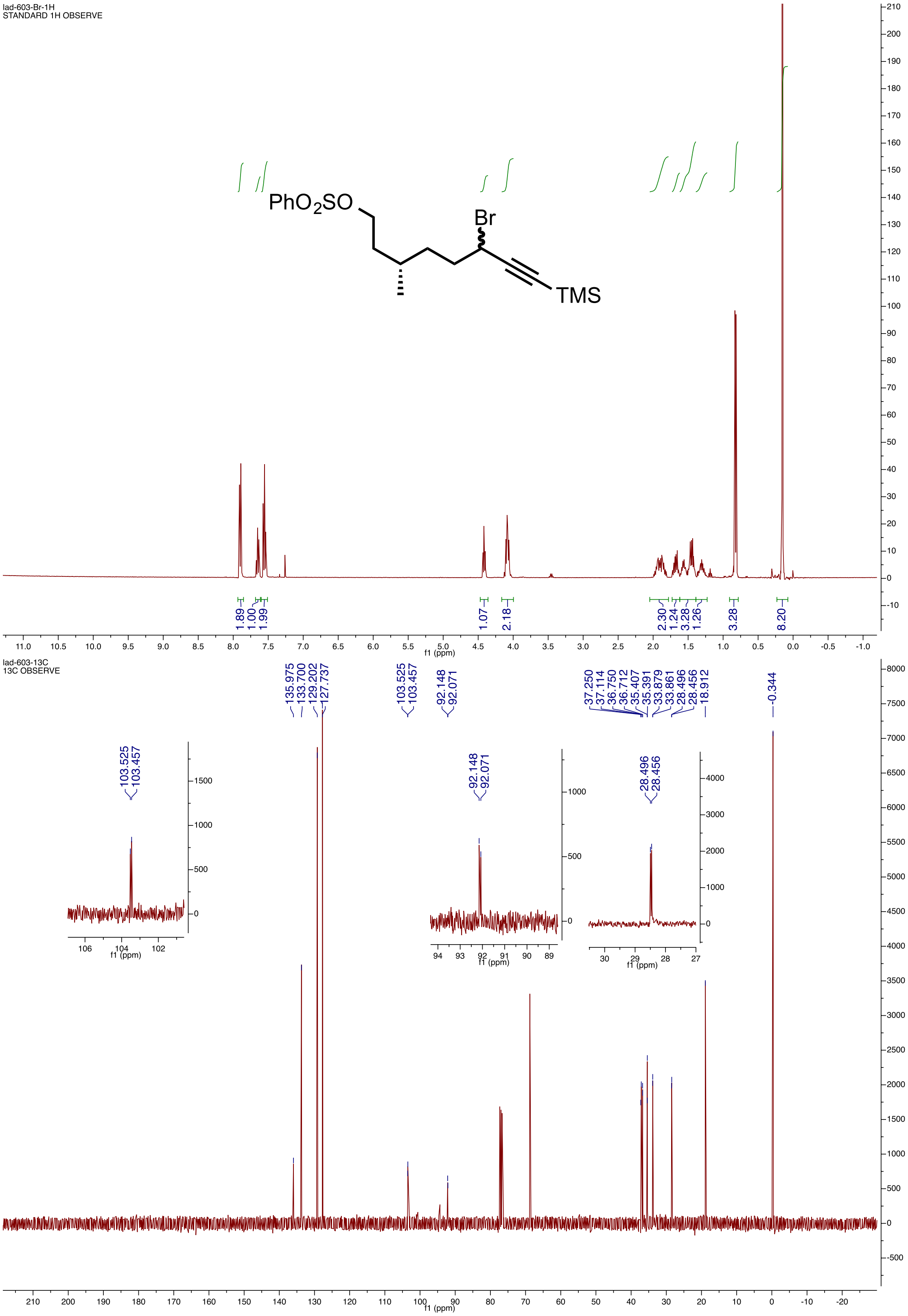

Compound 4c dr > 95:5

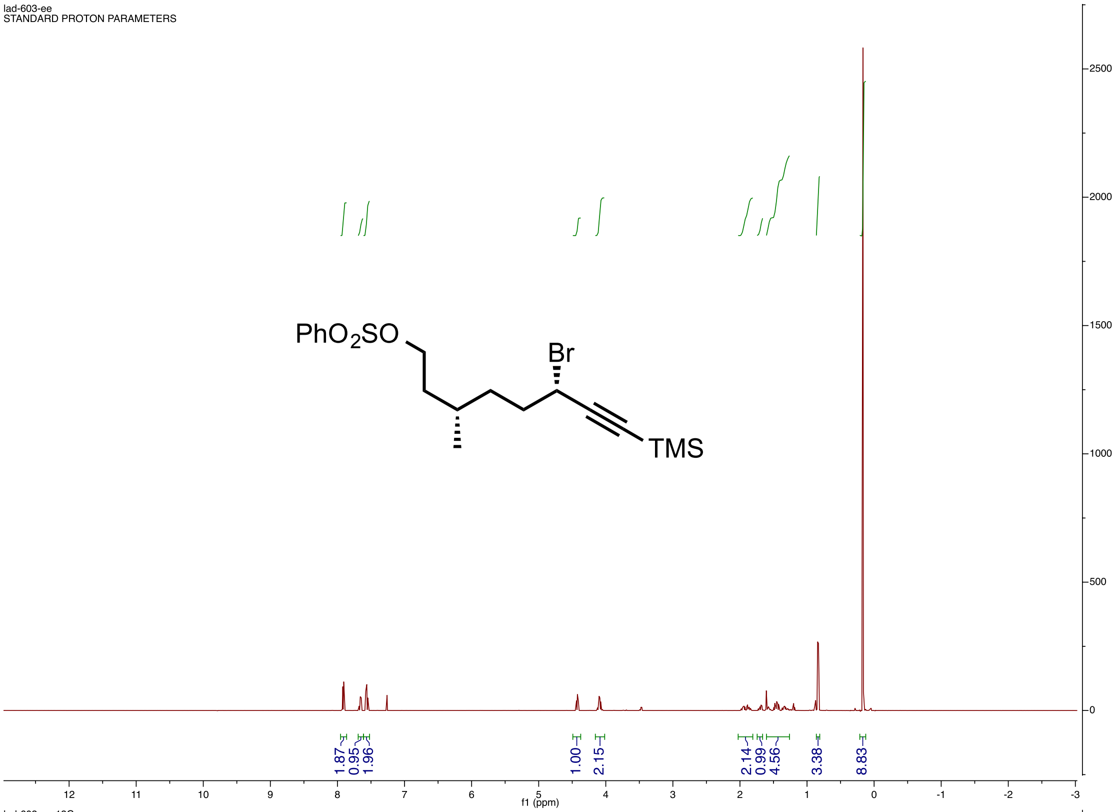

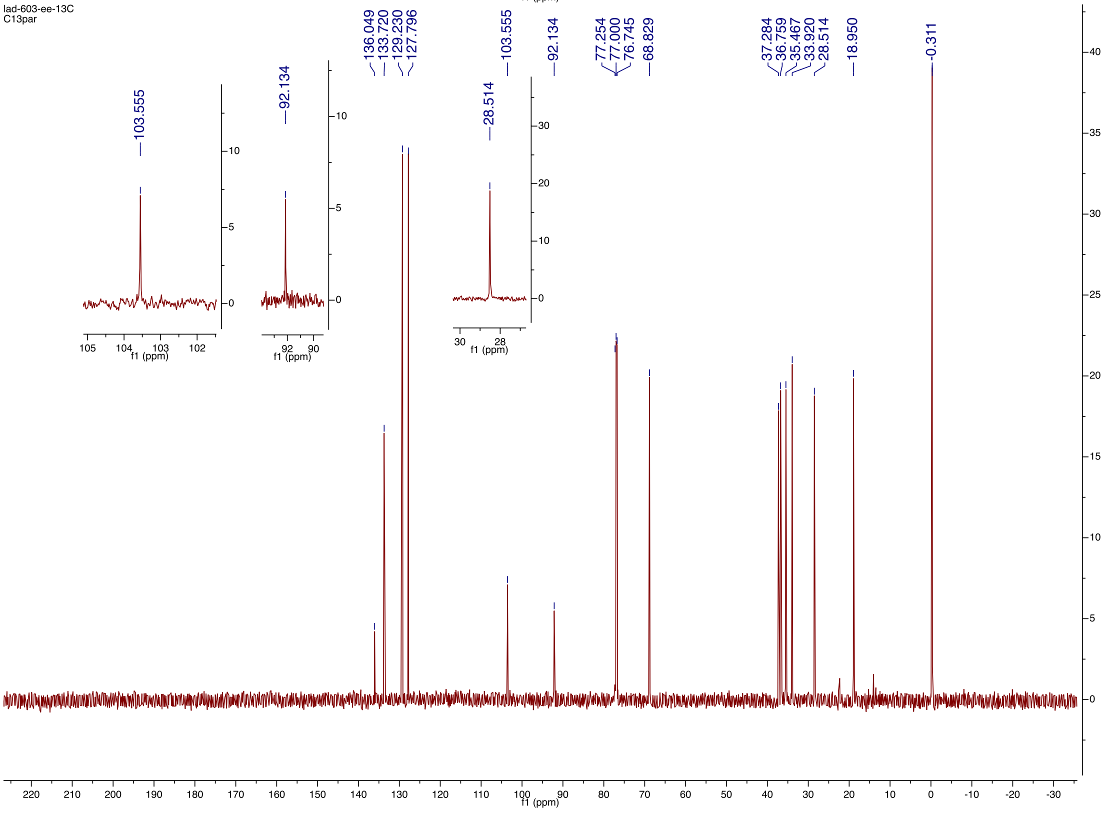

Compound 4d

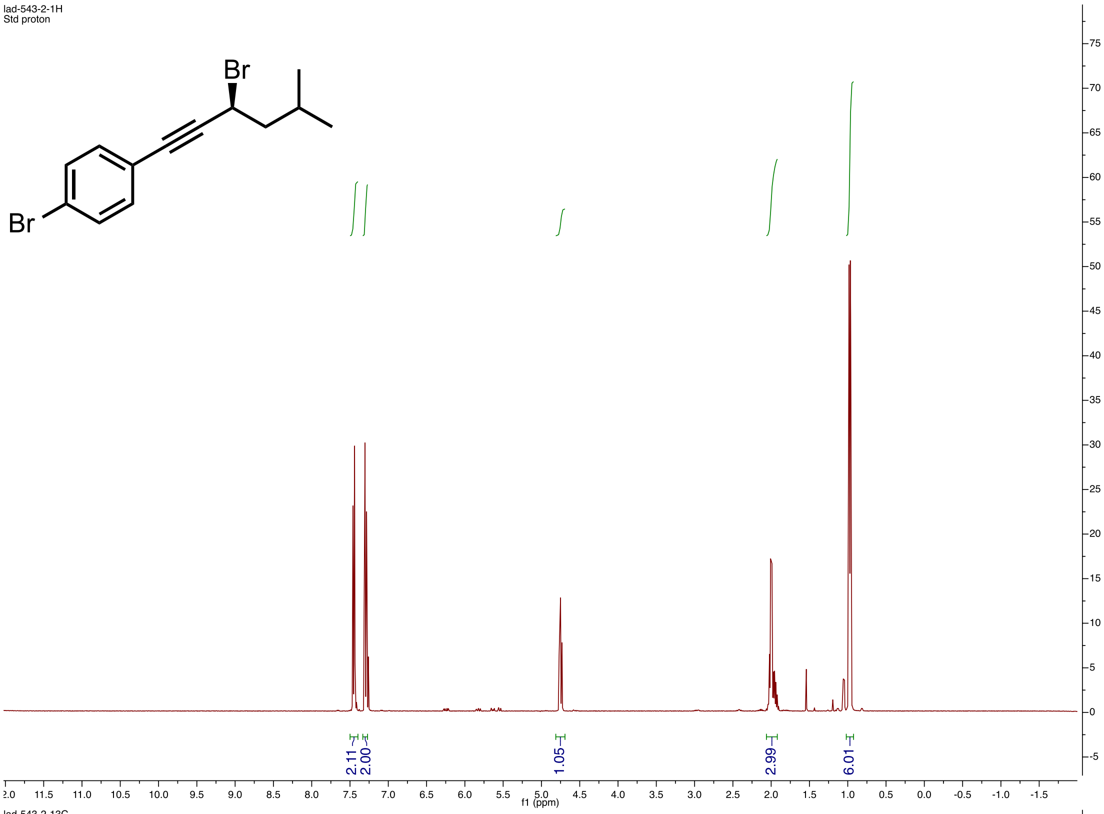

lad-543-2-1H
Std proton
Br
Br
2.11
2.00
1.05
2.99
6.01
f1 (ppm)

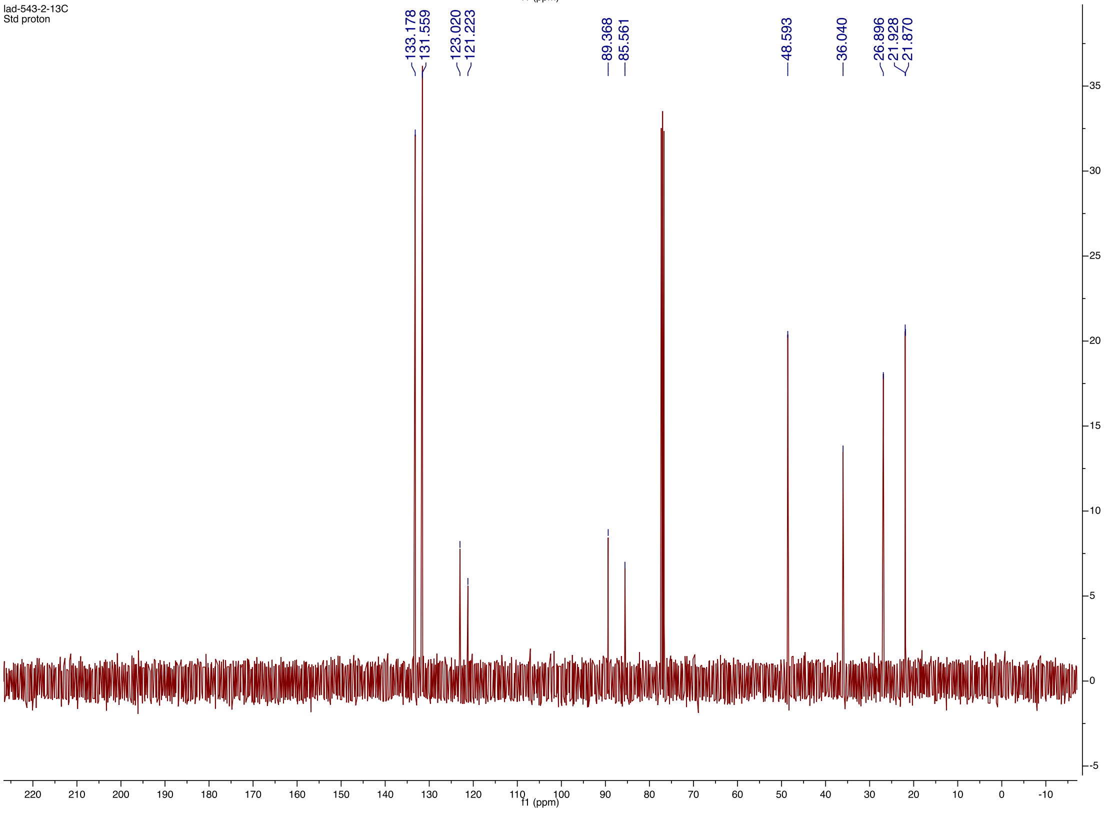

lad-543-2-13C
Std proton
133.178
131.559
123.020
121.223
89.368
85.561
48.593
36.040
26.896
21.928
21.870
f1 (ppm)

Compound 4e

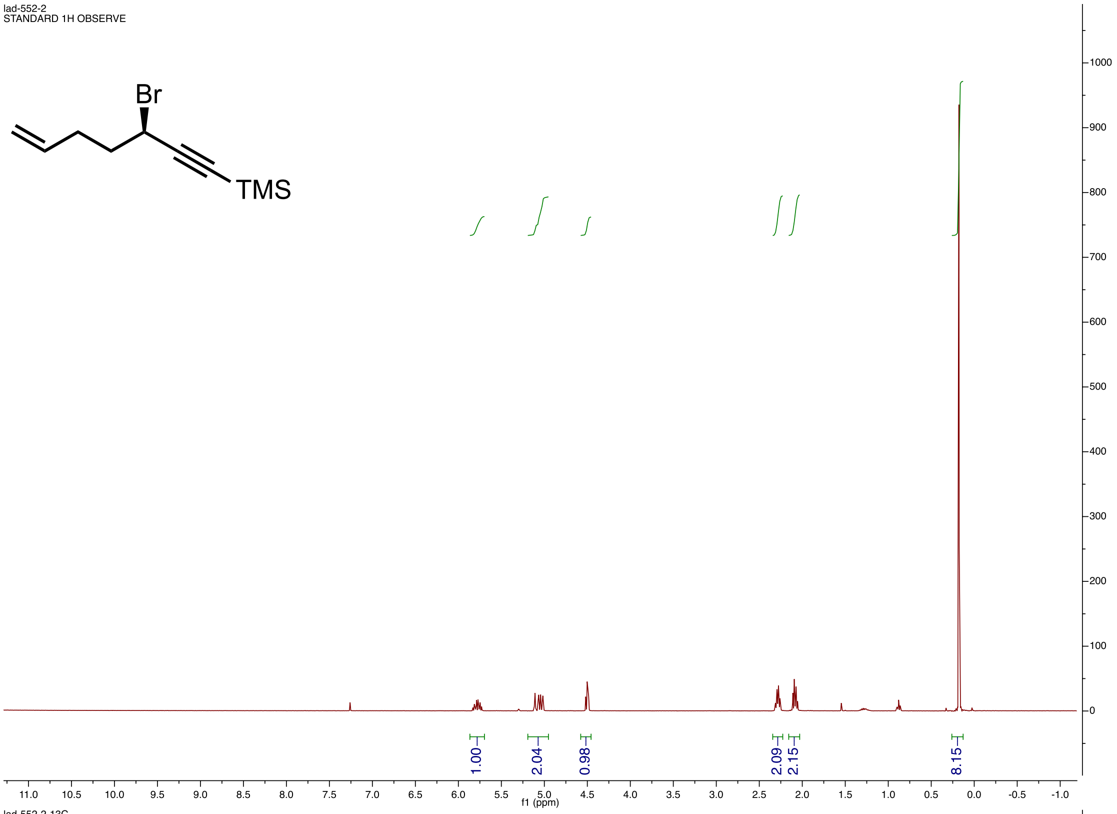

lad-552-2
STANDARD 1H OBSERVE
Br
TMS
1.00
2.04
0.98
2.09
2.15
8.15
f1 (ppm)

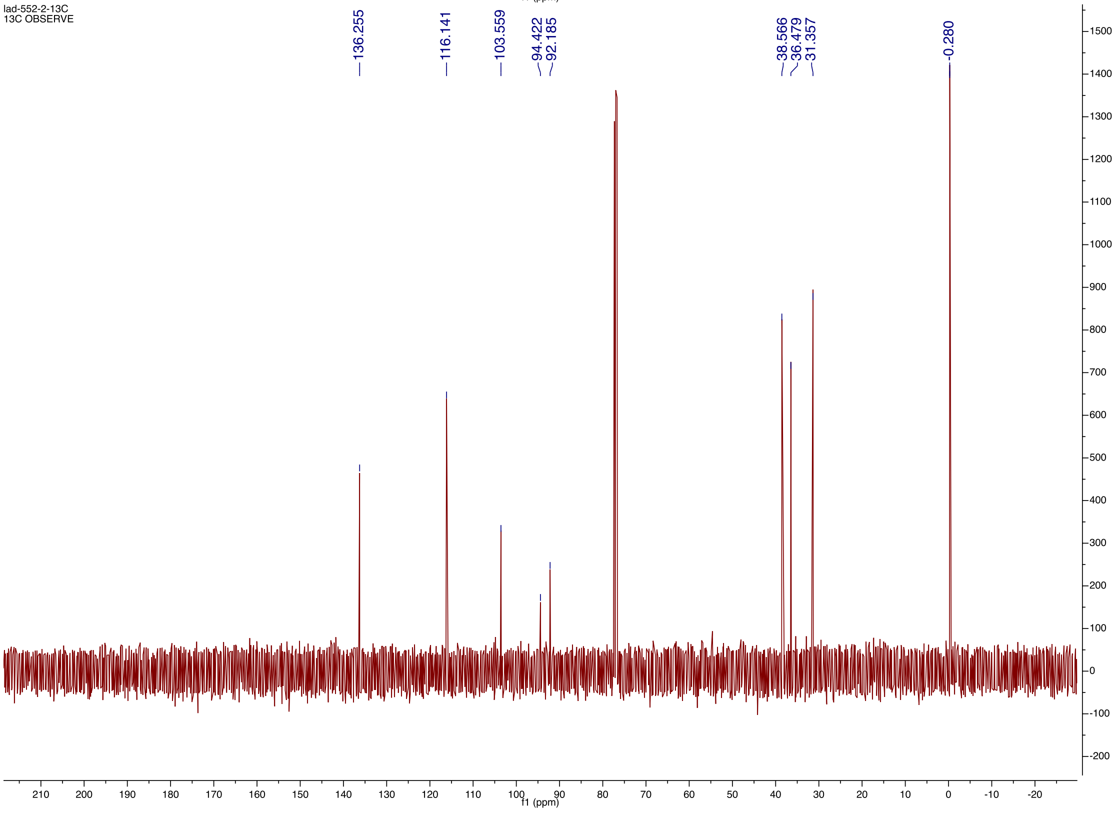

lad-552-2-13C
13C OBSERVE
136.255
116.141
103.559
94.422
92.185
38.566
36.479
31.357
-0.280
f1 (ppm)

Compound 4f

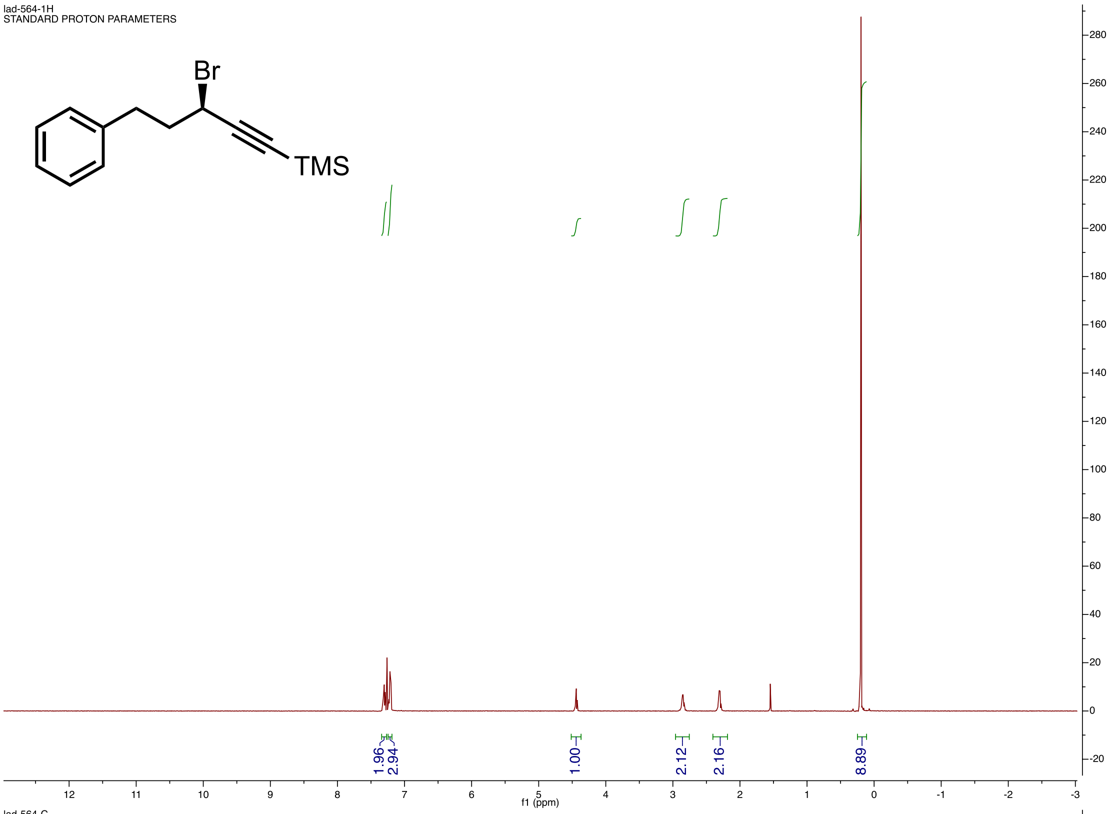
lad-564-1H
STANDARD PROTON PARAMETERS
Br
TMS
1.96
2.94
1.00
2.12
2.16
8.89
f1 (ppm)

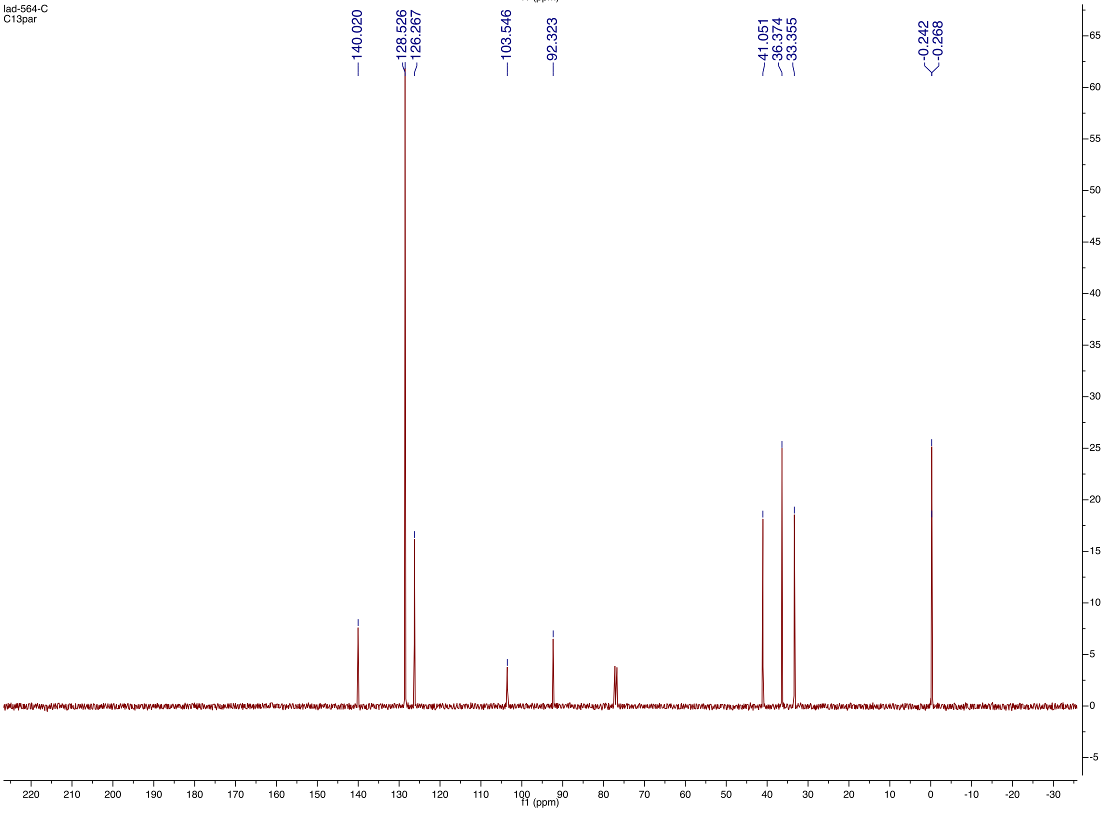
lad-564-C
C13par
140.020
128.526
126.267
103.546
92.323
41.051
36.374
33.355
-0.242
-0.268
f1 (ppm)

Compound 4g

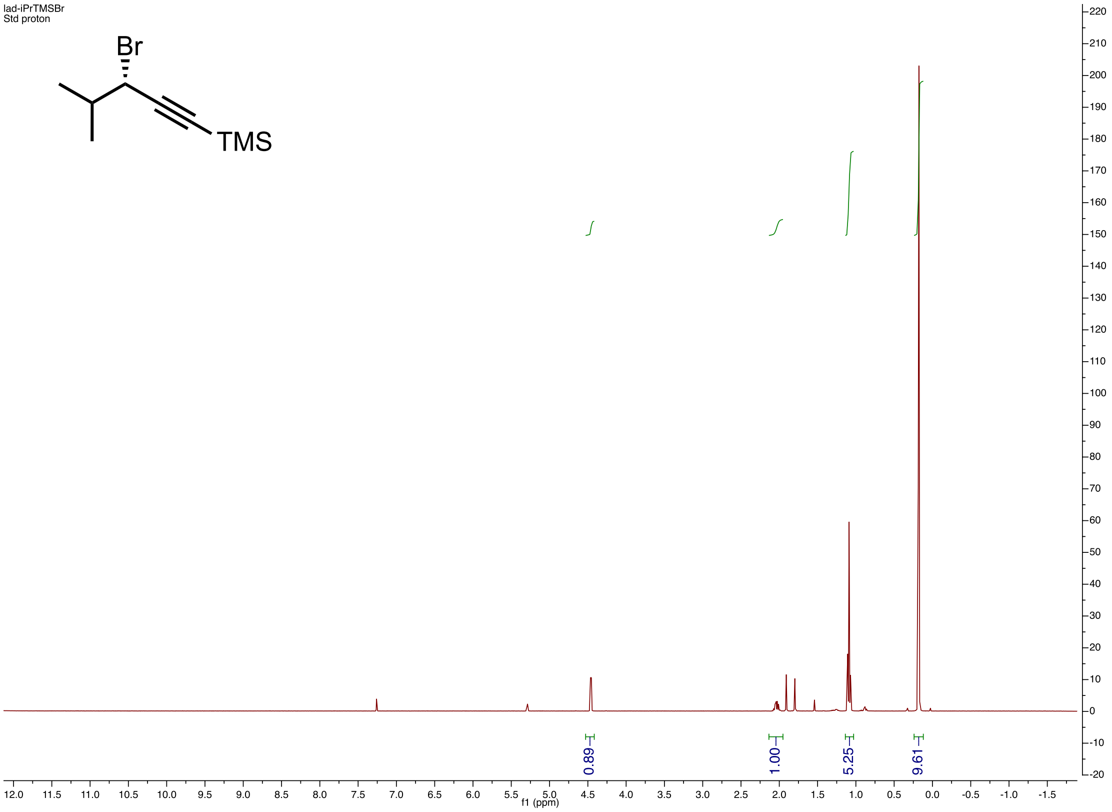
lad-iPrTMSBr
Std proton
Br
TMS
0.89
1.00
5.25
9.61
f1 (ppm)

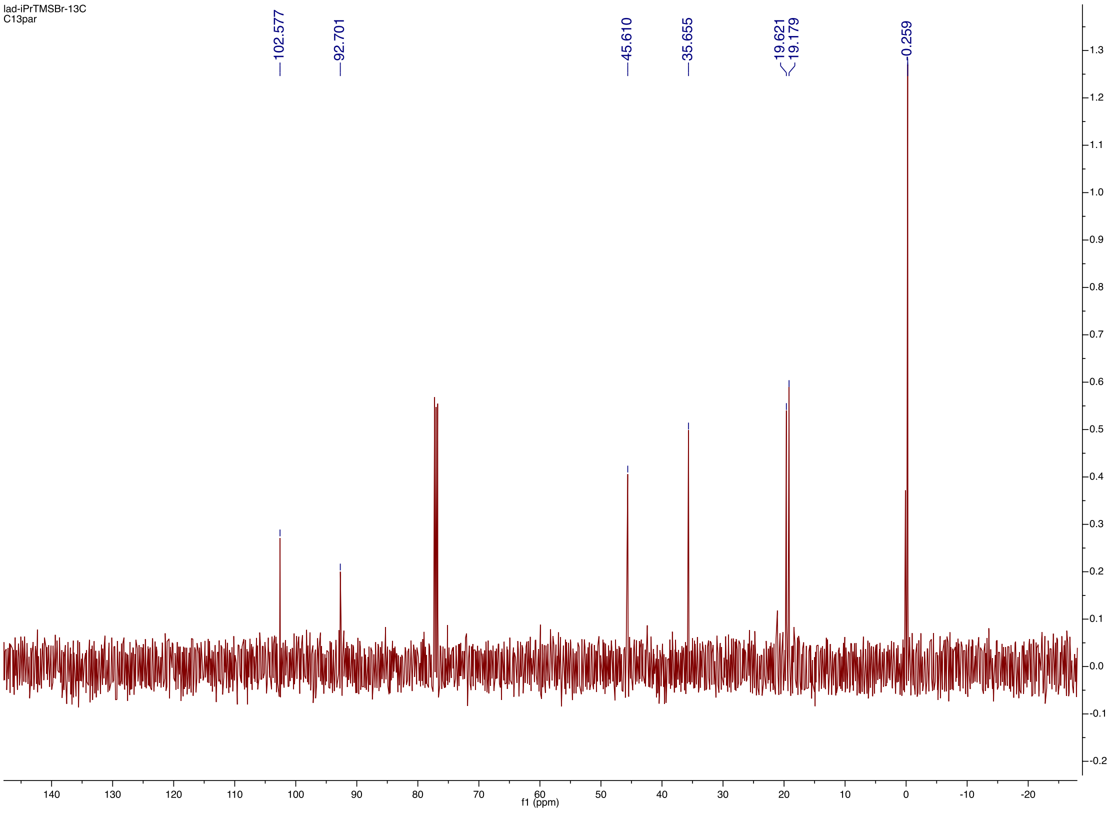
lad-iPrTMSBr-13C
C13par
102.577
92.701
45.610
35.655
19.621
19.179
0.259
f1 (ppm)

Compound 4h

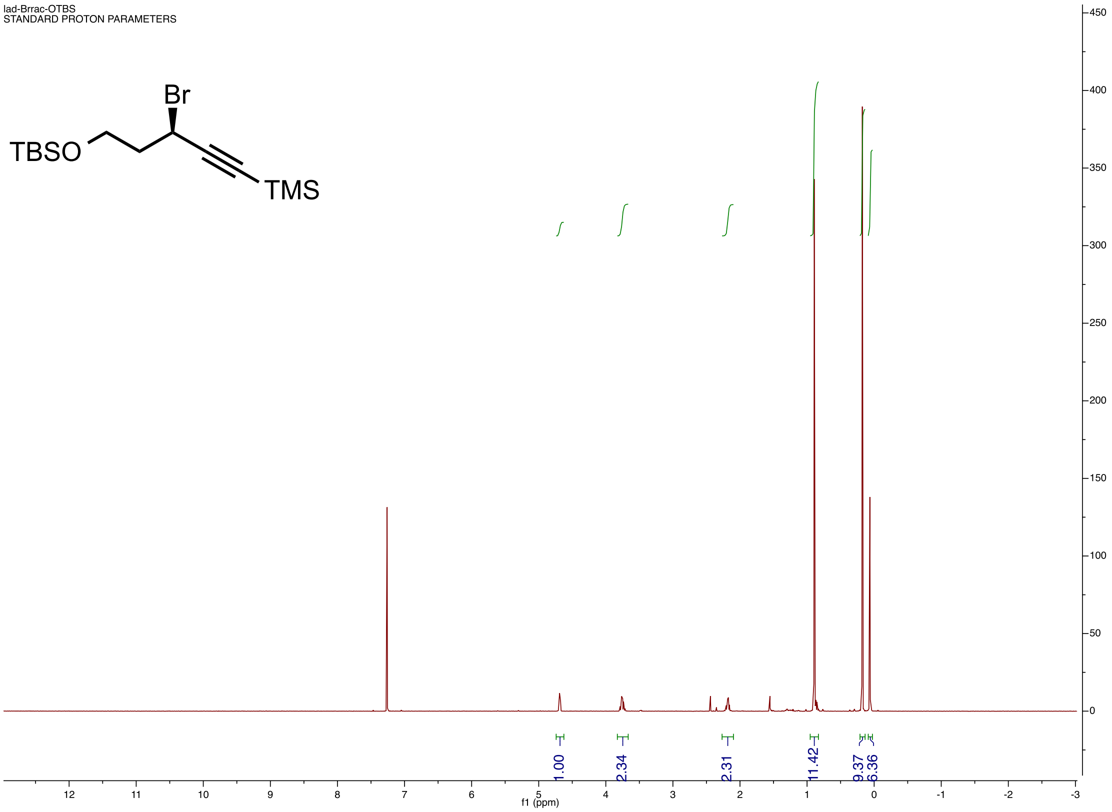

lad-Brrac-OTBS
STANDARD PROTON PARAMETERS
Br
TBSO
TMS
1.00
2.34
2.31
11.42
9.37
6.36
f1 (ppm)

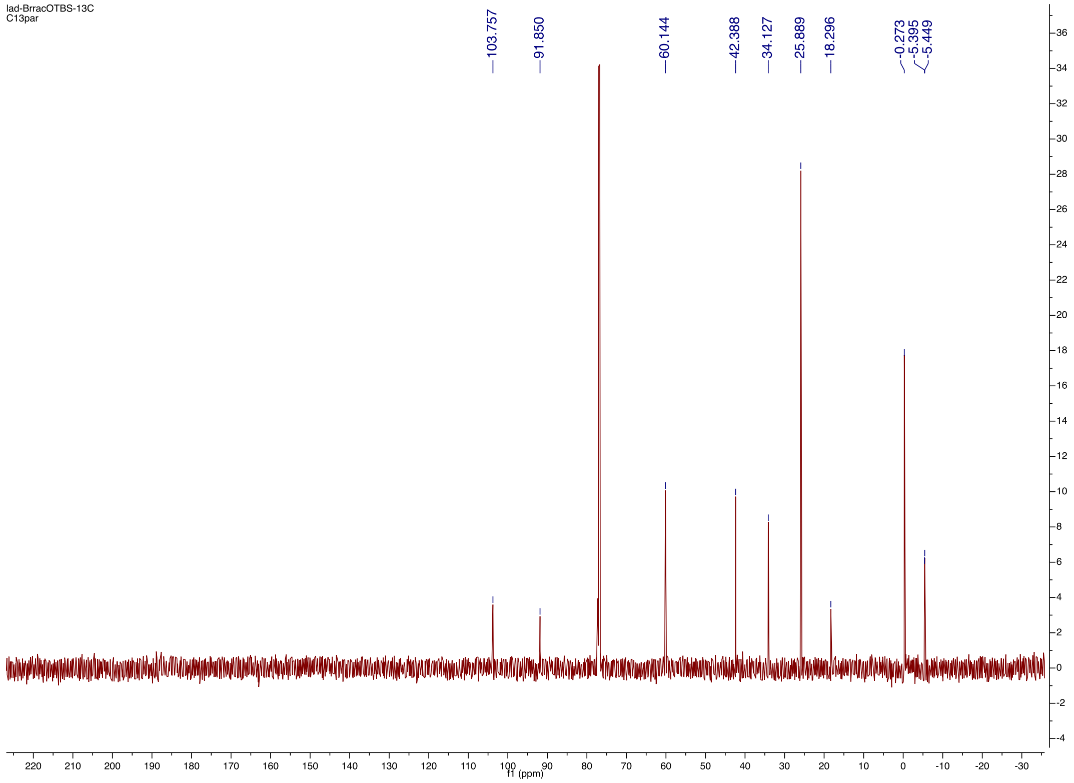

lad-BrracOTBS-13C
C13par
103.757
91.850
60.144
42.388
34.127
25.889
18.296
-0.273
-5.395
-5.449
f1 (ppm)

Compound 4i

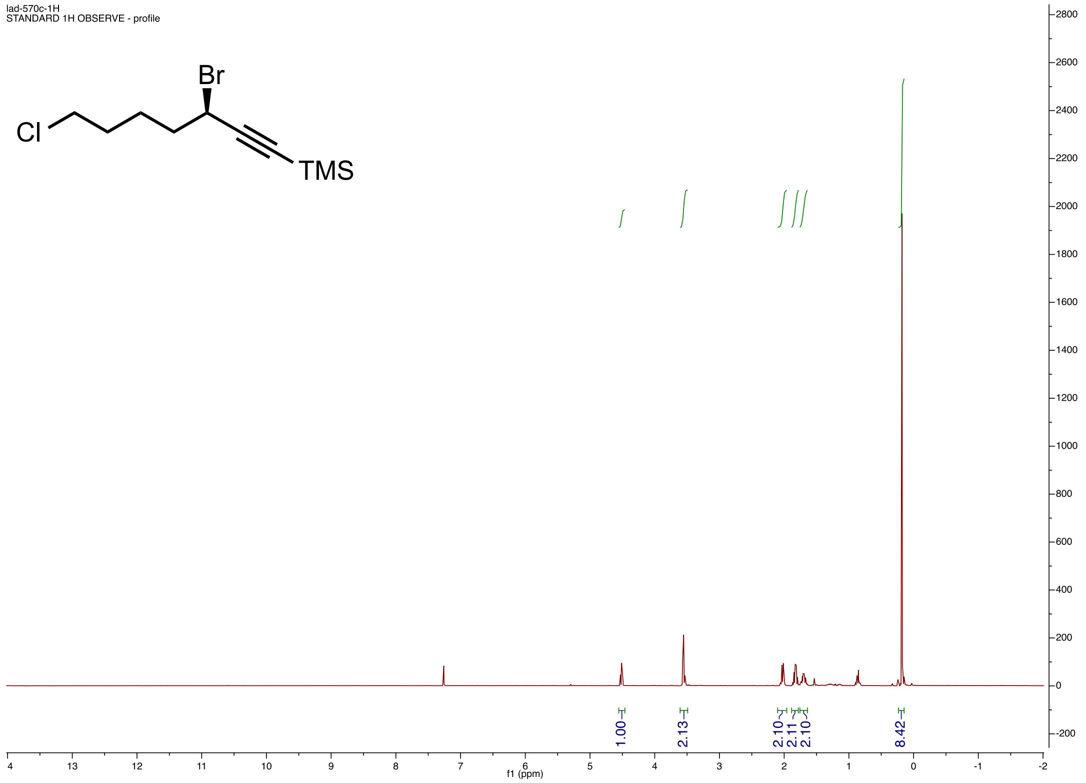
lad-570c-1H
STANDARD 1H OBSERVE - profile
Br
Cl
TMS
1.00
2.13
2.10
2.11
2.10
8.42
f1 (ppm)

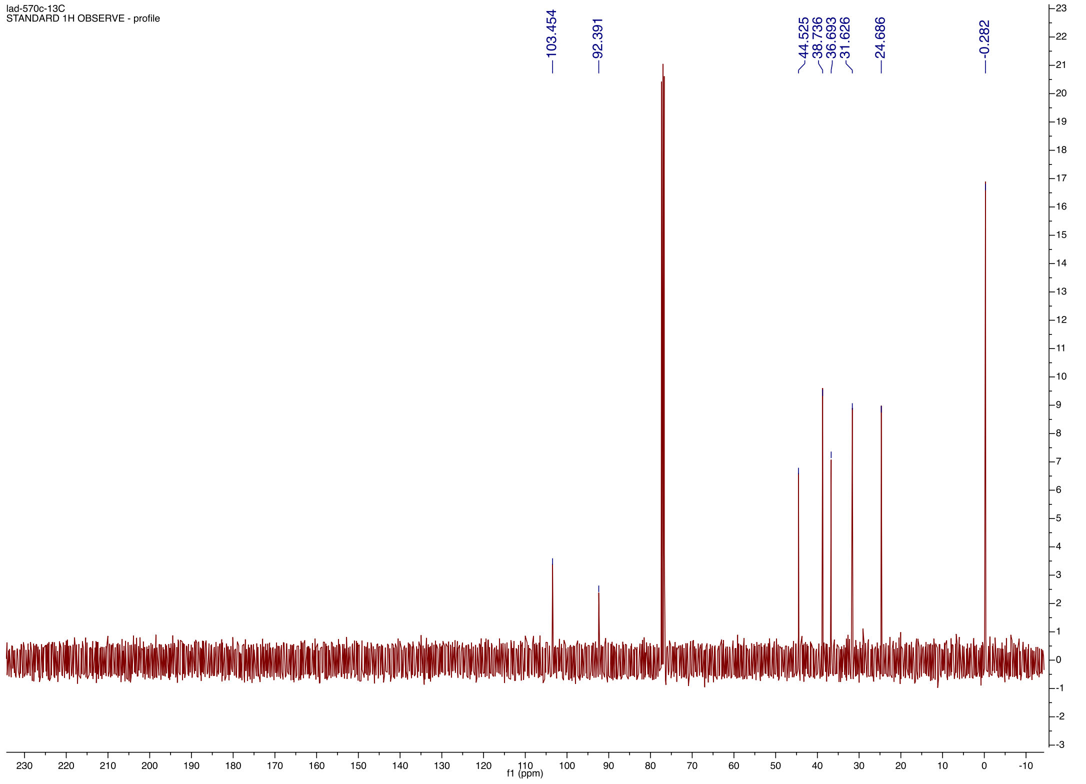
lad-570c-13C
STANDARD 1H OBSERVE - profile
103.454
92.391
44.525
38.736
36.693
31.626
24.686
0.282
f1 (ppm)

Compound 4j

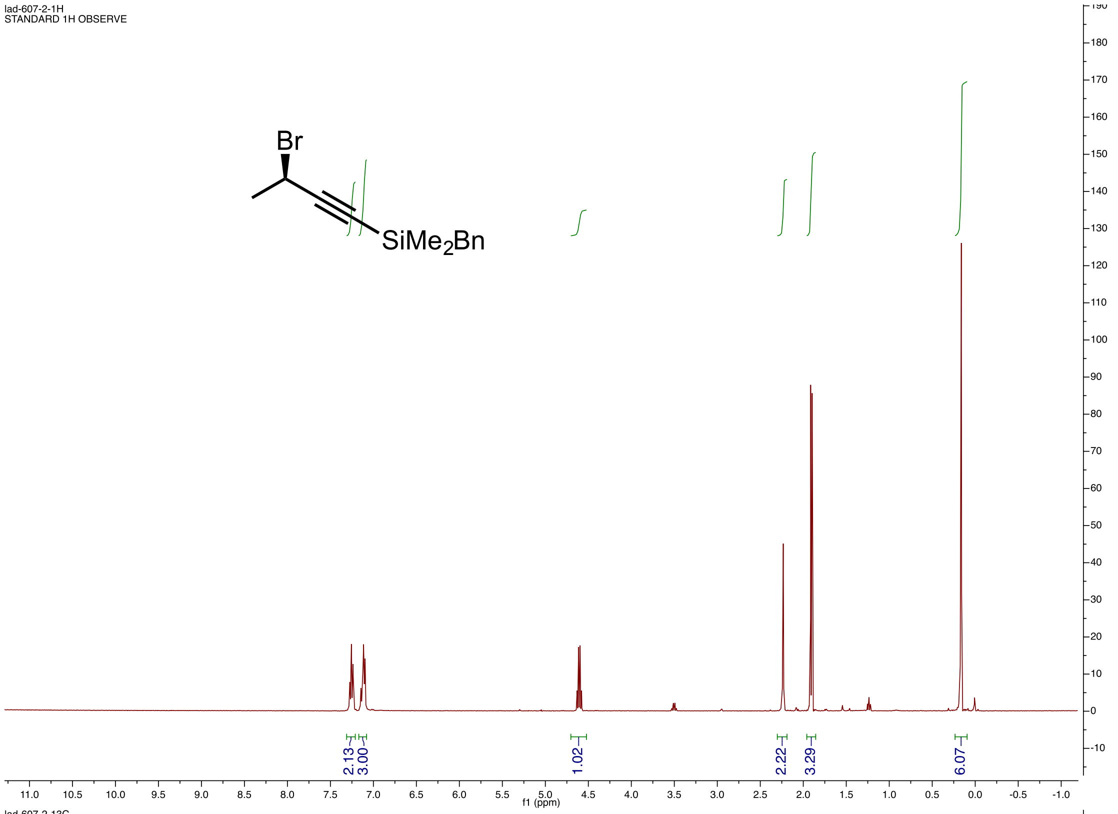
lad-607-2-1H
STANDARD 1H OBSERVE
Br
SiMe2Bn
2.13
3.00
1.02
2.22
3.29
6.07
f1 (ppm)

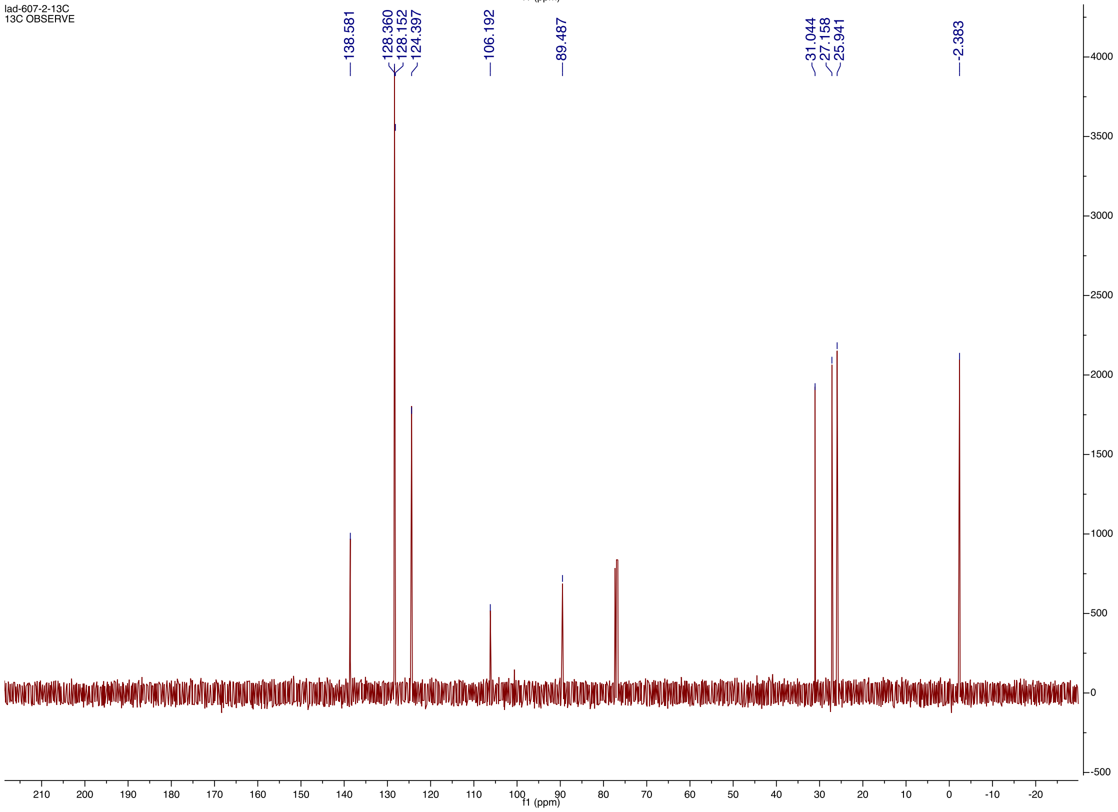
lad-607-2-13C
13C OBSERVE
138.581
128.360
128.152
124.397
106.192
89.487
31.044
27.158
25.941
-2.383
f1 (ppm)

Compound 4k

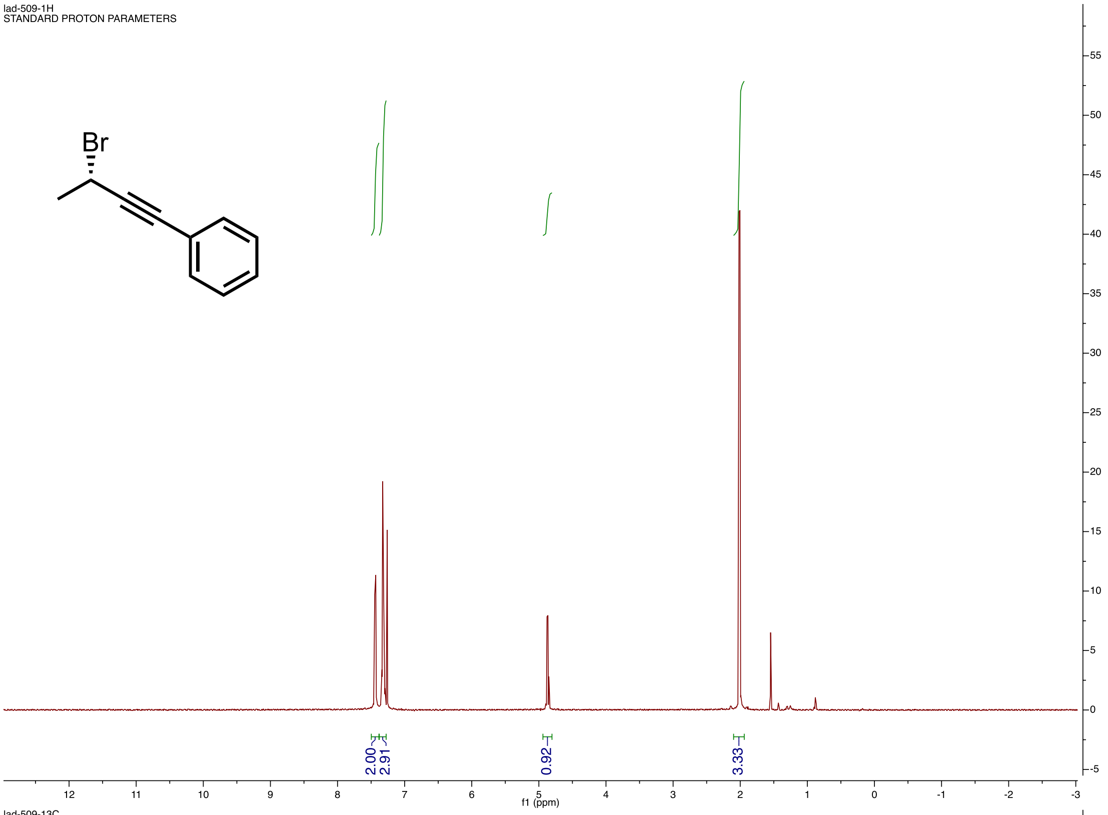
lad-509-1H
STANDARD PROTON PARAMETERS
Br
2.00
2.91
0.92
3.33
f1 (ppm)

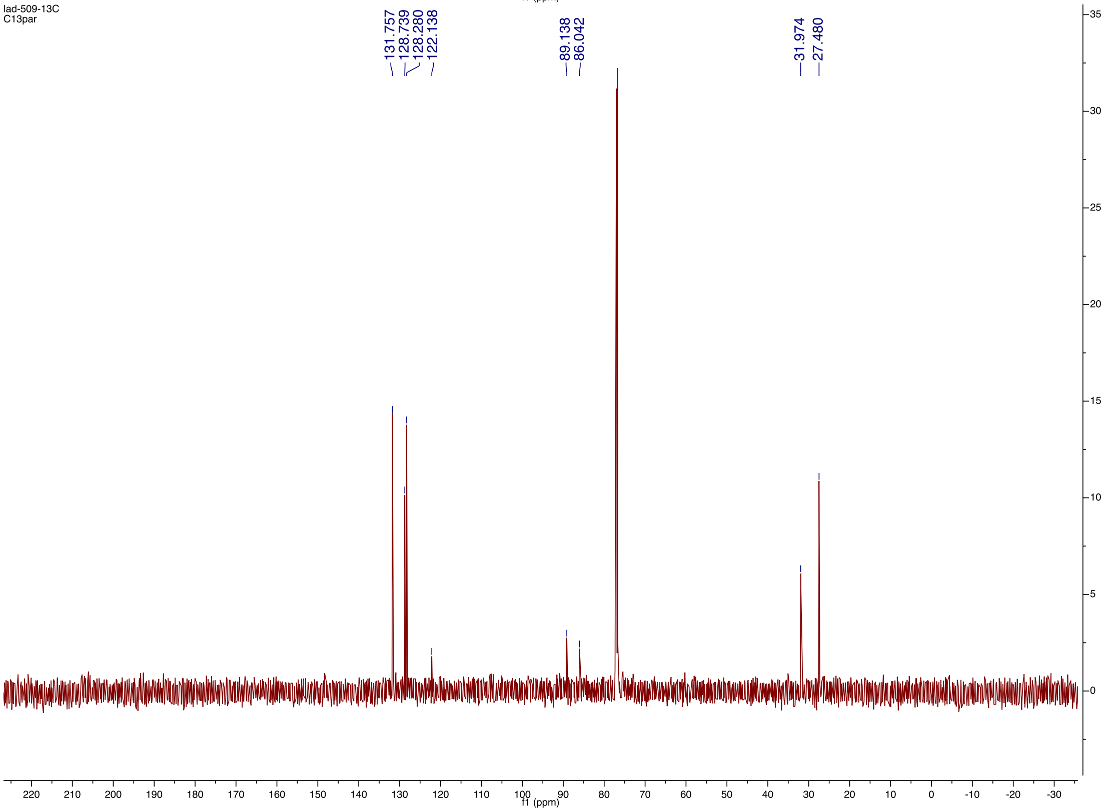
lad-509-13C
C13par
131.757
128.739
128.280
122.138
89.138
86.042
31.974
27.480
f1 (ppm)

Compound 2a

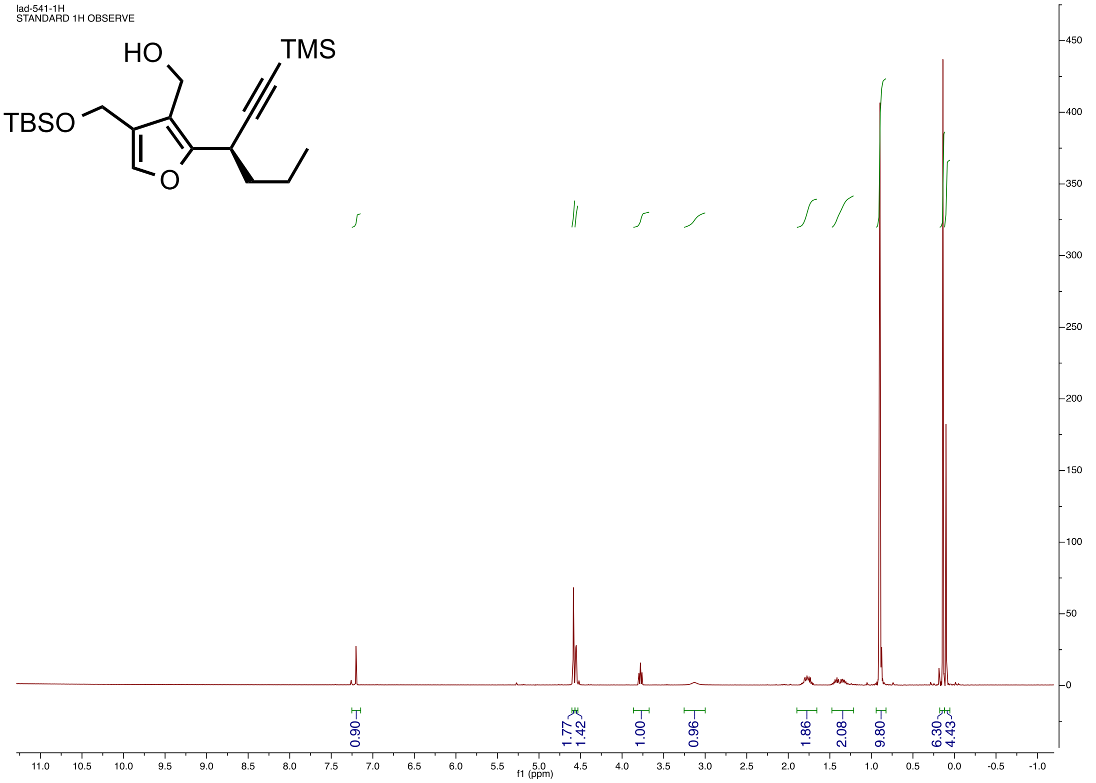
lad-541-1H
STANDARD 1H OBSERVE
HO
TMS
TBSO
O
0.90
1.77
1.42
1.00
0.96
1.86
2.08
9.80
6.30
4.43
f1 (ppm)

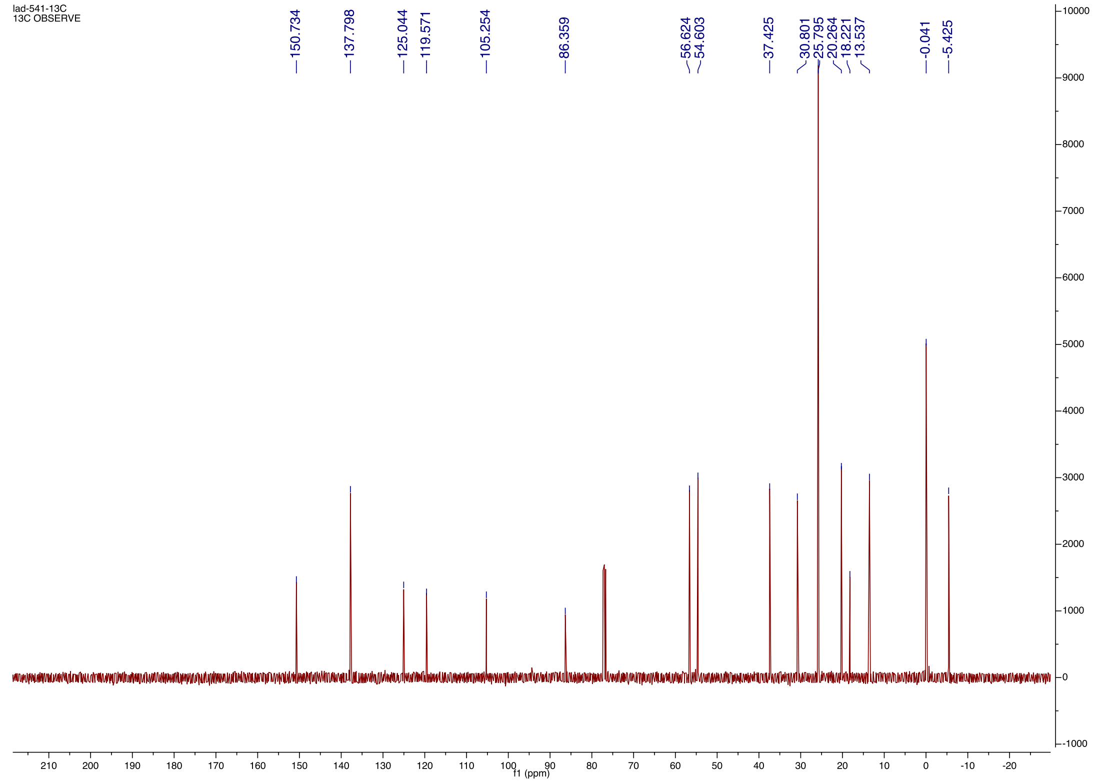
lad-541-13C
13C OBSERVE
150.734
137.798
125.044
119.571
105.254
86.359
56.624
54.603
37.425
30.801
25.795
20.264
18.221
13.537
-0.041
-5.425
f1 (ppm)

Compound 2b

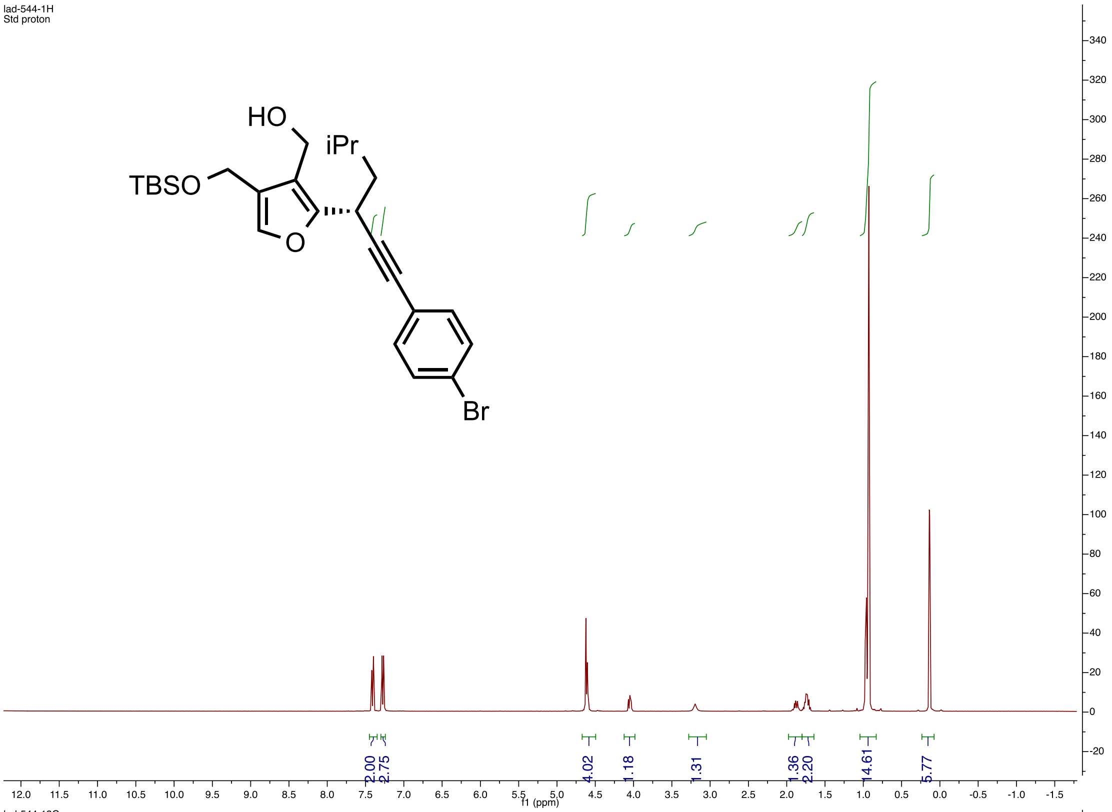
lad-544-1H
Std proton
HO
iPr
TBSO
O
Br
2.00
2.75
4.02
1.18
1.31
1.36
2.20
14.61
5.77
f1 (ppm)

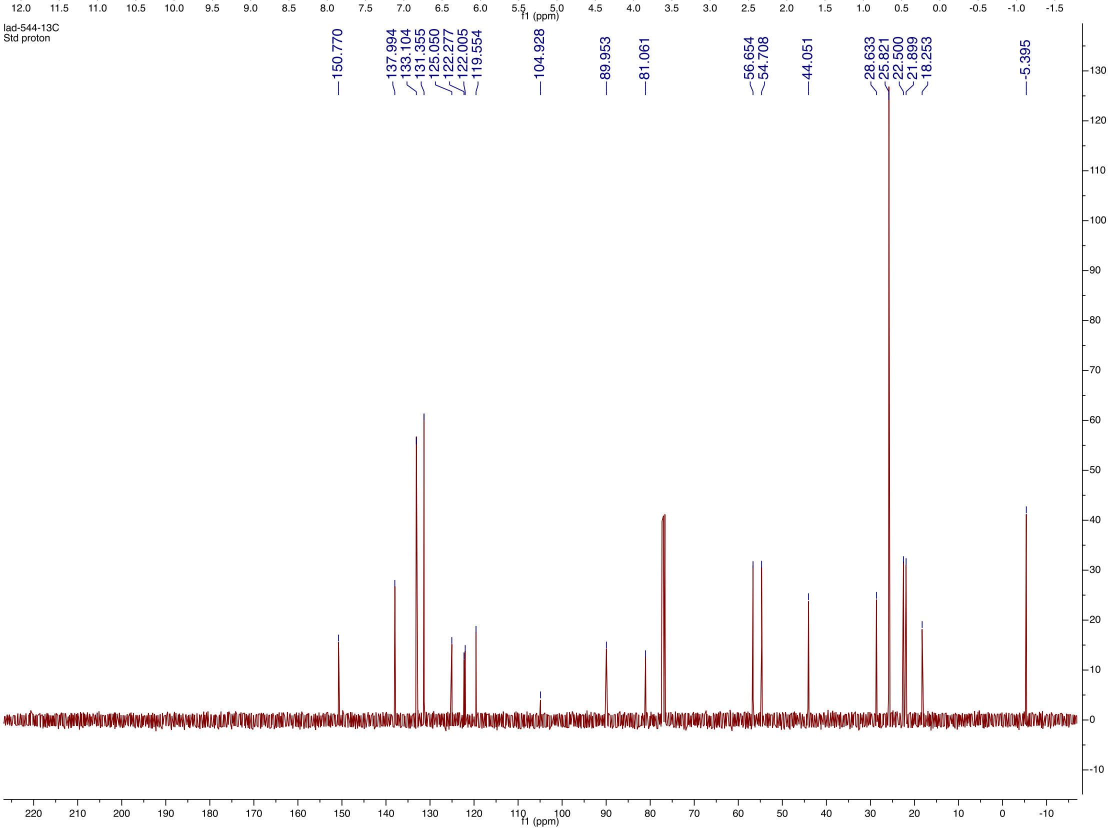
lad-544-13C
Std proton
150.770
137.994
133.104
131.355
125.050
122.277
122.005
119.554
104.928
89.953
81.061
56.654
54.708
44.051
28.633
25.821
22.500
21.899
18.253
-5.395
f1 (ppm)

Compound 2c

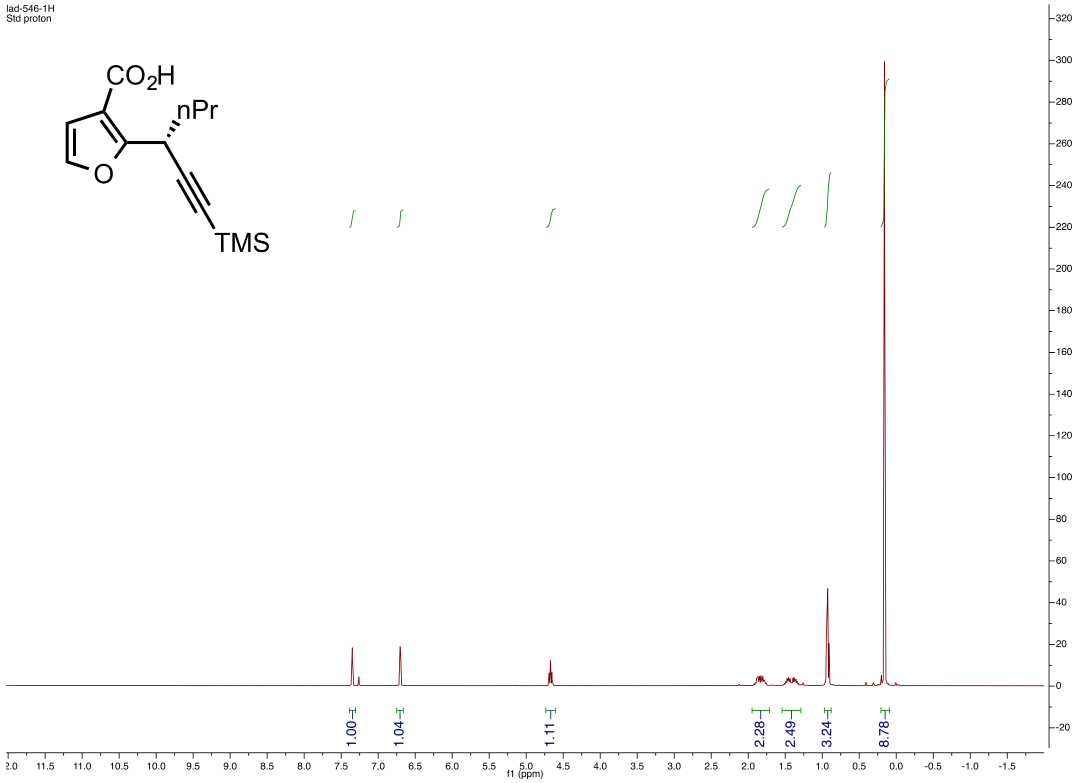

lad-546-1H
Std proton
CO2H
nPr
O
TMS
1.00
1.04
1.11
2.28
2.49
3.24
8.78
f1 (ppm)

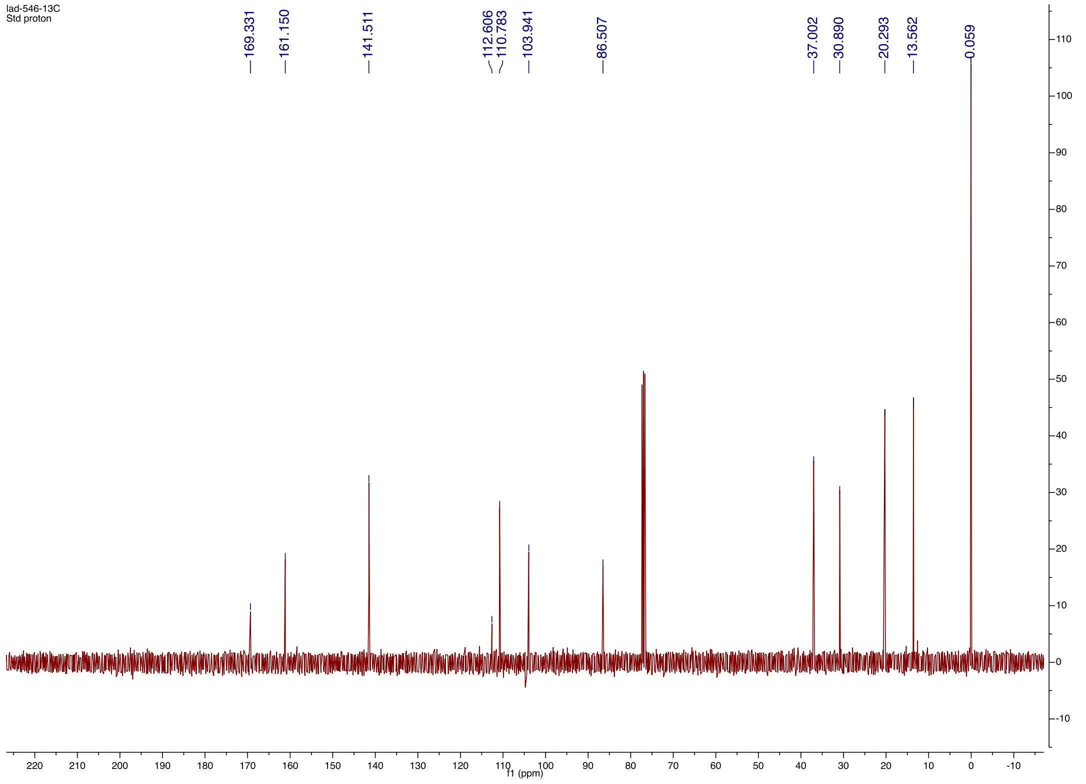

lad-546-13C
Std proton
169.331
161.150
141.511
112.606
110.783
103.941
86.507
37.002
30.890
20.293
13.562
0.059
f1 (ppm)

Compound 2d

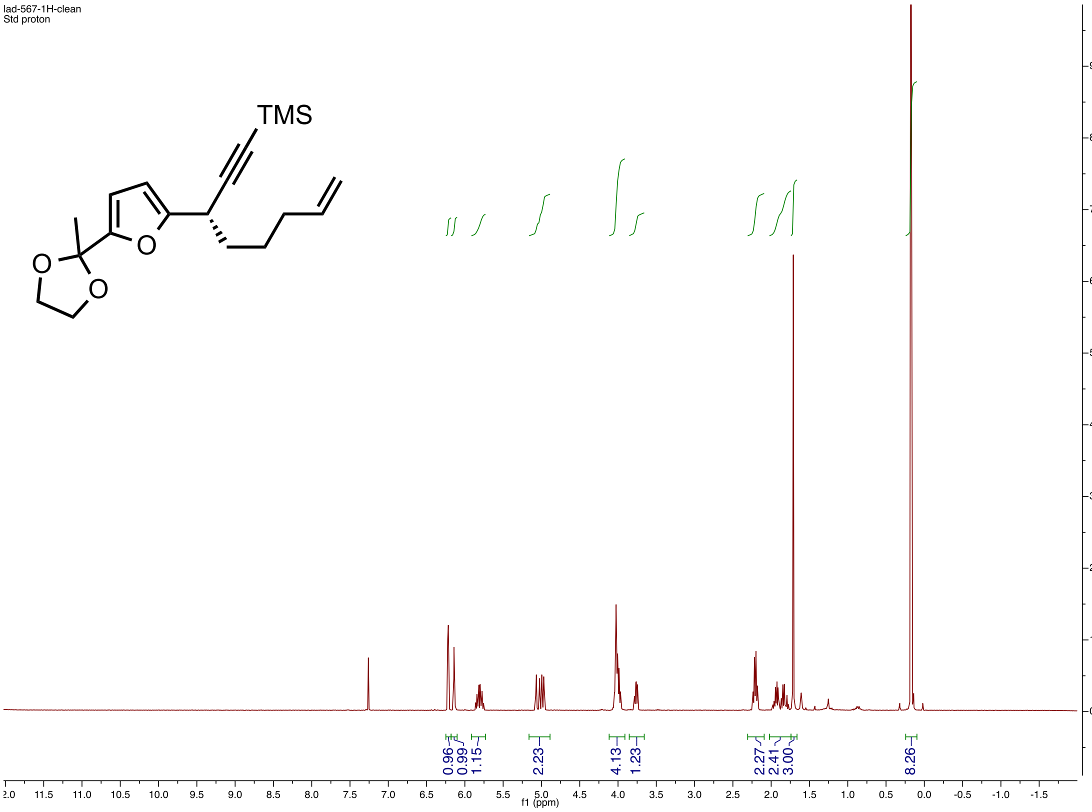
lad-567-1H-clean
Std proton
TMS
0.96
0.99
1.15
2.23
4.13
1.23
2.27
2.41
3.00
8.26
f1 (ppm)

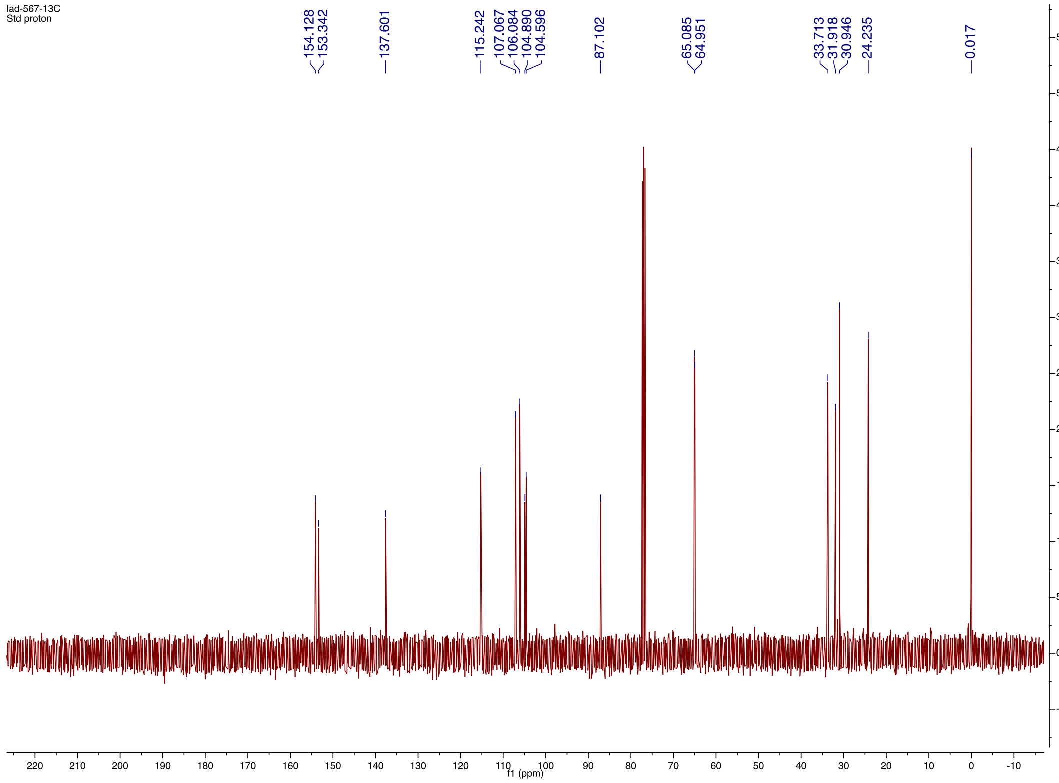
lad-567-13C
Std proton
154.128
153.342
137.601
115.242
107.067
106.084
104.890
104.596
87.102
65.085
64.951
33.713
31.918
30.946
24.235
0.017
f1 (ppm)

Compound 2e

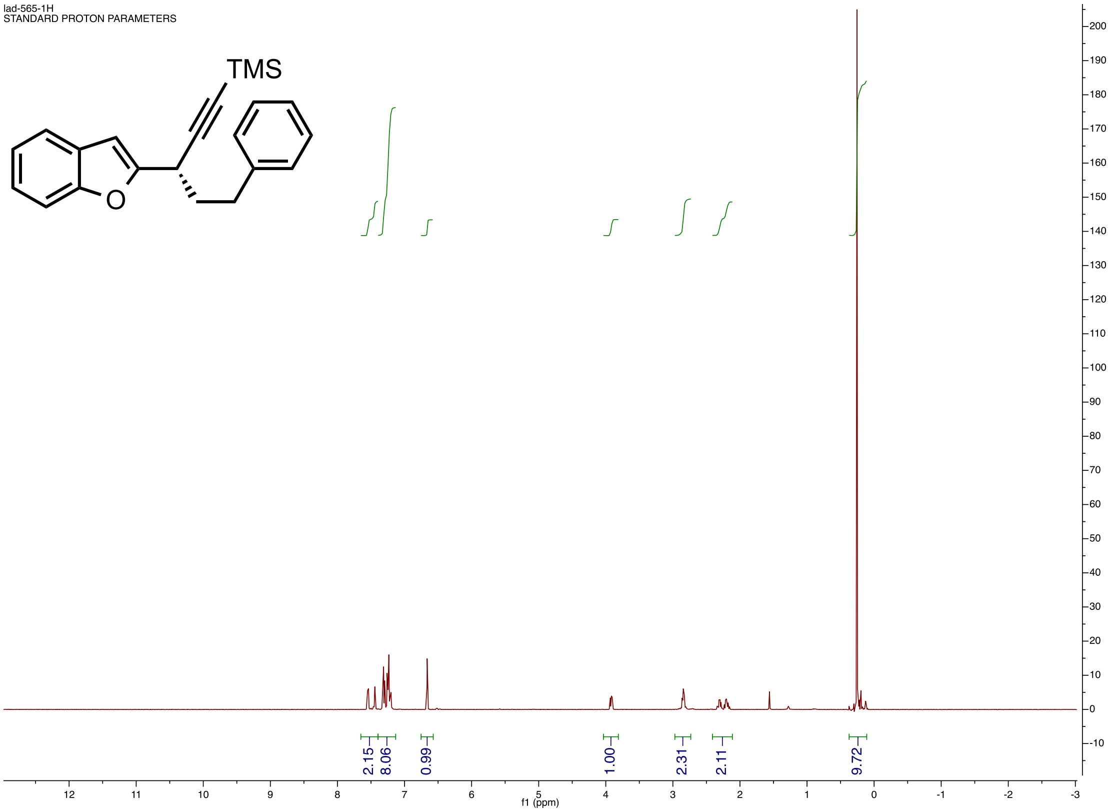
lad-565-1H
STANDARD PROTON PARAMETERS
TMS
O
2.15
8.06
0.99
1.00
2.31
2.11
9.72
f1 (ppm)

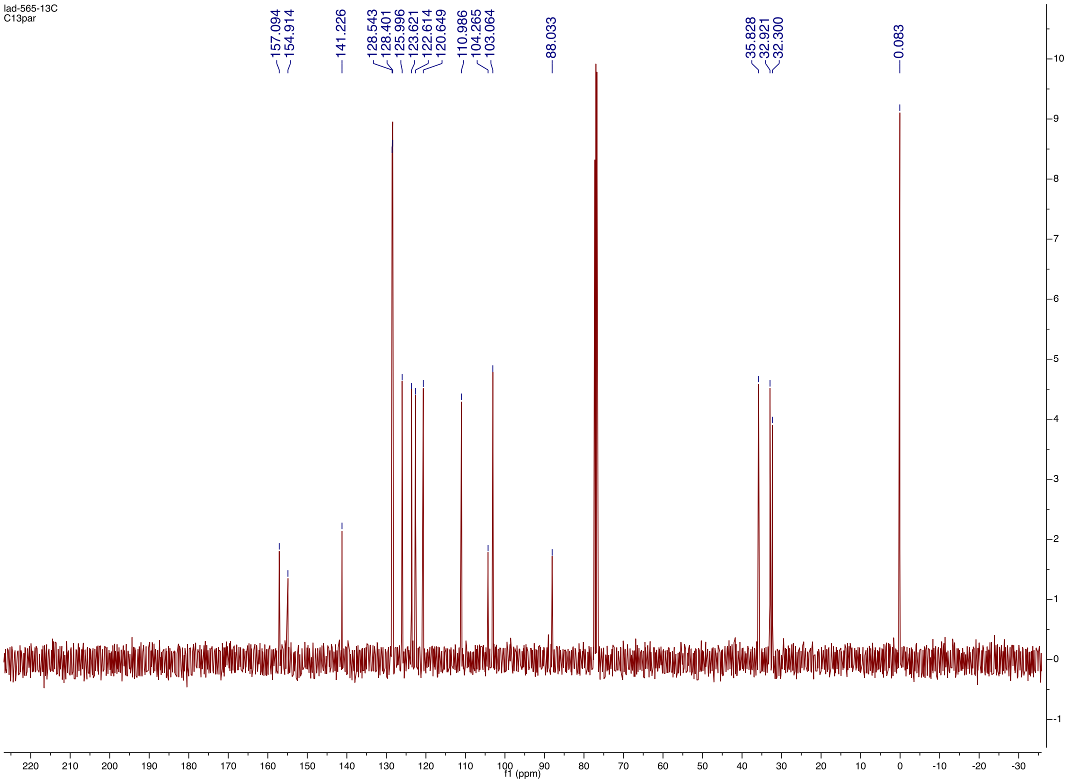
lad-565-13C
C13par
157.094
154.914
141.226
128.543
128.401
125.996
123.621
122.614
120.649
110.986
104.265
103.064
88.033
35.828
32.921
32.300
0.083
f1 (ppm)

Compound 2f

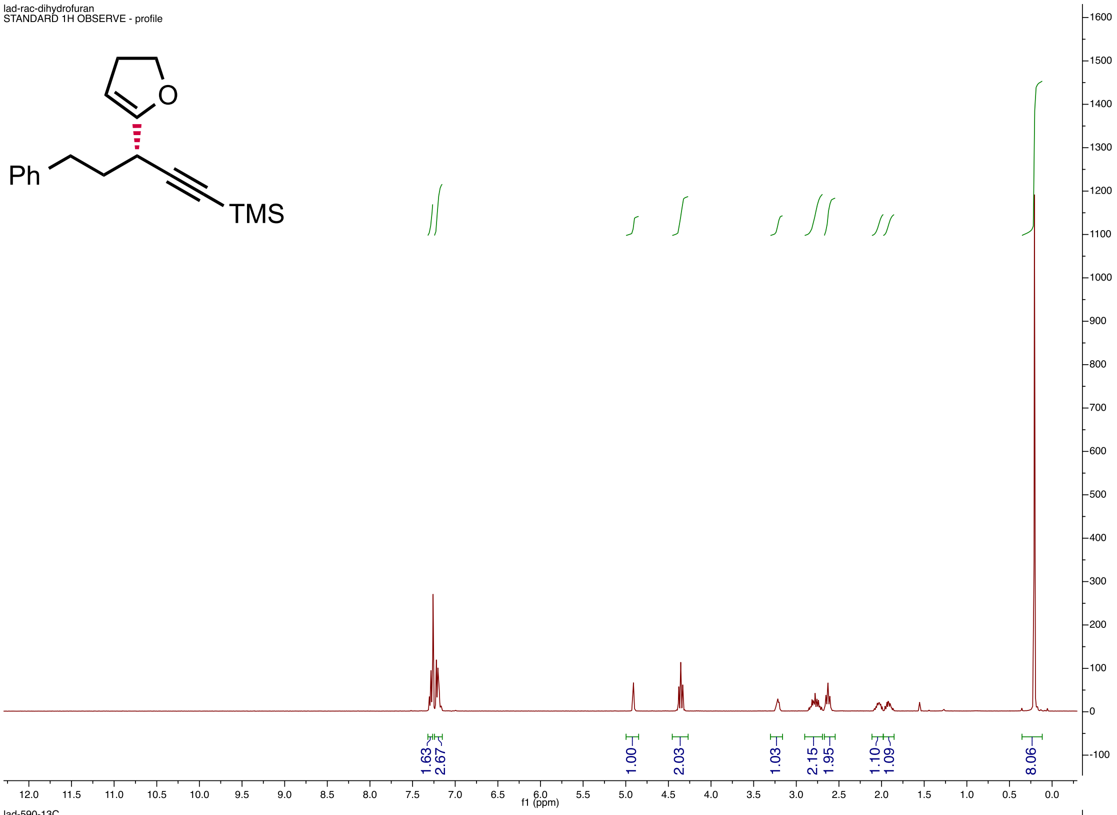

lad-rac-dihydrofuran
STANDARD 1H OBSERVE - profile
Ph
O
TMS
1.63
2.67
1.00
2.03
1.03
2.15
1.95
1.10
1.09
8.06
f1 (ppm)

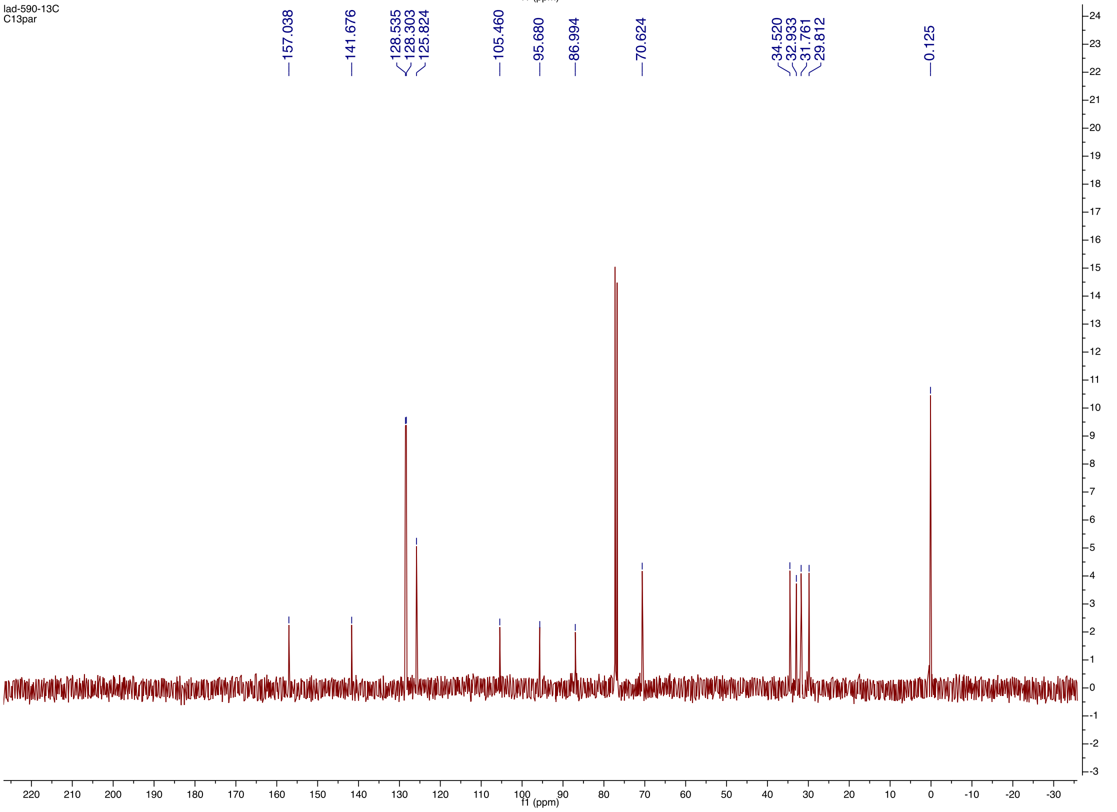

lad-590-13C
C13par
157.038
141.676
128.535
128.303
125.824
105.460
95.680
86.994
70.624
34.520
32.933
31.761
29.812
0.125
f1 (ppm)

Compound 2g

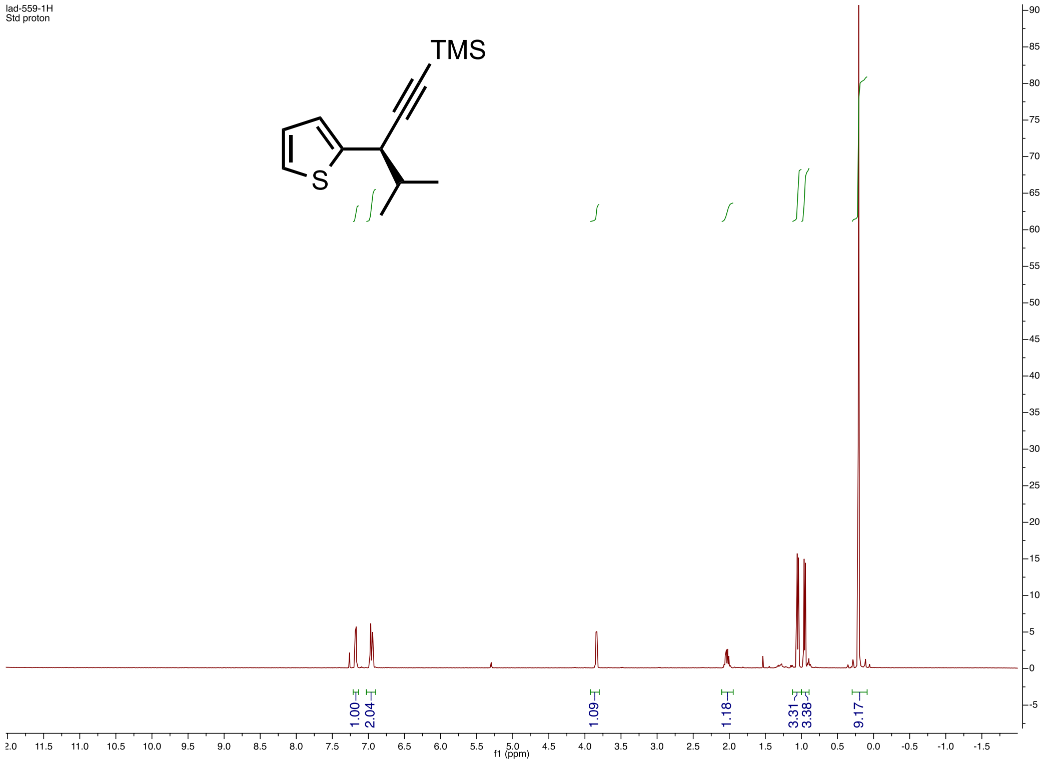
lad-559-1H
Std proton
TMS
S
1.00
2.04
1.09
1.18
3.31
3.38
9.17
f1 (ppm)

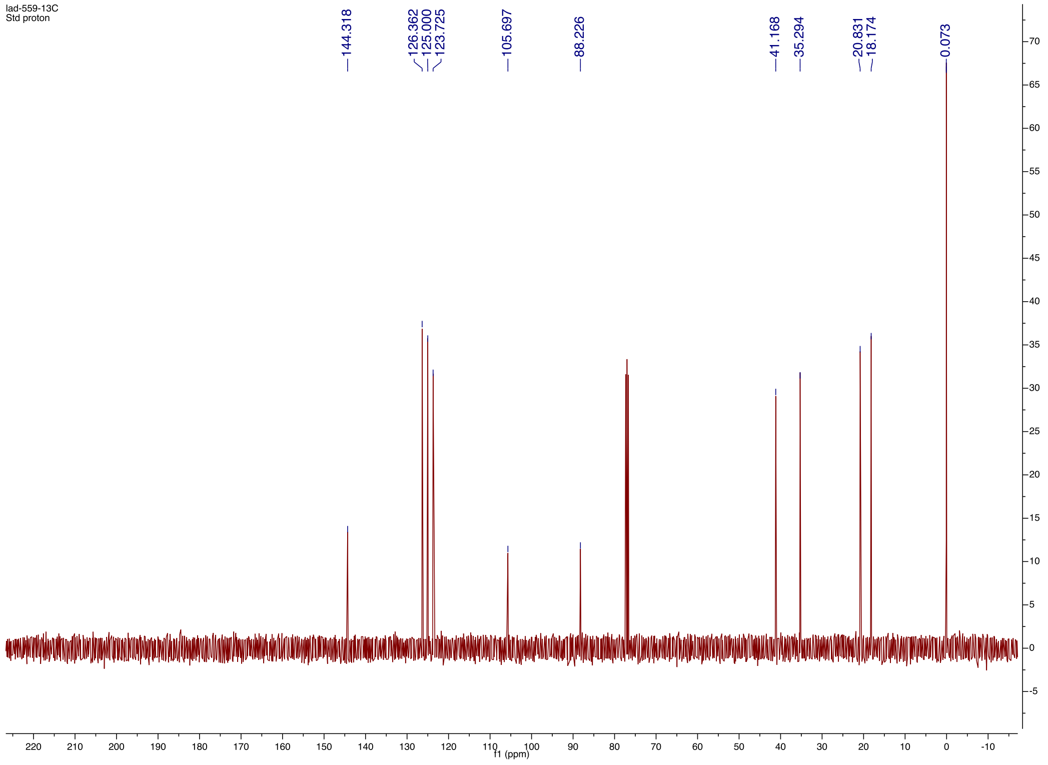
lad-559-13C
Std proton
144.318
126.362
125.000
123.725
105.697
88.226
41.168
35.294
20.831
18.174
0.073
f1 (ppm)

Compound 2h

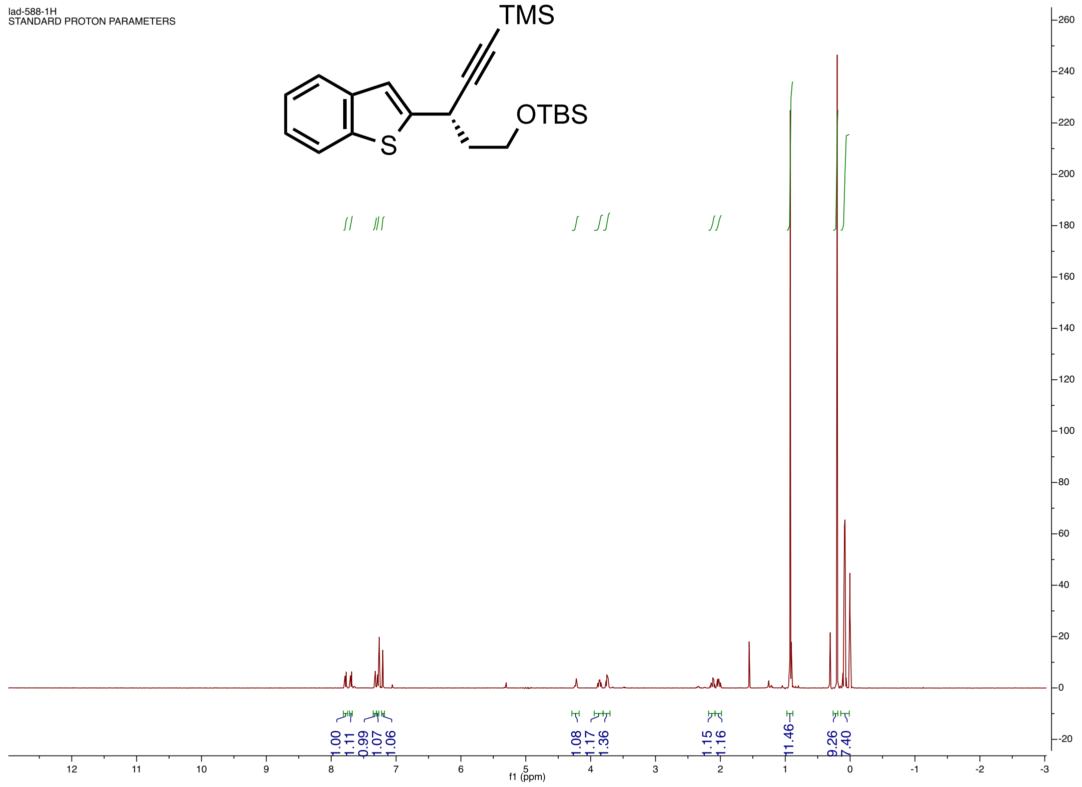
lad-588-1H
STANDARD PROTON PARAMETERS
TMS
OTBS
S
1.00
1.11
0.99
1.07
1.06
1.08
1.17
1.36
1.15
1.16
11.46
9.26
7.40
f1 (ppm)

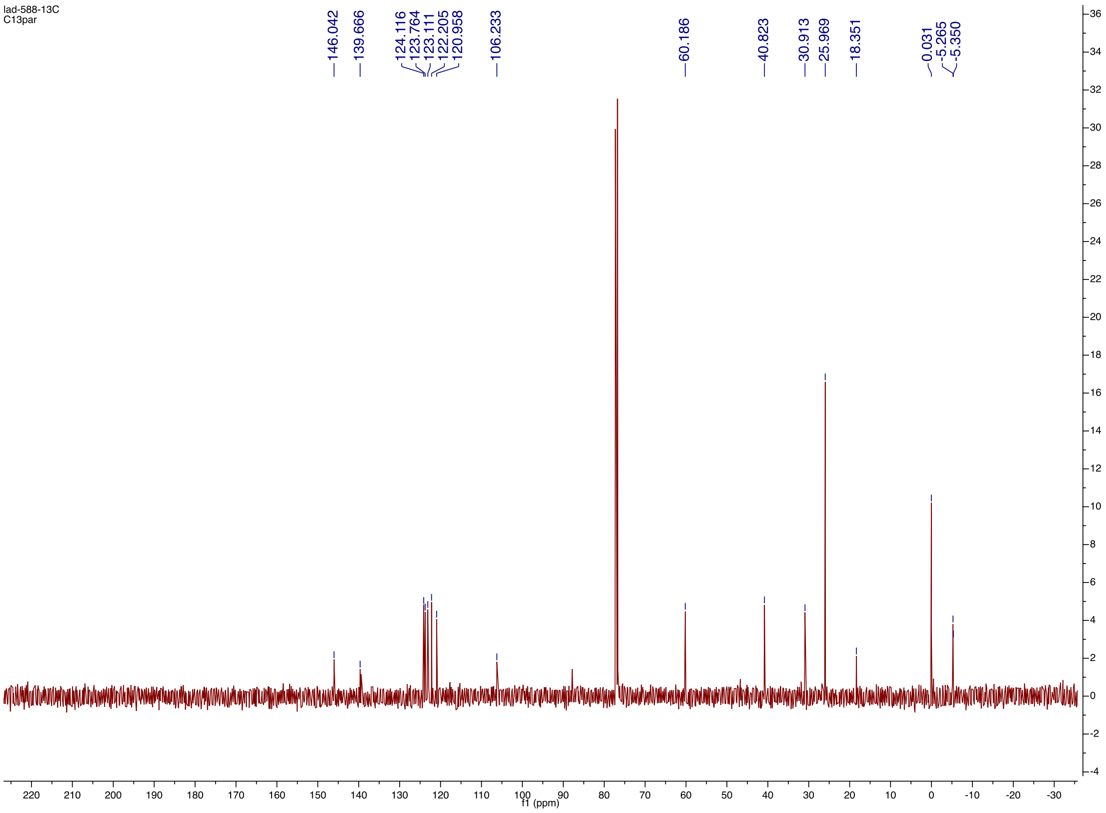
lad-588-13C
C13par
146.042
139.666
124.116
123.764
123.111
122.205
120.958
106.233
60.186
40.823
30.913
25.969
18.351
0.031
-5.265
-5.350
f1 (ppm)

Compound 2i

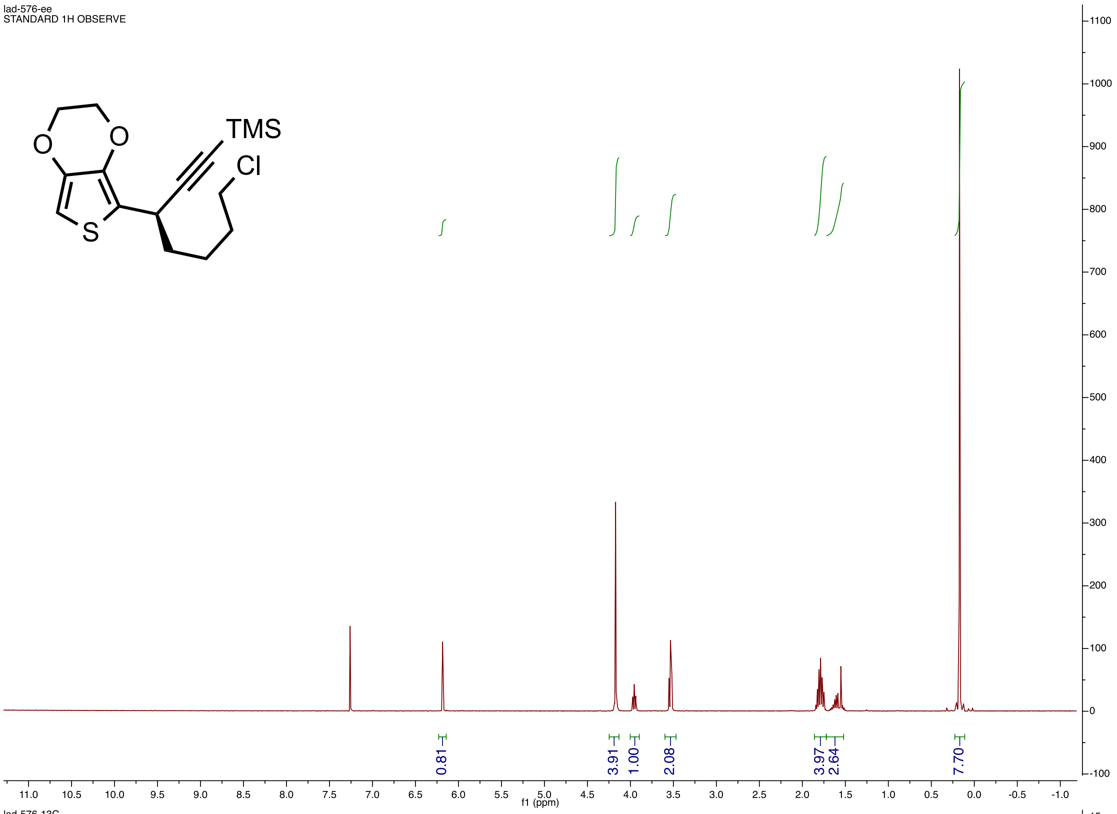
lad-576-ee
STANDARD 1H OBSERVE
TMS
Cl
O
O
S
0.81
3.91
1.00
2.08
3.97
2.64
7.70
f1 (ppm)

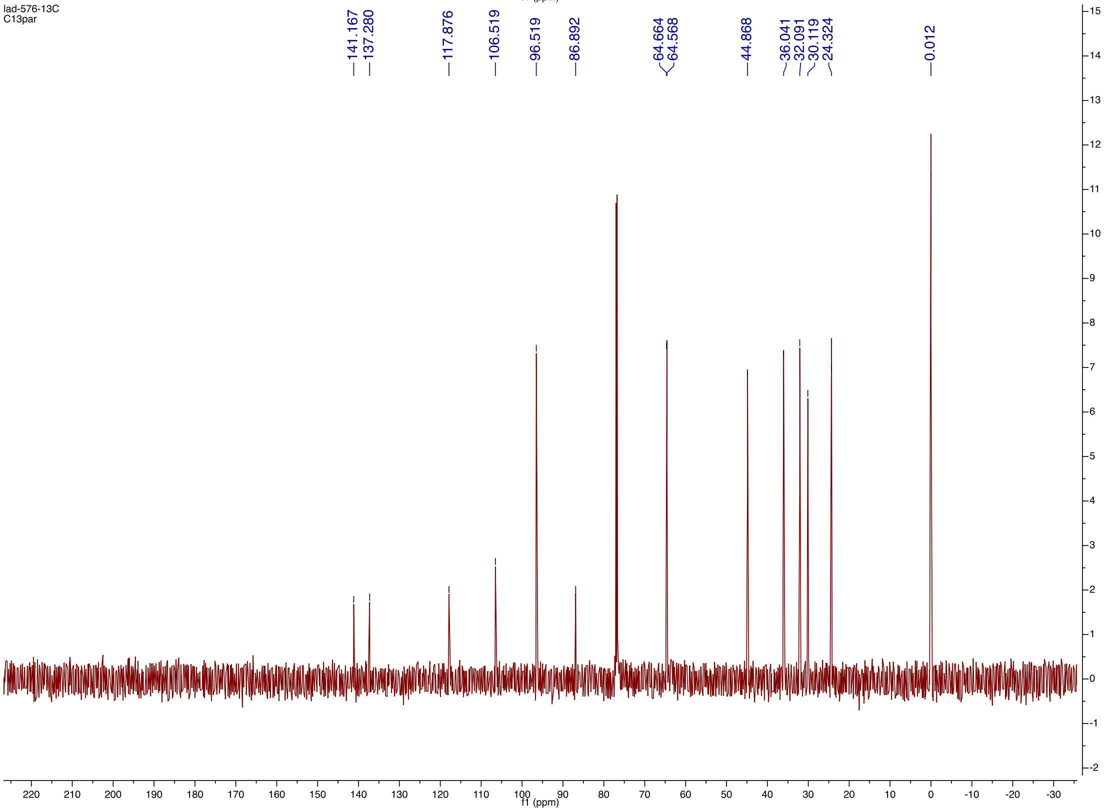
lad-576-13C
C13par
141.167
137.280
117.876
106.519
96.519
86.892
64.664
64.568
44.868
36.041
32.091
30.119
24.324
0.012
f1 (ppm)

Compound 2j

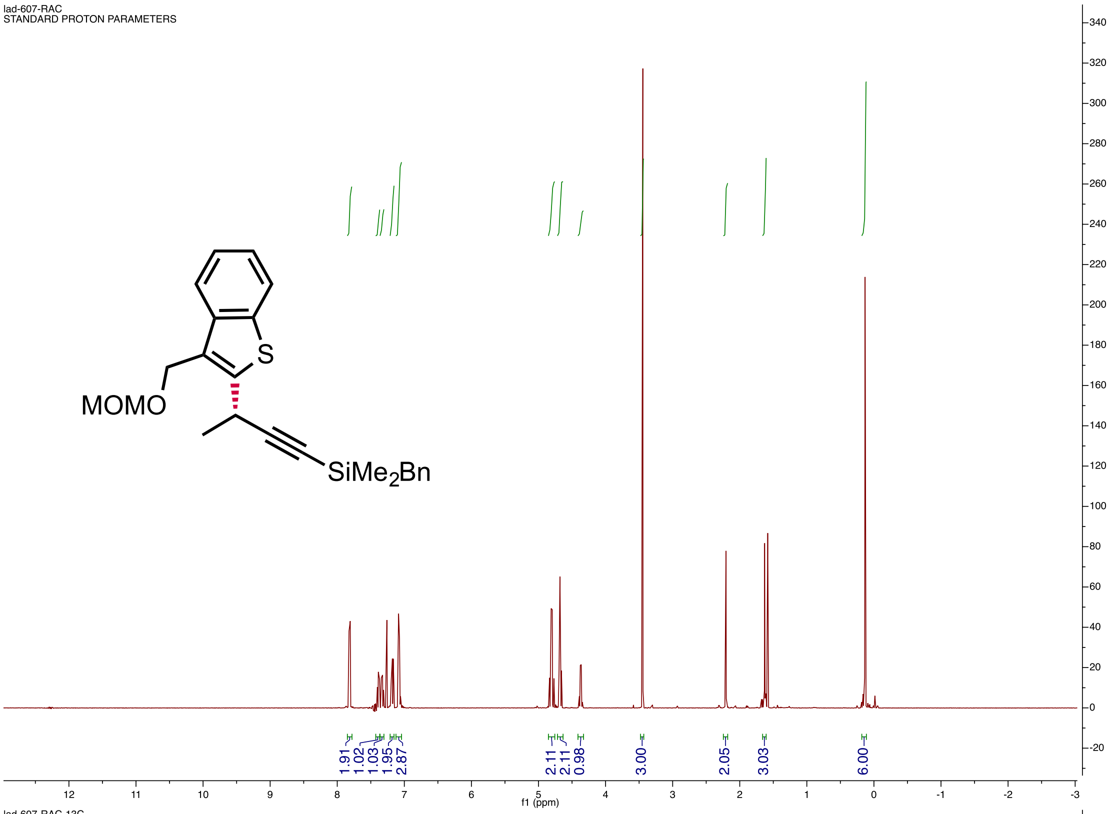
lad-607-RAC
STANDARD PROTON PARAMETERS
S
MOMO
SiMe2Bn
1.91
1.02
1.03
1.95
2.87
2.11
2.11
0.98
3.00
2.05
3.03
6.00
f1 (ppm)

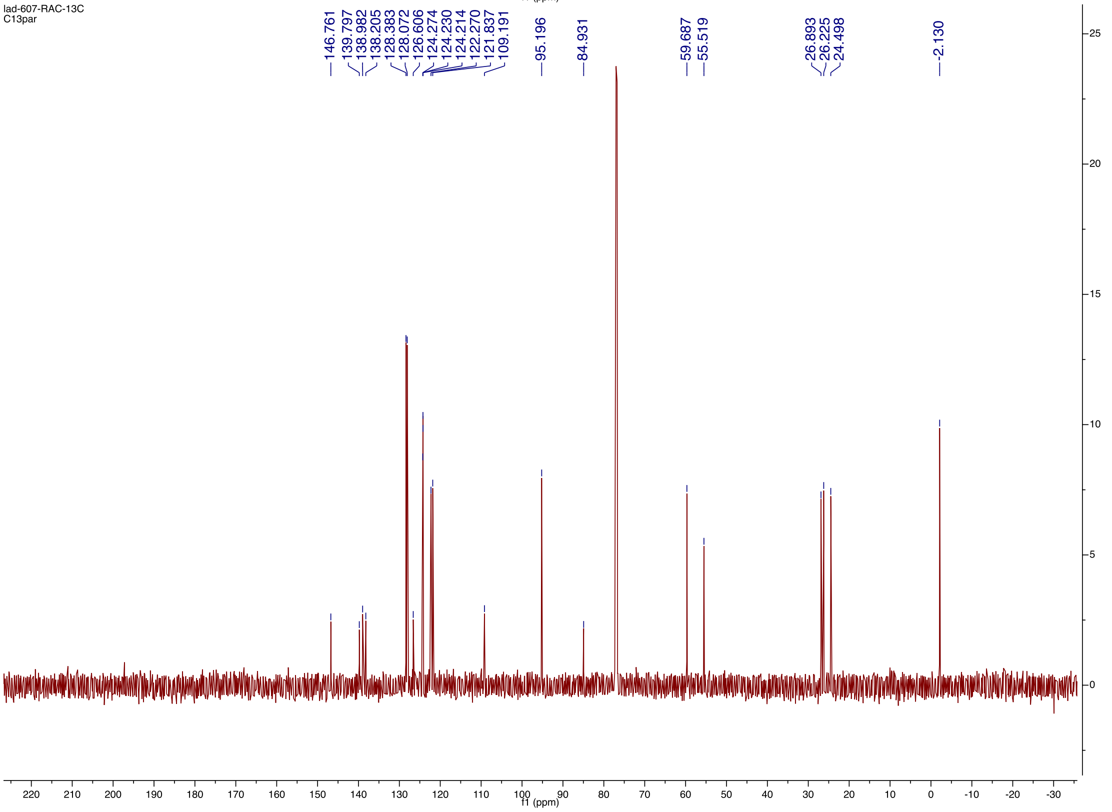
lad-607-RAC-13C
C13par
146.761
139.797
138.982
138.205
128.383
128.072
126.606
124.274
124.230
124.214
122.270
121.837
109.191
95.196
84.931
59.687
55.519
26.893
26.225
24.498
-2.130
f1 (ppm)

Compound 2k

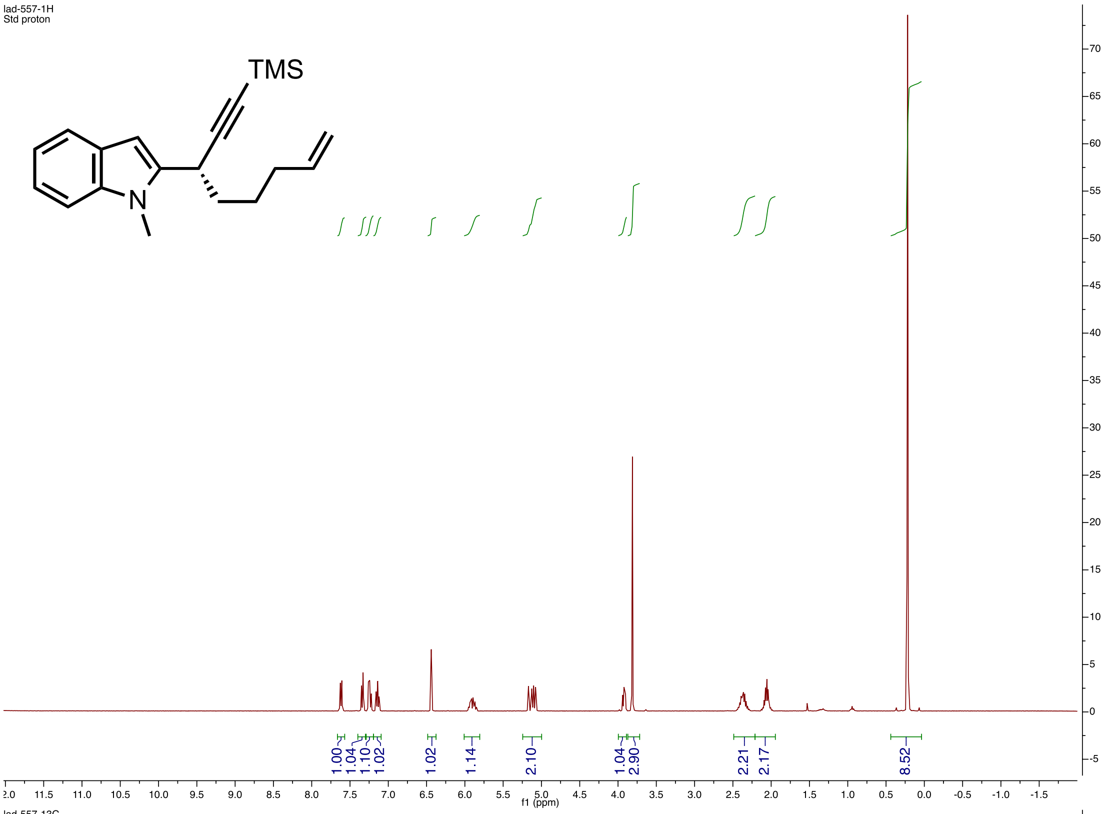
lad-557-1H
Std proton
TMS
N
1.00
1.04
1.10
1.02
1.02
1.14
2.10
1.04
2.90
2.21
2.17
8.52
f1 (ppm)

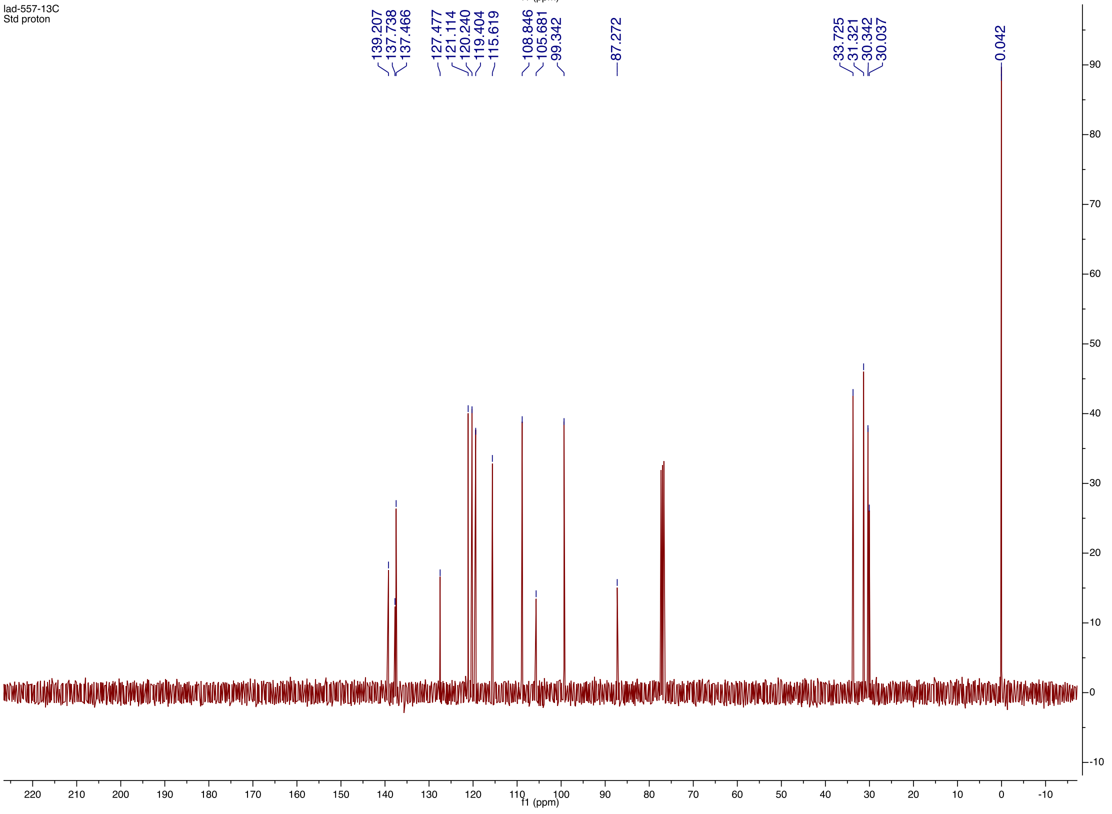
lad-557-13C
Std proton
139.207
137.738
137.466
127.477
121.114
120.240
119.404
115.619
108.846
105.681
99.342
87.272
33.725
31.321
30.342
30.037
0.042
f1 (ppm)

Compound 2l

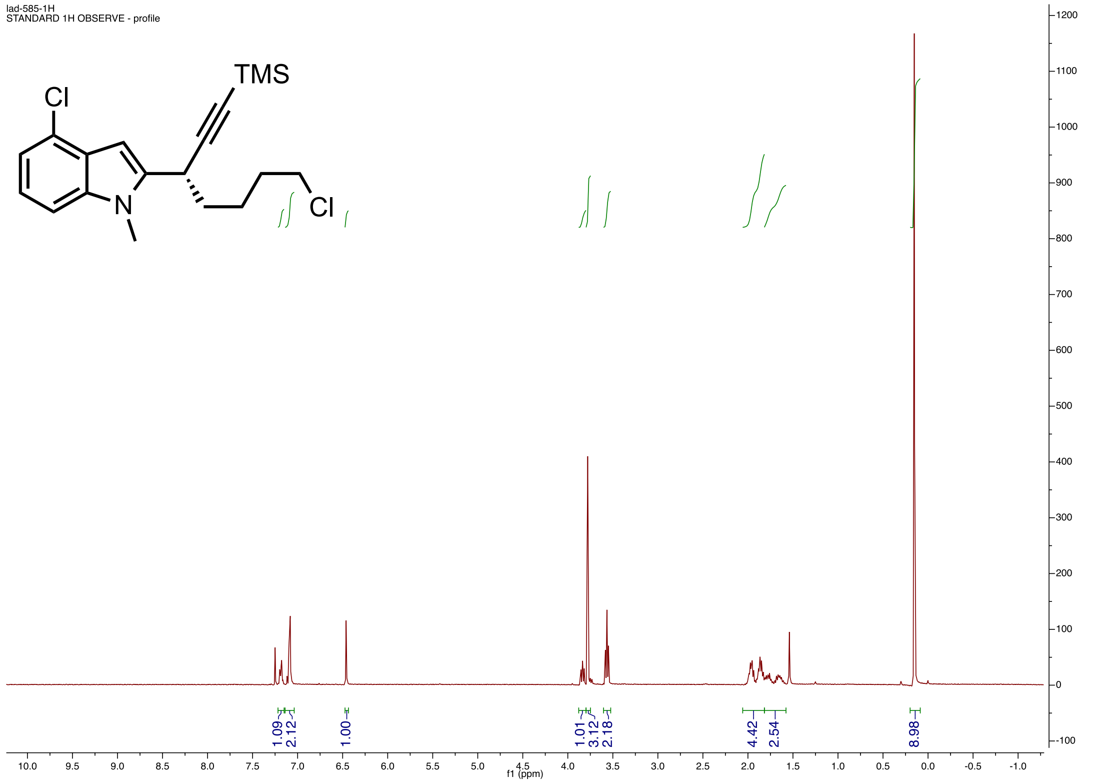
lad-585-1H
STANDARD 1H OBSERVE - profile
TMS
Cl
N
Cl
1.09
2.12
1.00
1.01
3.12
2.18
4.42
2.54
8.98
f1 (ppm)

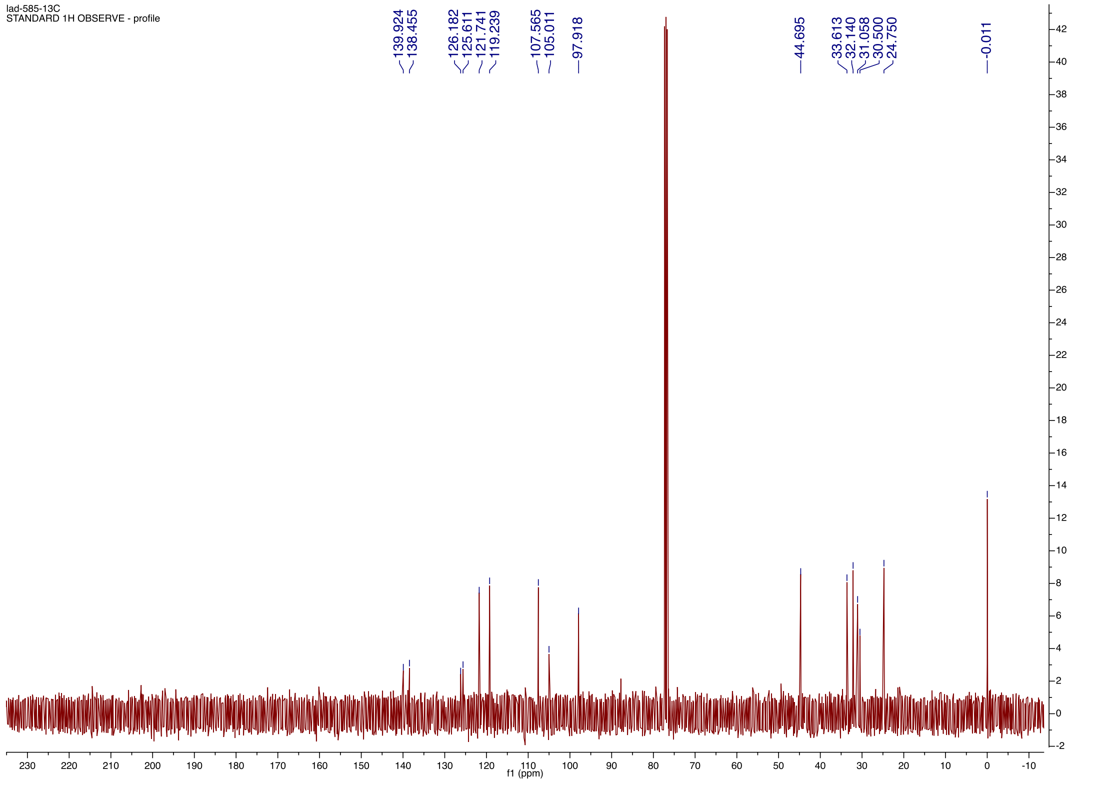
lad-585-13C
STANDARD 1H OBSERVE - profile
139.924
138.455
126.182
125.611
121.741
119.239
107.565
105.011
97.918
44.695
33.613
32.140
31.058
30.500
24.750
-0.011
f1 (ppm)

Compound 2m

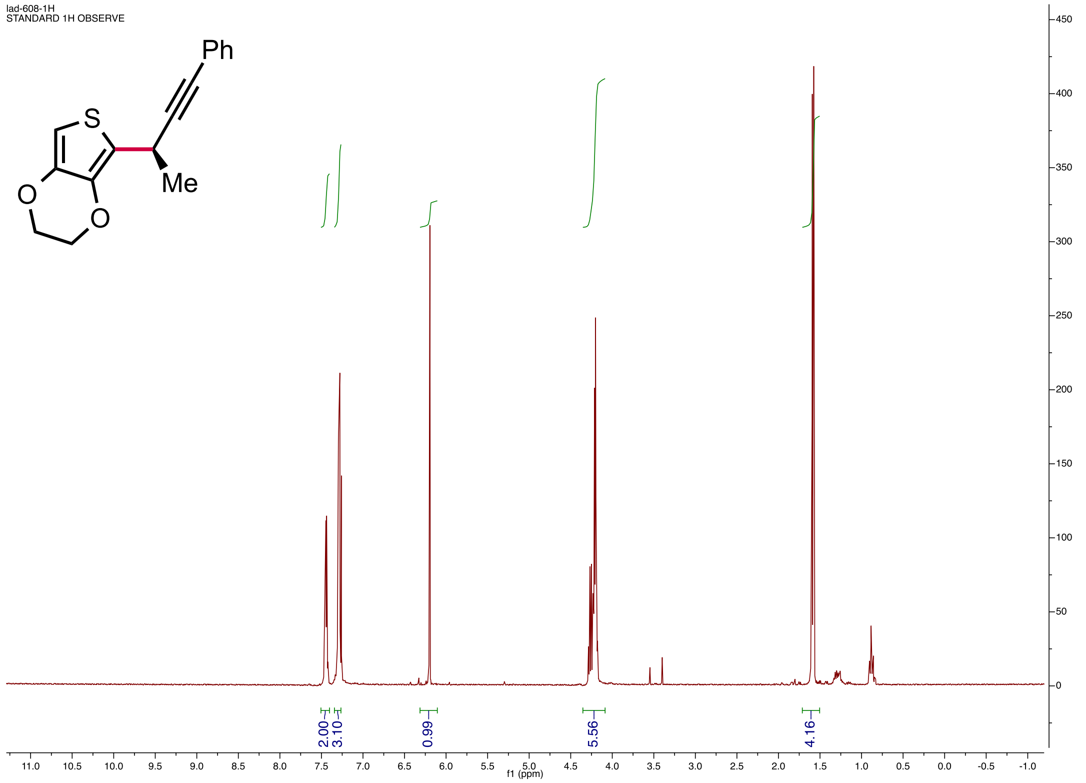
lad-608-1H
STANDARD 1H OBSERVE
Ph
S
O
O
Me
2.00
3.10
0.99
5.56
4.16
f1 (ppm)

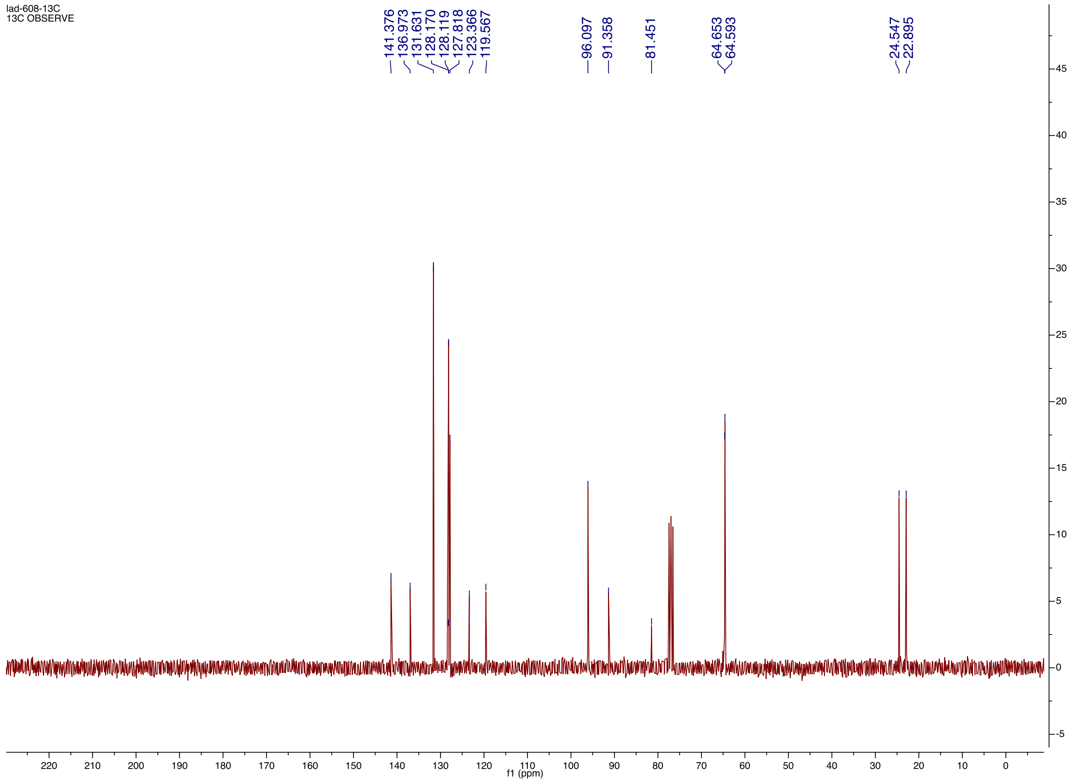
lad-608-13C
13C OBSERVE
141.376
136.973
131.631
128.170
128.119
127.818
123.366
119.567
96.097
91.358
81.451
64.653
64.593
24.547
22.895
f1 (ppm)

Compound 5a

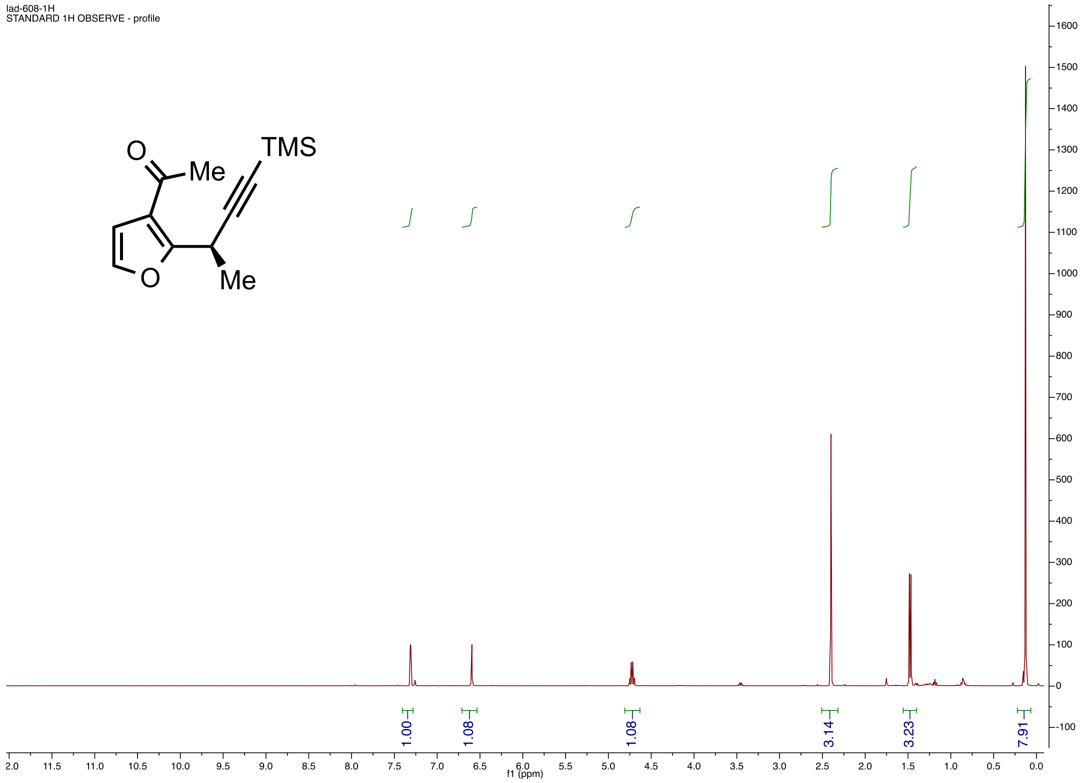

lad-608-1H
STANDARD 1H OBSERVE - profile
O
Me
TMS
O
Me
1.00
1.08
1.08
3.14
3.23
7.91
f1 (ppm)

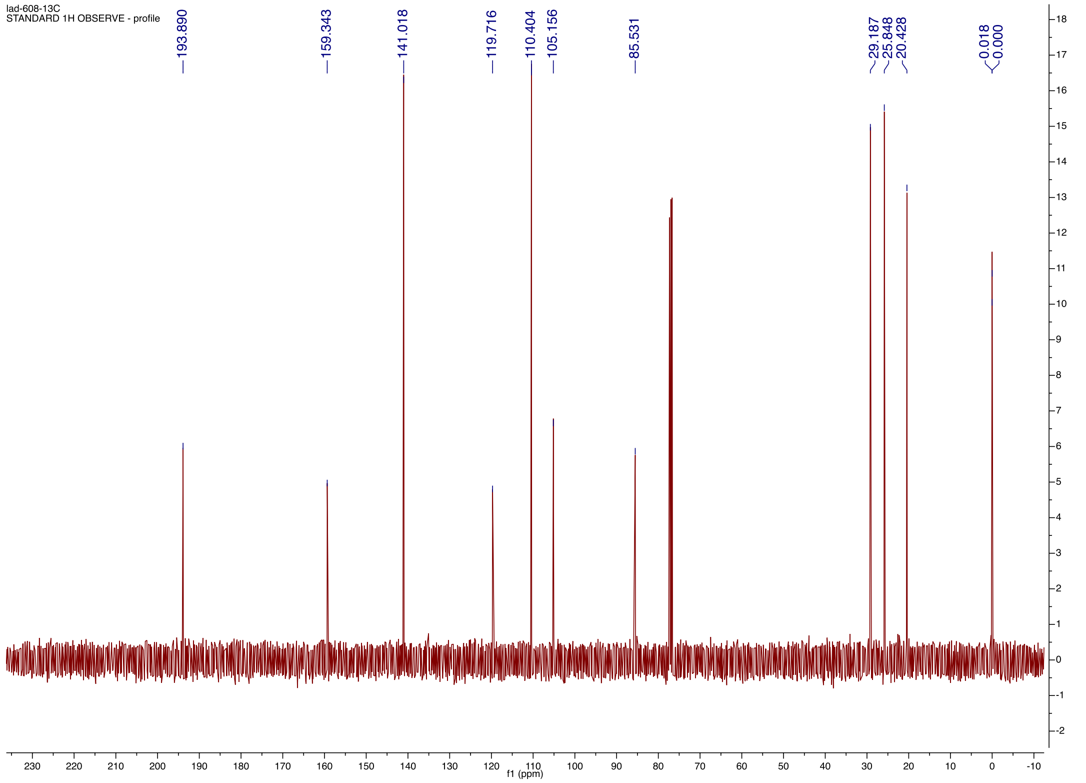

lad-608-13C
STANDARD 1H OBSERVE - profile
193.890
159.343
141.018
119.716
110.404
105.156
85.531
29.187
25.848
20.428
0.018
0.000
f1 (ppm)

Compound 2o dr = 1:1

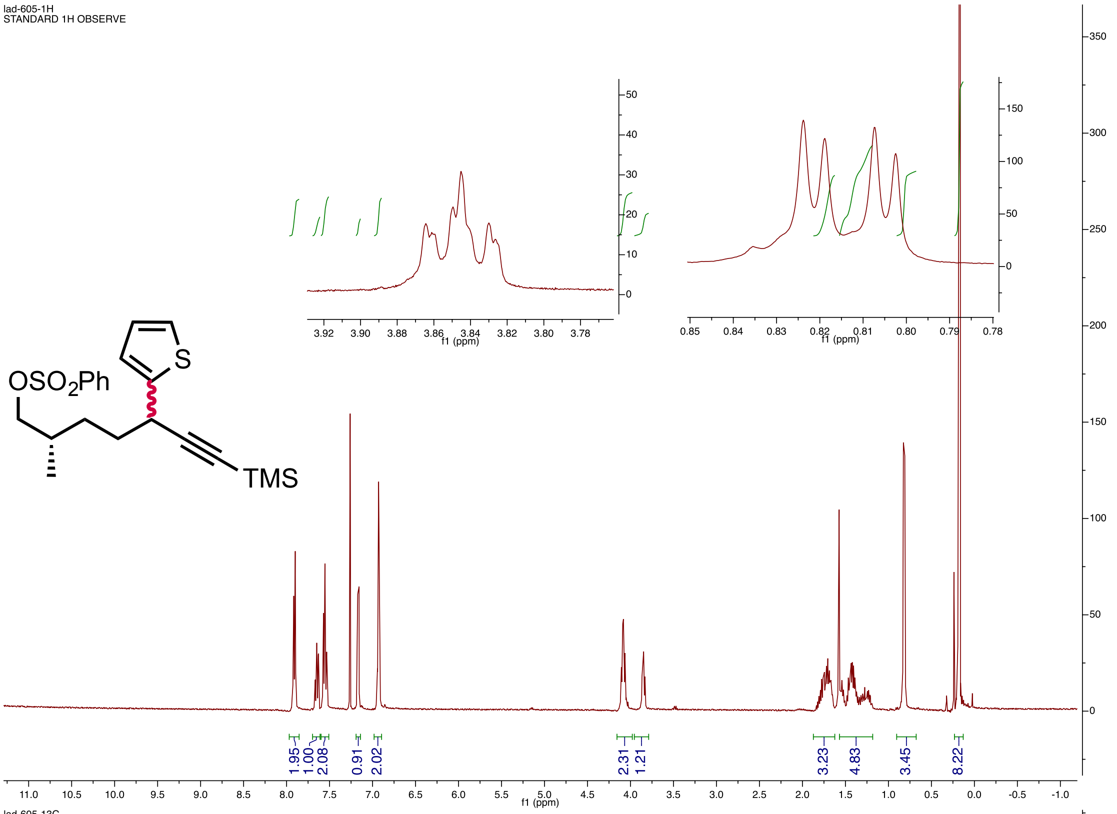

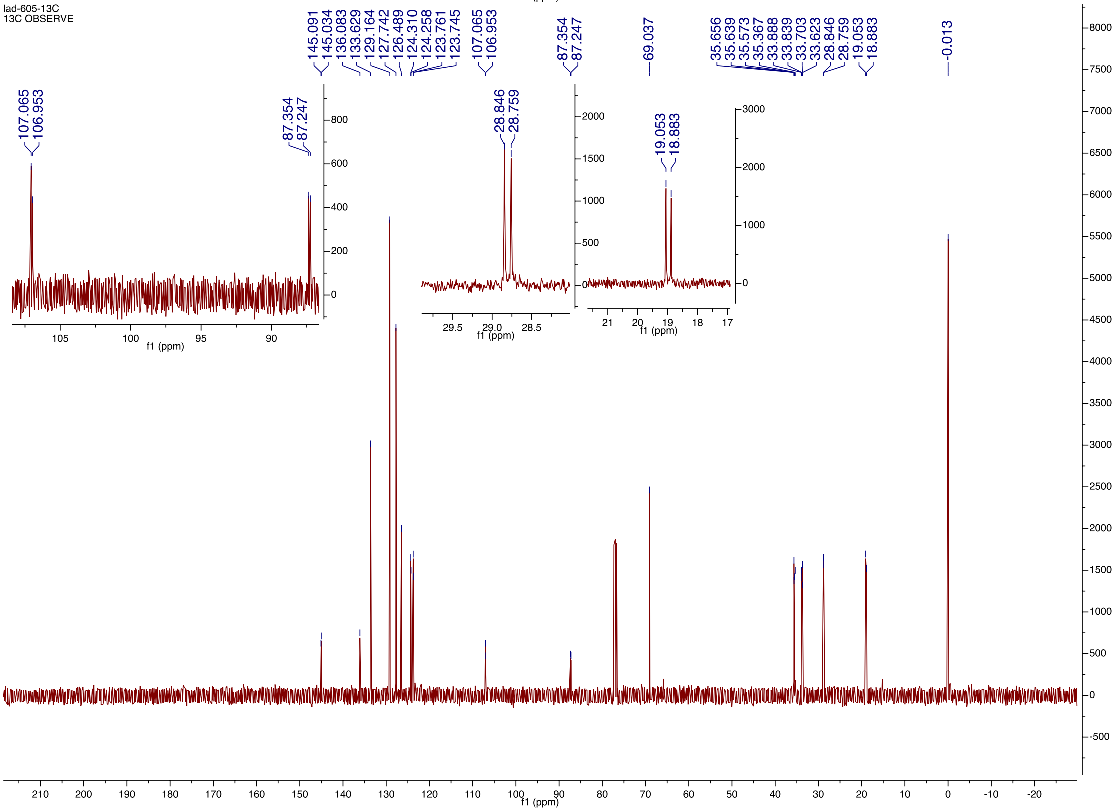

Compound 2o dr > 95:5

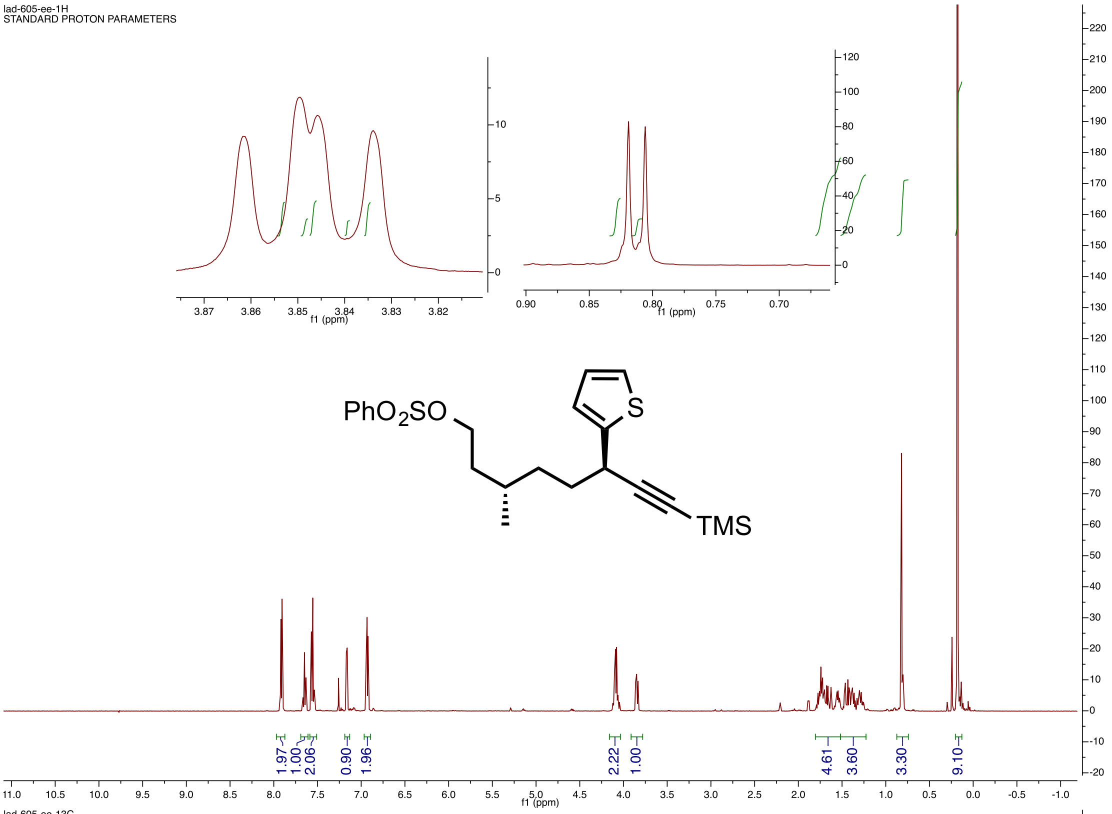

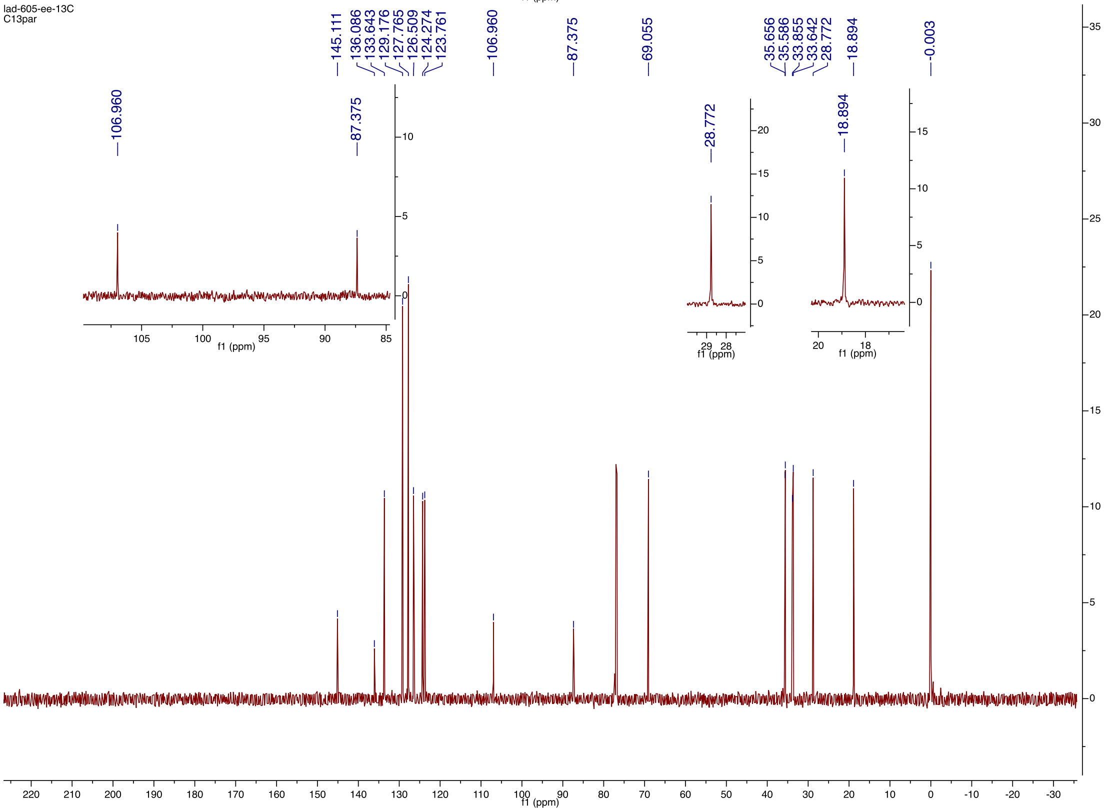

Compound 5b dr = 1:1

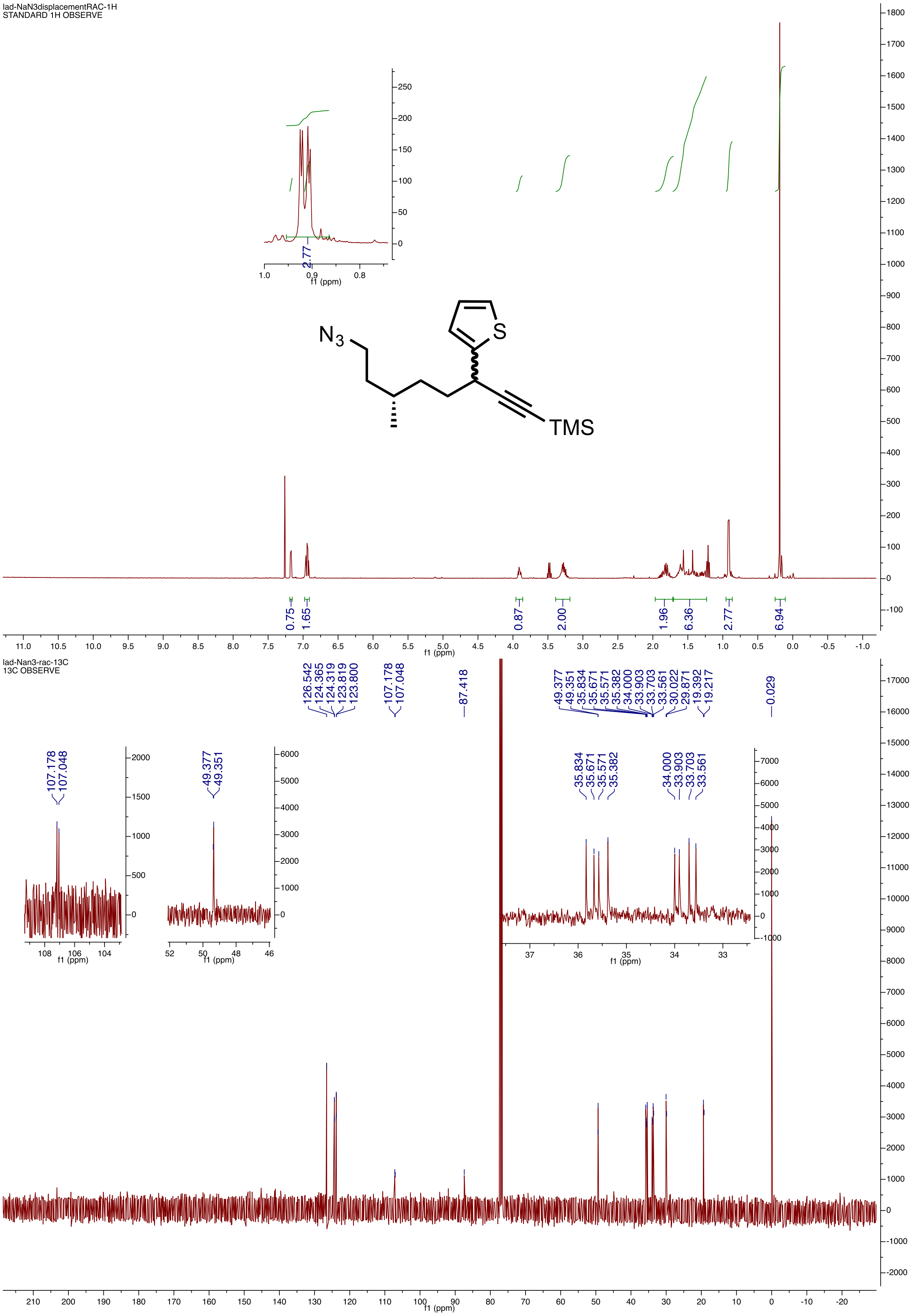

Compound 5b dr > 95:5

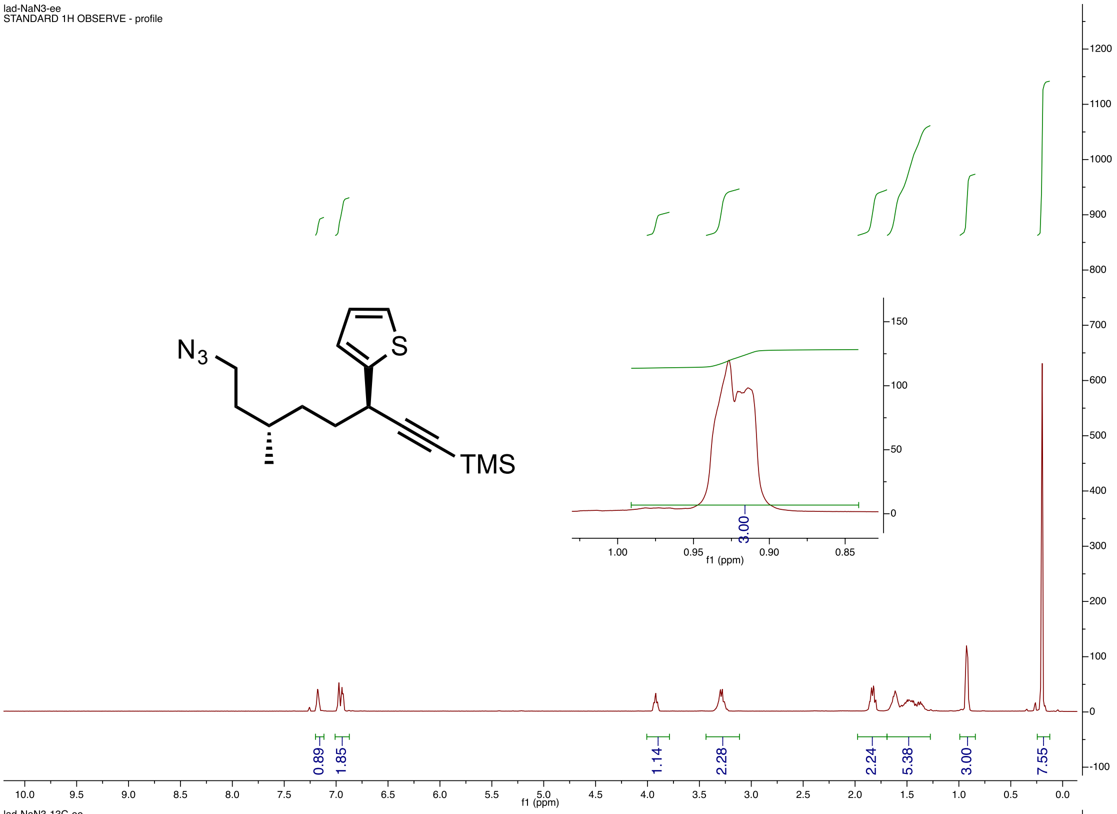

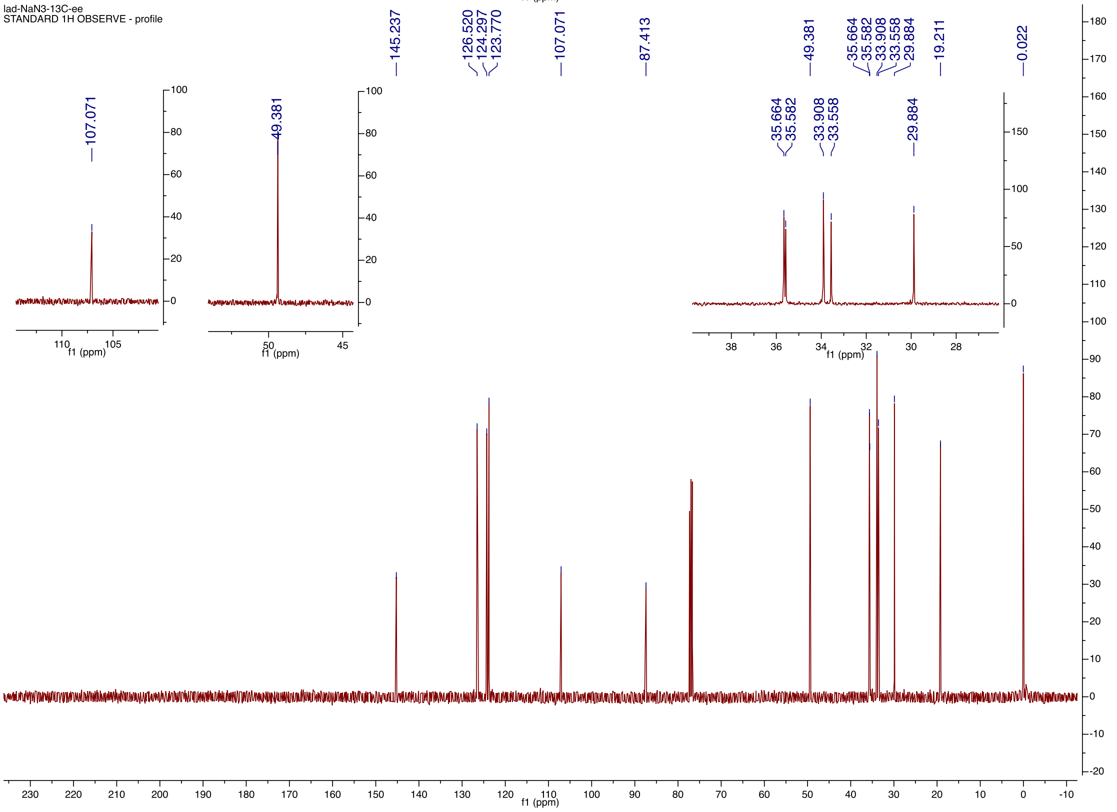

Compound 2p

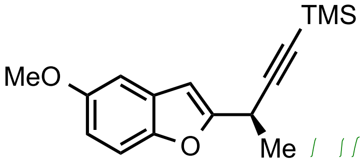

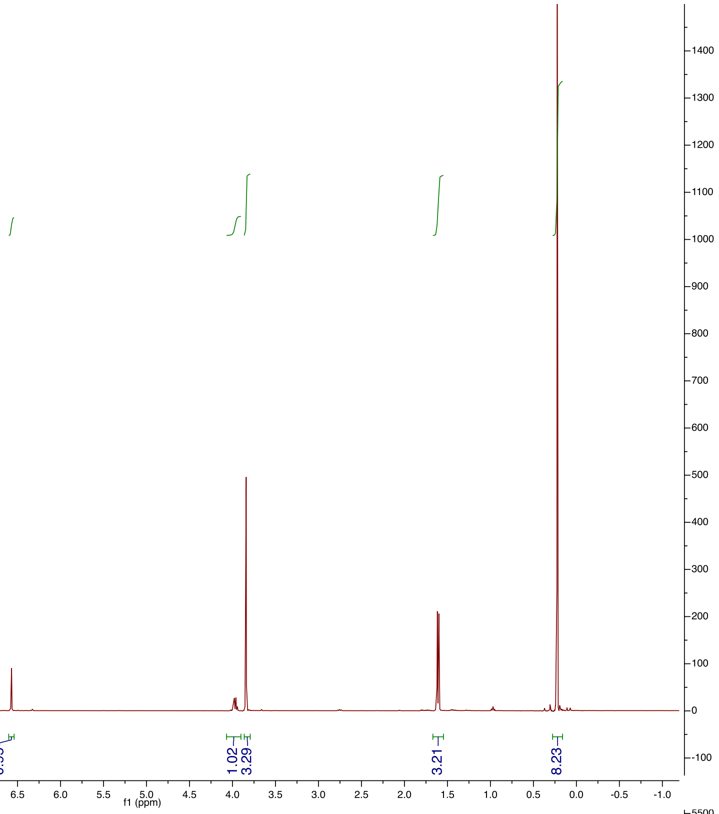
lad-604-1H
STANDARD 1H OBSERVE
1.00
0.98
1.07
0.95
1.02
3.29
3.21
8.23
f1 (ppm)

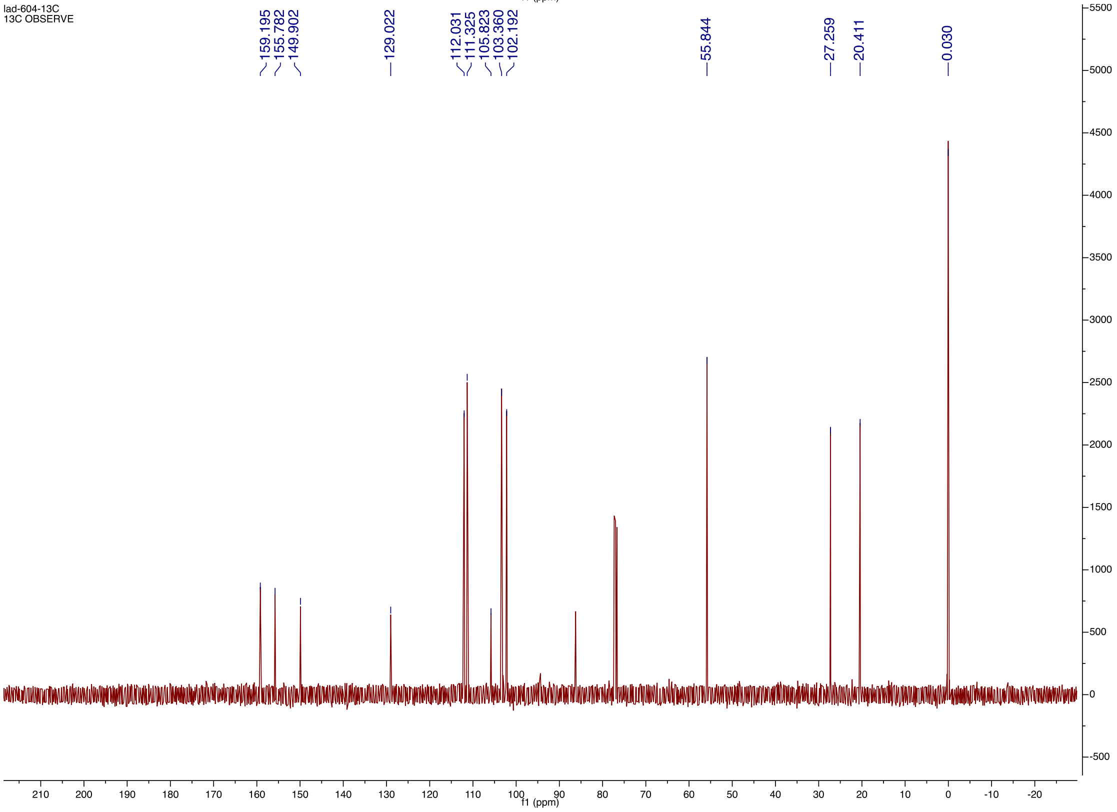
lad-604-13C
13C OBSERVE
159.195
155.782
149.902
129.022
112.031
111.325
105.823
103.360
102.192
55.844
27.259
20.411
0.030
f1 (ppm)

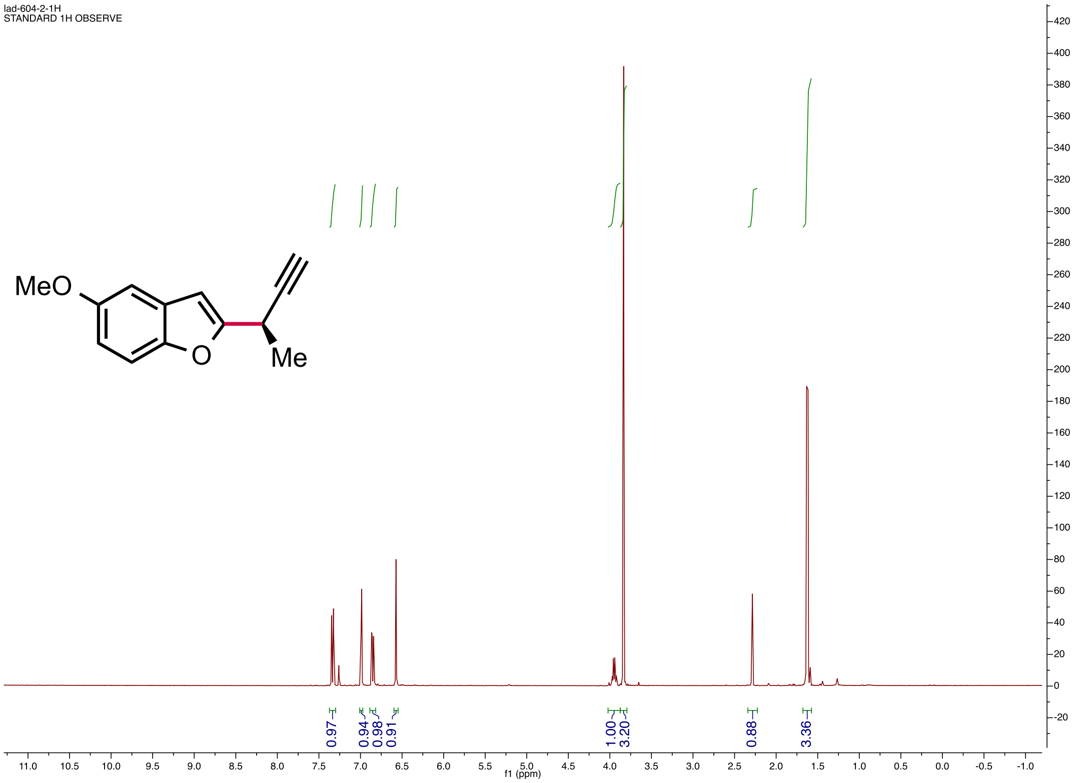
lad-604-2-1H
STANDARD 1H OBSERVE
MeO
O
Me
0.97
0.94
0.98
0.91
1.00
3.20
0.88
3.36
f1 (ppm)

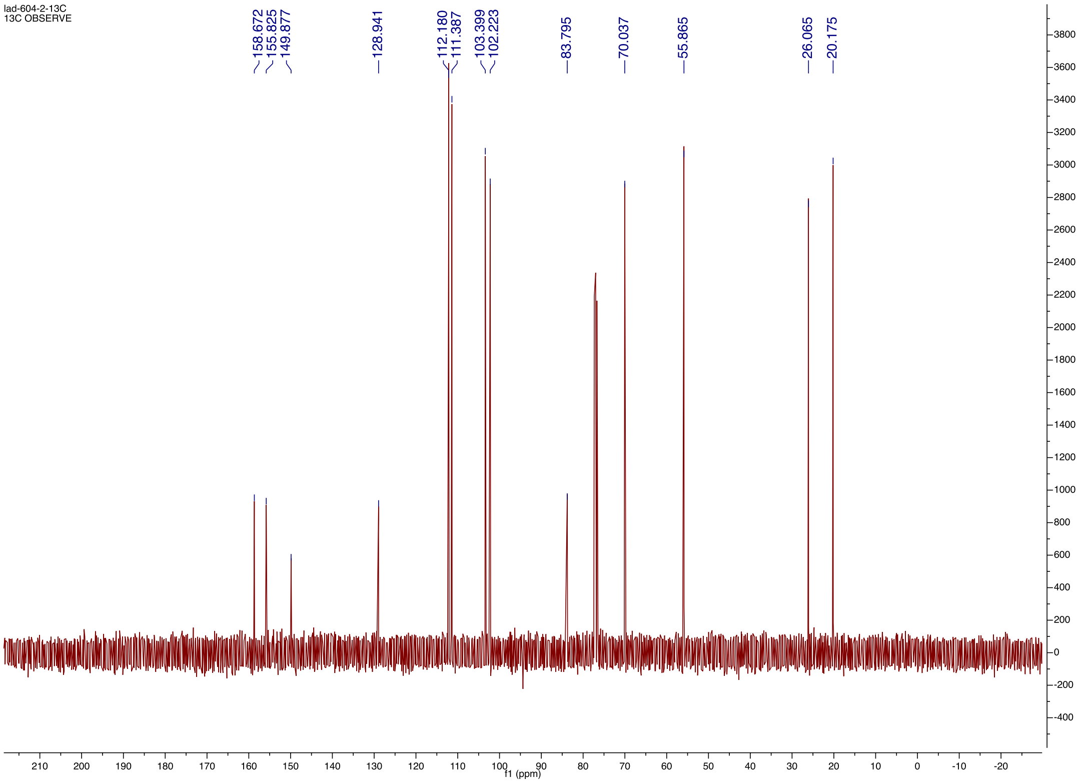
lad-604-2-13C
13C OBSERVE
158.672
155.825
149.877
128.941
112.180
111.387
103.399
102.223
83.795
70.037
55.865
26.065
20.175
f1 (ppm)

Compound 5c

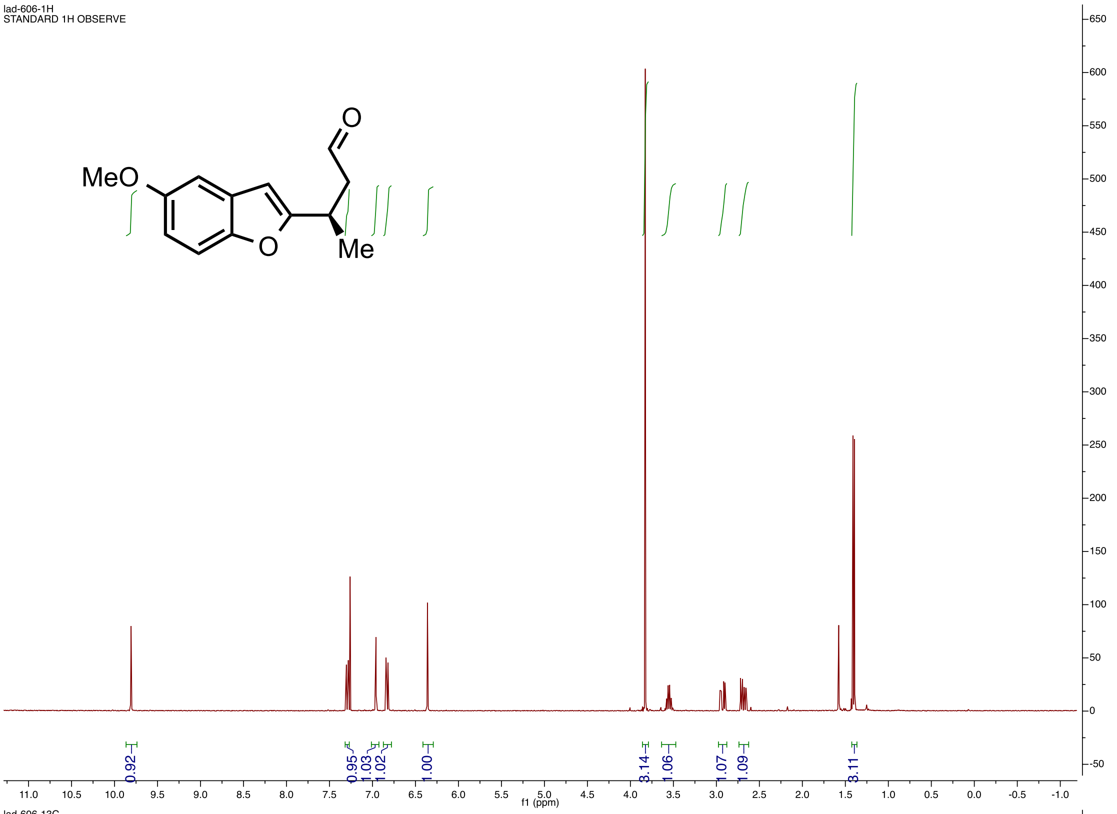
lad-606-1H
STANDARD 1H OBSERVE
MeO
O
O
Me
0.92
0.95
1.03
1.02
1.00
3.14
1.06
1.07
1.09
3.11
f1 (ppm)

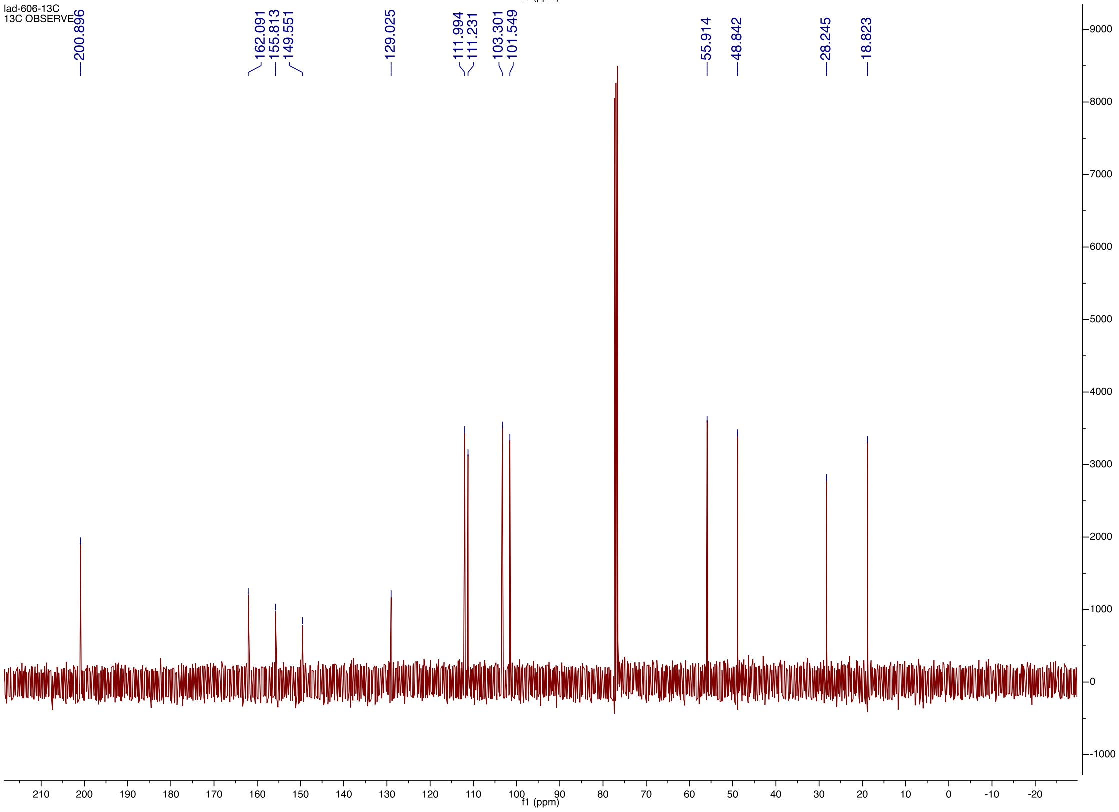
lad-606-13C
13C OBSERVE
200.896
162.091
155.813
149.551
129.025
111.994
111.231
103.301
101.549
55.914
48.842
28.245
18.823
f1 (ppm)
